# Mobile Colistin Resistance (*mcr*) Gene-Containing Organisms in Poultry Sector in Low- and Middle-Income Countries: Epidemiology, Characteristics, and One Health Control Strategies

**DOI:** 10.3390/antibiotics12071117

**Published:** 2023-06-28

**Authors:** Madubuike Umunna Anyanwu, Ishmael Festus Jaja, Charles Odilichukwu R. Okpala, Emmanuel Okechukwu Njoga, Nnenna Audrey Okafor, James Wabwire Oguttu

**Affiliations:** 1 Department of Livestock and Pasture Science, University of Fort Hare, Alice 5700, South Africa; 2 Department of Functional Food Products Development, Faculty of Biotechnology and Food Science, Wrocław University of Environmental and Life Sciences, 50-375 Wrocław, Poland; charles.okpala@upwr.edu.pl; 3 UGA Cooperative Extension, College of Agricultural and Environmental Sciences, University of Georgia, Athens, GA 30602, USA; 4 Department of Veterinary Public Health and Preventive Medicine, University of Nigeria, Nsukka 400001, Nigeria; njoga.emmanuel@unn.edu.ng; 5 Department of Microbiology, University of Nigeria, Nsukka 400001, Nigeria; nnenna.okafor.pg04212@unn.edu.ng; 6 Department of Agriculture and Animal Health, Florida Campus, University of South Africa, Johannesburg 1709, South Africa; joguttu@unisa.ac.za

**Keywords:** mobile colistin resistance, low- and middle-income countries, *mcr* gene, One Health, tigecycline resistance, poultry sector

## Abstract

Mobile colistin resistance (*mcr*) genes (*mcr*-1 to *mcr*-10) are plasmid-encoded genes that threaten the clinical utility of colistin (COL), one of the highest-priority critically important antibiotics (HP-CIAs) used to treat infections caused by multidrug-resistant and extensively drug-resistant bacteria in humans and animals. For more than six decades, COL has been used largely unregulated in the poultry sector in low- and middle-income countries (LMICs), and this has led to the development/spread of *mcr* gene-containing bacteria (MGCB). The prevalence rates of *mcr*-positive organisms from the poultry sector in LMICs between January 1970 and May 2023 range between 0.51% and 58.8%. Through horizontal gene transfer, conjugative plasmids possessing insertion sequences (ISs) (especially IS*Apl1*), transposons (predominantly Tn*6330*), and integrons have enhanced the spread of *mcr*-1, *mcr*-2, *mcr*-3, *mcr*-4, *mcr*-5, *mcr*-7, *mcr*-8, *mcr*-9, and *mcr*-10 in the poultry sector in LMICs. These genes are harboured by *Escherichia*, *Klebsiella*, *Proteus*, *Salmonella*, *Cronobacter*, *Citrobacter*, *Enterobacter*, *Shigella*, *Providencia*, *Aeromonas*, *Raoultella*, *Pseudomonas*, and *Acinetobacter* species, belonging to diverse clones. The *mcr*-1, *mcr*-3, and *mcr*-10 genes have also been integrated into the chromosomes of these bacteria and are mobilizable by ISs and integrative conjugative elements. These bacteria often coexpress *mcr* with virulence genes and other genes conferring resistance to HP-CIAs, such as extended-spectrum cephalosporins, carbapenems, fosfomycin, fluoroquinolone, and tigecycline. The transmission routes and dynamics of MGCB from the poultry sector in LMICs within the One Health triad include contact with poultry birds, feed/drinking water, manure, poultry farmers and their farm workwear, farming equipment, the consumption and sale of contaminated poultry meat/egg and associated products, etc. The use of pre/probiotics and other non-antimicrobial alternatives in the raising of birds, the judicious use of non-critically important antibiotics for therapy, the banning of nontherapeutic COL use, improved vaccination, biosecurity, hand hygiene and sanitization, the development of rapid diagnostic test kits, and the intensified surveillance of *mcr* genes, among others, could effectively control the spread of MGCB from the poultry sector in LMICs.

## 1. Introduction

Bacterial antimicrobial resistance (AMR) facilitated by mobile genetic elements (MGEs), especially plasmids, poses a major risk to global human and animal health, as well as the economy. Plasmid-mediated AMR negatively impacts food security and safety and can potentially imperil the attainment of sustainable development goals (SDGs) 1, 2, 3, 6, 8, 10, 12, 13, and 16 by 2030, especially in low- and middle-income countries (LMICs) [1,2]. In 2019 alone, 127 million people, most of whom resided in LMICs, died globally due to antimicrobial-resistant bacterial infections, and the mortality rate is poised to reach 10 million by 2050 if the problem of AMR (especially plasmid-mediated AMR) is unaddressed [2,3].

The importance of poultry in providing relatively cheap nutrition for the global population and its economic benefits to different countries, especially LMICs, are well recognized [4,5]. Poultry meat is high in quality protein and other nutrients and is accepted across diverse socioeconomic and religious cultures. Hence, it is expected that, due to the rising global population, outbreaks of African swine fever that strongly constrain pork production, and the impact of the COVID-19 pandemic, poultry will be the most important sector of meat production by 2025 [5,6,7]. The rising global population is expected to increase the demand for meat products worldwide and lead to a subsequent rise in poultry production [6,7,8]. The rise in poultry could lead to the increased use, misuse, and abuse of antimicrobials, including HP-CIAs such as COL, in animal agriculture in most LMICs [9]. Consequently, if no action is taken, there could be increased cases of antimicrobial-resistance-associated human and animal deaths.

Currently, LMICs are made up of 136 countries/territories. Most of these countries are in Africa, Asia, and South America and are among the world’s major producers/exporters of poultry products [9,10]. LMICs are high-impact antimicrobial resistance (AMR) regions contributing significantly to the global pool of AMR genes and difficult-to-treat-infections due to various reasons, including weak national drug-regulatory authorities, inefficient antibiotic policies, and the overuse and misuse of antibiotics [11,12,13]. In LMICs, antimicrobials, including those critical for managing infections associated with multidrug- and extensively drug-resistant organisms, are heavily consumed by livestock, especially poultry [12,14,15]. The increasing populations and economies in LMICs are expected to cause an increased demand for animal protein (up to a 14% increase in global meat consumption by 2030) with a parallel increase in the intensification of poultry systems, which could result in a 65–67% increase in antimicrobial usage from 2010 to 2030 [15,16]. Globally, in 2017, an estimated 93,309 tonnes of antibiotics was sold for use (mostly in LMICs) in food-producing animals (FPAs), and this figure is poised to reach 104,079 tonnes by 2030 [16]. It is projected that if antibiotic consumption keeps growing at the current rates, especially in LMICs, a 2.7-fold global increase in the antimicrobial resistance gene (ARG) abundance of clinically relevant plasmids may be reached by 2030 [17]. Unfortunately, weak regulations, a lack of political will, and inadequate diagnostic infrastructure limit the ability of LMICs to control AMR. In addition, the inability of LMICs to control AMR increases the risk of the spread of resistance to all other countries, especially those with low resistance rates [13,18]. Information on the occurrence/magnitude, sources/routes of transmission, and characteristics (phenotypic and genotypic) of clinically relevant and zoonotic organisms carrying resistance genes in the poultry sector in LMICs is important in devising strategies to curb the global spread of such organisms.

**Figure 1:**
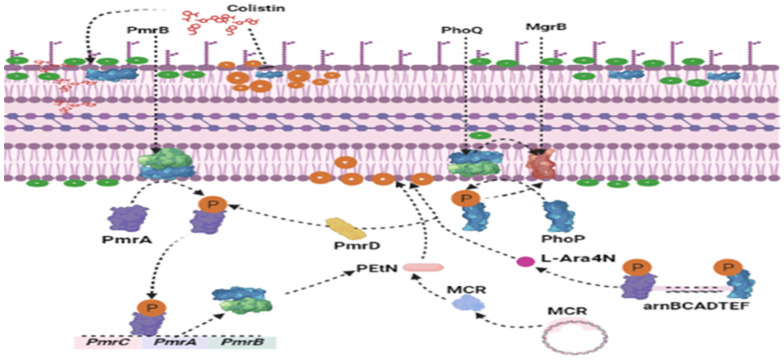
Plasmid-mediated colistin resistance mechanism in Gram-negative bacilli. Mobile colistin resistance (*mcr*) genes encode MCR proteins, which are cytoplasmic phosphoethanolamine (pEtN) transferase enzymes that modify lipid A by adding pEtN, resulting in a less negative net charge on the bacterial outer membrane, like the chromosomal pathways. Created with BioRender.com (accessed 29 May 2023).

COL is one of the last-line highest-priority critically important antibiotics (HP-CIAs) used to treat deadly infections caused by multidrug-resistant (MDR) and extensively drug-resistant (XDR) Gram-negative bacilli (GNB) in humans and animals. The administration of COL has been largely unregulated in LMICs for more than six decades [19]. Although the amount of global COL sulphate production decreased from 13,746 tons in 2016 to only 4292 tons in 2019, global poultry production accounts for over 49% of total COL sulphate usage, above the amount used in pig production, which accounts for 47.41% [19]. COL manufacturing, as well as the trade of pharmaceutical raw materials, finished pharmaceutical products, and feed additives or growth promoters for veterinary use, remained unchanged between 2017 and 2019 in some heavily populated LMICs [8]. The worst is that 6 LMICs among the 10 largest meat producers in the world do not report antimicrobial usage to the public [20]. Before 2015, the bacterial COL resistance mechanism was only understood to be chromosomally mediated and mutationally acquired, conferred by two-component systems such as *pmrAB*, *phoPQ*, *crrAB*, and *mgrB*, amongst others [21,22] (Figure 1). These chromosomal mechanisms are vertically transmitted, resulting in clonal dissemination, and thus, by their very nature, are self-limiting [23,24]. The emergence and spread of plasmid-mediated transmissible COL resistance genes, specifically mobile COL resistance (*mcr*) genes *mcr*-1 to *mcr*-10, first reported in late 2015, threaten the clinical efficacy of COL in veterinary and human medical practices [25]. Unfortunately, *mcr*-gene-bearing plasmids (especially conjugative plasmids) spread through inter- and intraspecies horizontal gene transfer (HGT) at lightning speed among Gram-negative bacilli (GNB) [23,26]. *mcr* genes have numerous variants and subvariants, which have been identified in isolates/samples from humans, animals, and environments across over 70 countries on all continents [27]. These genes encode MCR proteins, which are cytoplasmic transmembrane proteins/enzymes (phosphoethanolamine (pEtN) transferases) that confer resistance to COL through the attachment of a pEtN moiety to the lipid A of lipopolysaccharide found in the outer membrane of GNB [25,28] (Figure 1). This attachment of the pEtN moiety abolishes the negative charges on lipopolysaccharide (LPS) to which cationic COL/polymyxins have an affinity, thereby resulting in COL resistance [18] (Figure 1). The cocarriage of *mcr* with plasmid-encoded genes conferring resistance to fluoroquinolones and other last-resort HP-CIAs, such as genes encoding flavin monooxygenenases (TetX) and resistance-nodulation-drug efflux pumps (TmexCD-toprJ) (which mediate resistance to tigecycline), carbapenemases (which mediate resistance to carbapenems), extended-spectrum β-lactamases (ESBLs) and plasmidic ampicillinase C (pAmpC) (which mediate resistance to extended-spectrum cephalosporins), and glutathione S transferase (which mediate resistance to fosfomycin) is of further concern because such strains could become XDR or pandrug-resistant (PDR) in a single event of HGT, which could result in nearly untreatable infections, fatalities, and huge economic losses [26]. Unfortunately, plasmids carrying *mcr* genes with other last-resort antimicrobial determinants seem to be more stable, giving carriers a greater fitness advantage and enabling the easier and more rapid transfer of genes [29]. 

The poultry sector is a potential reservoir of COL-resistant organisms capable of causing community- and hospital-acquired intestinal/extraintestinal infections. The foodborne transfer of plasmid-mediated COL resistance to humans and the environment threatens global public health and socioeconomic development, which can only be effectively addressed through a One Health approach. Understanding the sources/routes of the transmission, incidence, and phenotypic and genotypic traits of *mcr* gene-containing bacteria (MGCB) from poultry birds/products and the environment in LMICs is crucial in devising effective strategies for controlling their spread to reduce the One Health risks involved. This review describes the epidemiology (causes of COL selection pressure, sources and routes of transmission, One Health impact, and prevalence), characteristics (genomic traits, genetic context of *mcr*, genetic and phenotypic resistance, virulence properties, and population structure), and control strategies for MGCB in the poultry sector in LMICs from 1970 to May 2023. 

## 2. Causes of Colistin Selection Pressure and Development of *mcr*-Gene-Carrying Organisms in Poultry Sector in LMICs

COL was largely abandoned for human use in the 1970s, soon after its production, due to its toxic kidney/nervous effects, alongside the discovery of safer and more effective antibiotics [30]. However, COL has continued to be used in most LMICs as topical applications in humans and as a growth enhancer [30]. It has also been administered for the prophylactic and chemotherapeutic treatment of intestinal bacterial infections in livestock, especially in poultry production [12,19]. In most LMICs, there are no veterinary antibiotic restrictions, and CIAs such as COL are readily available over the counter (OTC) and often marketed by non-professionals [11,31]. COL is often used without a veterinarian’s supervision and added at a sub-therapeutic dose to poultry feed for the prophylactic control of enteric infections and for promoting the growth of animals for higher socioeconomic returns [8,28]. In some LMICs, COL is often ignorantly administered in water in plastic drinking troughs, making COL bind to the plastics and thereby reducing the concentration to sub-therapeutic/subinhibitory levels [32,33]. Substandard COL freely circulates in some LMICs, and incorrect dosage regimens by farmers seem to be enhancing the problem of AMR in these countries [34]. These practices consequently result in COL selective pressure and the rapid acquisition of *mcr* genes by bacteria in poultry birds [25,28]. In most countries, other antimicrobial agents, especially tetracycline, florfenicol, and β-lactams, as well as bacitracin, are extensively used in poultry. This also contributes to prompting the acquisition of COL resistance determinants in poultry [35]. The poor absorption of COL in the gastrointestinal tract of birds and the consequent low bioavailability following oral administration equally triggers COL selective pressure [32,36]. 

## 3. Sources, Routes of Transmission, and One Health Impact of *mcr* Gene-Containing Organisms in the Poultry Sector in LMICs

### 3.1. Breeder Birds, Eggs, and Hatcheries

MGCB can vertically enter the poultry production sector even without antimicrobial use through day-old chicks (DOCs). In Brazil, *mcr*-9-positive *Enterobacter cloacae* was isolated from DOCs [37], while *mcr*-1-positive *E*. *coli* was isolated from DOCs in Vietnam [38] and Bangladesh [39,40,41]. Eggs infected by COL-resistant organisms, especially *Salmonella*, through vertical (in ovo) transmission in breeder flocks raised with COL or other antimicrobials are potential sources of infection of DOCs with MGCB [42]. In Bangladesh, *mcr*-1-, *mcr*-2-, and *mcr*-3-positive *E*. *coli* were isolated from breeder birds [39,40,41], while, in China, *mcr*-1-positive salmonellae was isolated from eggs [43,44]. MGCB can contaminate eggs through the on-farm contamination of eggshells by bird faeces. Genetically similar *mcr*-1-bearing salmonellae differing only by 20 to 21 single-nucleotide polymorphisms (SNPs) were isolated from eggs and the chickens that laid them [44], thus suggesting that chicken faeces were the likely contamination source of the organisms. Aside from the in ovo transmission and faecal contamination of eggs, contaminated hands of egg handlers and hatchery trays are also putative sources of egg contamination. Wang et al. [45] detected *mcr*-1 in Chinese hatcheries, and there was an overlap of sequence types (STs) showing genetic relatedness between *mcr*-1-positive *E*. *coli* from different poultry farms in China [46], Lebanon [47], Ecuador [47], and Nigeria, wherein DOCs were sourced from the same hatcheries [34]. In Paraguay, phylogenetically related *mcr*-5-positive *E*. *coli* was isolated from 28-day-old birds in all farms that sourced chickens from the same breeding/hatchery company [48], suggesting that organisms colonizing DOCs in hatcheries play a role in the development of the chicken microbiome with the colonizers and ARGs maintained.

### 3.2. Poultry Feeds 

Poultry feeds whose components might be contaminated pre- or post-harvest in the field, at the feed mill, or during the feeding of birds are potential sources of MGCB in poultry farms. In Malaysia [49] and Bangladesh [39,40,41], *mcr*-1-positive *E*. *coli* was isolated from poultry feeds, and in Lebanon, there was genetic relatedness indicated by the overlap of STs (ST101 and ST746) between *mcr*-1-bearing *E*. *coli* from chickens and that from poultry feeds consumed by the chickens [47], suggesting that the birds possibly acquired the organisms from the feed. Poultry litter, feed/feedstuff handlers, and vectors (such as synanthropic insects and rodents) are potential sources of MGCB in poultry feed. Fish meal, often used as a source of protein in poultry feed, is also a potential source of *mcr* gene-bearing organisms (such as *Aeromonas*, which is a common inhabitant of the aquatic environment) in the poultry sector [19]. 

### 3.3. Birds’ Drinking Water

Birds’ drinking water contaminated by humans and animals in-farm or at the source could result in the spread of MGCB in the poultry population. In China [49,50,51,52], Bangladesh [39,53], Malaysia [54], and Indonesia [55], *mcr*-1-positive *E*. *coli* was isolated from poultry farm water and the farm water sources (ponds and deep water), while *mcr* genes were detected in birds’ drinking water samples in Vietnam [56]. Colonization of the oropharyngeal cavities of birds by MGCB is a risk factor for the contamination of birds’ drinking water and feed. Zhang et al. [57] detected *mcr*-1, *mcr*-2, and *mcr*-3 in oropharyngeal swabs from birds (chickens, ducks, geese, and pigeons) in China, suggesting that the birds possibly acquired MGCB from litter, feed, and/or drinking water. 

### 3.4. Contaminated Farm Equipment and Environment

Cyclical contamination of poultry farms is also possible if MGCB persists in the farm environment. In Bangladesh, *mcr*-1-positive *E*. *coli* was recovered from the floors of pens and the ventilators in poultry farms [53], suggesting that MGCB can survive on poultry farms, even after culling, and that contaminated ventilators are potential sources for the airborne transmission of MGCB in livestock farms. The *mcr*-1 gene was detected in all samples (farm environmental samples, faeces, solid poultry manure, and soil) collected from the same site from 2017 to 2019 in China [58], suggesting that *mcr* gene-bearing organisms can persist in the poultry farm environment for a considerably long time.

### 3.5. Vectors

#### 3.5.1. Mammalian Vectors

Carrion-eating animals (such as carnivores—dogs and cats, and rodents) and humans can be infected by MGCB when they consume carcasses of dead birds since these organisms have been isolated from the internal organs of birds, including sick and dead birds in China [59,60,61,62,63,64], Bangladesh [40,41,53,65,66], Pakistan [67], Malaysia [54,68], Nepal [69,70,71], Tunisia [72,73,74,75], Egypt [2,76,77,78,79,80], and Morocco [81]. Accordingly, *mcr*-4-positive salmonellae and *mcr*-1-positive *E*. *coli* were isolated from rats captured in poultry farms in South Africa [82] and Thailand [83], respectively. The rectal carriage of *mcr*-1-positive *E*. *coli* was observed in dogs found in poultry farm environments in Vietnam [84] and China [85,86], while Zhou et al. [87] isolated *mcr*-3-positive *E*. *coli* from dogs in contact with livestock in Laos. The use of poultry meat and/or by-products (such as internal organs of food animals from slaughterhouses) contaminated with COL-resistant organisms in feeding or formulating foods for carnivores (pets such as dogs and cats) and fish is a potential route for transferring MGCB from the poultry sector to these animals, which closely interact with humans.

#### 3.5.2. Flies 

Synanthropic flies that feed/breed in poultry litter/manure can be colonized on the body surface and in the gut by MGCB and subsequently transport the organisms from the poultry environment to other places. In Pakistan, flies captured around poultry farms harboured *mcr*-1-positive *E*. *coli* [8]. In Bangladesh [88] and Laos PDR [87], *mcr*-3-bearing *E*. *coli* were isolated from flies captured in poultry farms. In China, flies captured in poultry slaughterhouses and farms harboured *mcr*-1-positive *Rauoltella* [85,86], while those captured in proximity to poultry farms harboured *mcr*-1-positive *E*. *coli* that was genetically similar to that from the birds [89]. Thus, flies are potential carriers/sources of genetically related (overlapping STs) bacterial pathogens in different farms within a locality. These contaminated flies could mechanically transport MGCB to humans when they land on wounds, foods, and fomites [23].

#### 3.5.3. Migratory/Free-Range Wild, Urban, and Aquatic Birds 

Wild migratory birds can acquire MGCB through contact with poultry manure/litter/sewage in farms, slaughterhouses, or crop farms fertilized with untreated/insufficiently treated poultry manure/slaughterhouse sewage. Umair et al. [8] isolated *mcr*-1-positive *E*. *coli* from the droppings of wild birds (crows and kites) scavenging on poultry wastes/human sewage in Pakistan, implying that poultry manure is a source of MGCB colonization of wild birds. In China, *mcr*-1-positive *E*. *coli* was isolated from pigeons [90,91,92], indicating that migratory urban birds are potential sources of human and environmental contamination with MGCB through the faecal contamination of public places, such as parks, markets, and surface waters. This is of concern in countries such as China, where antimicrobials are heavily used in the pigeon industry, a major source of animal protein [92]. Free-range birds are also a potential source for disseminating MGCB in the environment. In Bangladesh [93] and Pakistan [8], native and backyard free-range chickens that lived in proximity to humans harboured *mcr*-1-positive *E*. *coli*. This suggests that these birds possibly picked up the organisms from an anthropogenically/agriculturally impacted environment and are a putative source of environmental contamination with MGCB. In China, *mcr*-1-positive *Enterobacterales* and *mcr*-3-positive *Aeromonas* have been frequently recovered from waterfowl such as ducks and geese [63,89,94,95,96,97,98,99,100]. As aquatic birds, these waterfowl possibly picked up MGCB from anthropogenically/agriculturally impacted waters. Nevertheless, huge amounts of antimicrobials are used in the Chinese waterfowl industry [101], such that MGCB could also evolve in waterfowl, thereby serving as a potential reservoir/source of environmental/surface water contamination. The exchange of MGCB between water sources and humans occurs when individuals (such as swimmers and persons/animals that drink these waters) encounter contaminated water during laundering, cooking, drinking, bathing, food processing, and outdoor recreational activities often practiced in LMICs [23].

### 3.6. Poultry Farm Litter/Manure/Slaughterhouse Sewage 

Poultry litter/manure in farms constitutes a potential source of the colonization of birds by MGCB. A study in Lebanon reported an overlap of STs of *mcr*-1-positive *E*. *coli* between isolates from chickens and chicken farm litter [47], suggesting that either the birds picked up the organisms from the litter or that they emanated from the animals. Poultry litter/manure/sewage used as organic fertilizer on crop farms is also a putative source for disseminating MGCB into the soil, botanical, and wildlife environments. Poultry slaughterhouse sewage contained *mcr*-1-positive *E*. *coli* in China [49,50,51,52] and Vietnam [84]. In Bangladesh, farm poultry litter contained *mcr*-gene-positive organisms [39,40,41], while Das et al. [40] isolated *mcr*-1-positive *E*. *coli* from the soil on a Bangladeshi poultry farm. In Brazil, *mcr*-1 was detected in soil that received non-composted poultry litter as organic fertilizer and in areas without livestock manure [102], suggesting that, through rainfall run-off, MGCB can filter into non-agriculturally impacted areas. Crop farmers, especially those without personal protective equipment (PPE), who encounter soil fertilized with poultry manure are at risk of being infected with MGCB [30,103]. Rainfall can unearth microorganisms from the soil/animal manure, resulting in the contamination/colonization of vegetables and crops, thereby reincorporating MGCB into the food chain [23,30]. Through rainwater run-off, MGCB from poultry manure disposed of on land can filter into environmental waters and subsequently spread by water currents to other places. In Laos, Zhou et al. [87] isolated extensively diversified (genetically unrelated) *mcr*-3-bearing *E*. *coli* from surface water within a 10 km radius of poultry farms. Animals and other aquatic organisms (free-range and feral mammals, birds, amphibians, and molluscs) depending on these contaminated waters for sustenance could become infected by MGCB and subsequently transport the organisms to other places [13,23]. 

### 3.7. Integrated Poultry–Fish Farms

Integrated fish farms where fish are fed livestock (poultry) manure, which often contain un-metabolized antimicrobials (including COL, which is poorly absorbed in the chicken intestine), are also potential sources for the dissemination of MGCB and COL selective pressure from poultry into aquatic–human–environmental ecosystems [19,38]. In a Chinese study, ducks in integrated duck–fish farms harboured *mcr*-1-positive *E*. *coli* that was closely related to *E*. *coli* strains from humans in the fishery farm region [50]. Though the fish in integrated farms are not given any antimicrobial treatments, the poultry birds are often reared with various antimicrobials, including COL, which is stable and persists for a long time in the water, thereby serving as a source of selective pressure and antimicrobial-resistant organisms/genes in aquafarms [23,104]. The Indochinese peninsula is of particular concern because countries (such as Vietnam and others) in this region have a lot of integrated aquaculture systems, where fish are often fed with the faeces of animals reared with extensive antibiotic usage [15,19]. Moreover, using poultry manure as organic fertilizer for increasing phytoplankton growth in aquaculture (a common economical method for feeding fish in LMICs) is also a means for transferring MGCB from poultry to the aquatic environment. In addition, feeding farmed fish with insect larvae (maggots) raised in poultry manure is also a potential route for disseminating MGCB from the poultry sector to the environment [30].

### 3.8. Poultry Meat

The carriage of MGCB by birds could result in the cross-contamination of poultry meat and associated products by these organisms during slaughter, processing, packaging, and selling. Studies have reported the presence of *mcr*-gene-harbouring organisms in poultry meats obtained from butchers/slaughterhouses/retail markets/supermarkets in China [57,105,106,107,108,109] (Table S1), India [110], Bangladesh [110], Nepal [69,70,71], Türkiye [111,112], Pakistan [8], Vietnam [84,113,114,115,116,117,118,119], Indonesia [55], Malaysia [54,120], Iraq [121], Laos [87,122], Tunisia [74], Algeria [123], Egypt [77,78,79], Brazil [124,125], the Dominican Republic [126], and Russia [127]. Worst of all, the storage temperature does not halt the exchange of *mcr* genes among meat-contaminating organisms since poultry meats stored at 4 °C, 25 °C, and 37 °C were conducive to the rapid exchange of *mcr*-1 between *Salmonella* and *E*. *coli* [5]. Handlers (such as butchers and food preparers, especially those with poor hygiene and without PPE) of raw poultry meat and consumers of raw and half-cooked eggs/and poultry meat are at greater risk of being infected with MGCB. This is because *mcr*-1-positive *S*. Typhimurium was isolated from the internal contents of eggs in China [44], *mcr*-bearing organisms were recovered from street foods in Bangladesh [40,66,128], and ST11 *mcr*-1-positive *Salmonella* Enteritidis that contaminated poultry meat was associated with an outbreak of human salmonellosis in Russia [127]. 

### 3.9. Humans in Contact with Poultry Birds, Products, and Environment

#### Poultry Farm/Slaughterhouse Workers and Their Workwear

Since a diversity of poultry birds (chickens, geese, turkeys, quail, pigeons, and ducks, amongst others) are established reservoirs of MGCB (Table 1, Table 2, Table 3 and Table 4), individuals such as poultry farmworkers, poultry slaughterhouse/meat-processing facility workers, and veterinarians who come into direct contact with poultry birds/wastes constitute potential vehicles for transporting MGCB from the poultry environment to the public/communities and vice versa. In China, *mcr*-8-positive *Klebsiella pneumoniae* was isolated from poultry birds and their caretakers, suggesting the possible exchange of the organisms between humans and birds [129]. Another Chinese group isolated *mcr*-1-positive *E*. *coli* from Chinese poultry farmworkers [45]. Investigators in Vietnam [84,117], Thailand [83,130], Lebanon [131], and Egypt [77] recovered *mcr*-1-positive *E*. *coli* from poultry farm workers. Remarkably, the Vietnamese isolates from farmers and birds had zero to one single-nucleotide polymorphism (SNP) between them and belonged to the same clone (clonal complex (CC) 10), showing that they were related [117]. In addition, isolates from the Thai poultry farmers and the birds that they cared for belonged to the same ST2973 clone, indicating they were related and possibly exchanged between humans and animals [83,130]. Furthermore, in China, *mcr*-1-positive ST34 *Salmonella* was recovered from the poultry sector and humans with diarrhoea [43], while, in Lebanon, *mcr*-1-positive *E*. *coli* of the same clone (ST1011) was recovered from poultry farmworkers and hospitalized individuals [131,132]. These findings suggest that these *mcr*-1-positive clones have successfully disseminated in poultry and human settings in these countries. Notably, no IPC measures were taken in the Lebanese farms from where *mcr*-1-positive organisms were recovered from poultry farmworkers [131], and the footbaths of poultry farmworkers in Bangladesh yielded *mcr*-1-postive *E*. *coli* [53]. Thus, working on poultry farms without taking IPC measures, such as wearing gloves, face masks, and boots and hand washing, is a putative risk for acquiring MGCB, especially following the handling of contaminated litter and feed or colonized/infected birds. It also implies that poultry farmworkers’ workwear is a vehicle for transporting MGCB from poultry farms to other places.

### 3.10. Poultry Bird Vendors 

Since poultry vendors come into direct contact with birds, they are potentially a major source of the local transmission of MGCB originating from the poultry environment to human communities and vice versa. Chicken vendors in China [152], Peru [208], and Nepal [71] harboured *mcr*-1-positive *E*. *coli*. This suggests that these vendors possibly acquired the organism from the birds, probably due to poor hand hygiene after bird handling.

### 3.11. Persons in Proximity to Poultry Birds/Environments

Individuals who have indirect contact, such as those in proximity to poultry birds/environments, are potentially at risk of being infected by MGCB. Some persons living near poultry farms/slaughterhouses in China harboured *mcr*-10-positive *Enterobacter kobei*, possibly originating from the poultry environment [139,167]. In Ecuador, a faecal sample from a boy who never received COL therapy yielded *mcr*-1-positive ST609 *E*. *coli* (a clone associated with animals), possibly acquired from his backyard soil contaminated with chicken faeces containing *mcr*-1-positive ST3941 *E*. *coli* (a clone associated with human infection) [205,221], thus suggesting the lateral dissemination of *mcr*-1 to unrelated *E*. *coli* strains in the same environment. In the same country, *mcr*-1-positive *E*. *coli* and *K*. *pneumoniae* were isolated from backyard chickens and their caretakers [206], further indicating the zoonotic transmission of the organisms.

### 3.12. Poultry Meat/Egg Handlers 

Meat handlers (such as butchers, slaughterhouse workers, potential buyers, and food preparers) constitute a major source of the cross-contamination of poultry meat by MGCB. In Bangladesh [181] and Tunisia [74], *mcr*-1-positive *E*. *coli* belonging to ST162 and ST117 clones, which are mostly associated with humans, were isolated from poultry meats and their handlers, respectively, suggesting the cross-contamination of meat from humans and vice versa. Other putative sources of MGCB in meat include unhygienic slaughterhouse environments, water used for carcass washing, and equipment used for meat processing, carriage, and display (such as knives, wheelbarrows, display table surfaces, etc.). Furthermore, synanthropic flies could potentially introduce MGCB acquired from other sources when they land on meat (in slaughterhouses, retail points, and open-air markets), and they could also transport organisms from meat to human and environmental domains. In Laos, Zhou et al. [87] isolated genetically related *E. coli* strains that coexpressed *mcr*-1 and *mcr*-3 from humans, flies, and chicken meat, suggesting the cross-sectorial dissemination of these organisms.

### 3.13. Trade of Poultry Birds and Products

The trade of poultry birds is a potential route for the international dissemination of MGCB. Chickens imported as day-old chicks from Croatia to Serbia harboured *mcr*-1-positive *E*. *coli* [220]. Grami et al. [73] isolated *mcr*-1-positive *E*. *coli* from Tunisian poultry farms that imported chickens from France or derived their flocks from French chicks. However, *mcr*-1 could also have been acquired during the birds’ lives in these countries. The trade of chicken meat/eggs also constitutes a potential route for the intercontinental transmission of MGCB. Chicken meat exported from Brazil to Asia, Europe, and other South American countries was contaminated by *mcr*-1- and *mcr*-9-positive *Enterobacterales* [37,222,223]. Live poultry bird trade could facilitate the local spread of MGCB at human–animal–environment interfaces. Metagenomics in China detected *mcr*-1, *mcr*-2, *mcr*-3, *mcr*-4, and *mcr*-5 in gut samples from poultry birds, live poultry bird handlers, and the live poultry market environment [57,145,152,224], indicating that individuals (such as sellers, buyers, and animal/environmental health workers/inspectors) who visit live poultry bird markets could acquire MGCB from persons in the markets, fomites, birds, and market environments.

### 3.14. Travel

Through international travel (even short-distance travel), *mcr*-gene-harbouring organisms originating from the poultry sector can disseminate globally from the point of emergence. Some *mcr*-1-positive ESBL-producing *E*. *coli* strains from the Chinese poultry sector were genetically related to Vietnamese human *mcr*-1-positive ESBL-producing *E*. *coli* strains, differing only by 0–150 SNPs [225,226], suggesting that the strains were transported from Southeast Asian countries to China, possibly through the meat trade and/or international travel. Countries that are attractions for international sports/social and religious events constitute hotspots for the colonization of tourists by MGCB circulating in the poultry meat production and supply industry [30]. 

**Figure 2:**
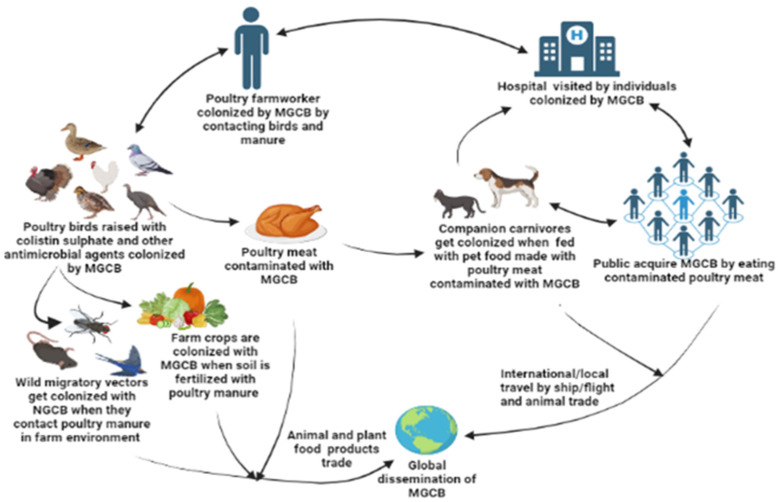
Possible sources and routes of transmission of *mcr*-gene-containing bacteria (MGCB) originating from the poultry sector to the One Health triad (human–animal–environment) in low- and middle-income countries. Created with BioRender.com accessed on 22 May 2023.

Indeed, MGCB emerging from the poultry sector can be disseminated through various ecological niches (human–animal–environment–aquaculture interfaces) (Figure 2). The rapid dissemination of the *mcr* gene among bacteria colonizing the gut of infected individuals (humans and animals) could result in the compromise of antimicrobial therapies that they receive, potentially resulting in public health threats and economic losses due to diseases that are difficult to nearly impossible to treat. These individuals could subsequently spread these organisms into the environment through unhygienic practices, such as open defaecation, improper agricultural waste disposal, and the nonapplication of basic IPC measures such as hand hygiene and environmental sanitation, as is common in LMICs [30]. Thus, MGCB in the poultry sector in LMICs have serious One Health ramifications.

## 4. Regional and Country-Wise Prevalence and Characteristics of *mcr* Gene-Containing Organisms in the Poultry Sector in LMICs 

### 4.1. Asia

The successive outbreaks of African swine fever and the COVID-9 pandemic in Asia led to decreased pork production with a rapid increase in poultry production, up to 8.79% higher than in 2018, on the continent [5,10]. In addition, the burgeoning population and emerging economies in Asia (which are predominantly LMICs) could potentially result in an increased demand for animal protein and the further abuse of critically important antibiotics, including COL, by 2030 if no action is taken [16,227,228]. This could have a catastrophic global impact due to rapidly increasing travel to and from Asia [228]. Therefore, information on plasmid-mediated colistin resistance in the poultry sector in Asian countries is crucial for devising a holistic approach to tackling this global problem.

One hundred and thirty publications reported on *mcr* genes in poultry meat supply chains in 15 (37.5%) of the 40 LMICs (among 50 countries) in Asia [10] (Table 1). Eight of the studies investigated the presence of *mcr* genes directly in the samples [56,57,58,145,171,229,230,231]. These studies investigated *mcr* genes in a total of 22,740 isolates and reported *mcr*-1 gene variants in 4024 isolates (3832 *Escherichia coli*, 62 *Klebsiella*, 45 *Salmonella*, 33 *Escherichia fergusonii*, 26 *Acinetobacter baumanii*, 18 *Proteus*, 3 *Enterobacter cloacae*, 2 *Citrobacter sakazakii*, and 1 each for *Providencia alcalifaciens*, *Rauoltoella planticola*, and *Shigella*), *mcr*-2 in 72 isolates (67 *E*. *coli*, 2 *Proteus*, and 1 each for *K*. *pneumoniae*, *Salmonella*, and *Enterobacter*), *mcr*-3-gene-type variants in 19 isolates (13 *E*. *coli*, 5 *Aeromonas*, and 1 *Proteus mirabilis*), *mcr*-7 in 3 *K*. *pneumoniae*, *mcr*-8 in 30 isolates (28 *K*. *pneumoniae* and 2 *R*. *ornithonilytica*), *mcr*-9 in 133 salmonellae, and *mcr*-10 in 5 isolates (2 each for *K*. *pneumoniae* and *Enterobacter* and 1 *E*. *coli*), respectively.

#### 4.1.1. Eastern Asia 

##### China

China is a key producer of poultry birds and poultry products, and the majority (54.6%, 71/130) of studies on *mcr* genes in Asian LMICs’ poultry sectors originated from this country (Table 1) (Table S1). China accounts for ~80% of the production market of global COL sulphate and is by far the largest consumer (45% of global use in 2017 and predicted to remain the topmost consumer at 43% by 2030 [16]) of antimicrobials, including COL, having consumed (for prophylactic control/growth promotion in the poultry sector) > 90% of 17.5 million tons of the COL that it produced in 2014 [16,19,36]. This exerted selective pressure, leading to MGCB being dispersed into diverse ecological niches in China [23,225]. So, the ban on COL use for nontherapeutic purposes, which was gazetted in November 2016 but took effect in April 2017, is expected to withdraw that amount of COL from the Chinese livestock industry, thereby reducing the magnitude of MGCB in the sector [232,233,234]. Therefore, information about the epidemiology and traits of MGCB isolated from the Chinese poultry sector before and during the ban is important for understanding the efficacy of the ban and enhancing existing strategies to control the spread of MGCB. A total of 63 publications documented the presence of *mcr* genes in isolates from the Chinese poultry sector (Table 1). Of these studies, 37 (58.7%) assessed *mcr* genes in isolates recovered from 2016 and earlier (i.e., before the ban), while 26 (41.3%) probed genes in isolates recovered from 2017 onwards (i.e., during the enforcement of the ban) (Table S1), suggesting that there are more publications on the incidence of *mcr* in Chinese poultry before than during the ban enforcement. However, some studies assayed *mcr* in isolates obtained during a period that spanned before and during the ban [63,85,133,135,157,162]. 

A diversity of bacteria isolated at varied rates have been incriminated as traffickers of *mcr* genes in the Chinese poultry sector. A total of 2422 (18.6%) strains carrying *mcr* genes (*mcr*-1—2346; *mcr*-2—66; and *mcr*-3 and *mcr*-10—1 each) were detected among 12,993 *E*. *coli* isolated between 1970 and May 2023 from poultry birds (chickens, ducks, pigeons, geese, and quail), meats, and the environment (Table 1; Table S1). Among 213 *E*. *fergusonii* isolated between 2019 and 2021, 33 (15.5%) *mcr*-1-positive strains were detected [100,137,163]. Of 345 klebsiellae isolates, 43 (12.5%) *K*. *pneumoniae* carrying *mcr* genes (*mcr*-8—23; *mcr*-1—p; *mcr*-7—3; and *mcr*-10—2) were detected [49,50,51,52,129]. Among 3005 salmonellae isolated between 2007 and 2021, 148 (4.9%) strains carrying *mcr* genes (*mcr*-1—24; *mcr*-9—131) were detected [43,44,94,97,98,99,100,165], while a total of 5 (8.6%) *mcr*-3-bearing strains (2 *A*. *veronii* and 1 each for *A*. *media*, *A*. *salmonicida*, *A*. *allosacharophila*, and *A*. *caviae*) were detected among 58 Aeromonads [168,169,170,235]. These findings indicate that various *mcr* gene types (*mcr*-1, *mcr*-2, *mcr*-3, *mcr*-7, *mcr*-8, *mcr*-9, and *mcr*-10), predominated by *mcr*-1, are widely spread in the Chinese poultry sector. The findings also show that *E*. *coli* is the dominant *mcr* gene trafficker in China. In addition, the findings reveal that *E*. *fergusonii* is spreading *mcr*-1 and has been an underrated repository of *mcr* genes and that *Klebsiella* traffics the highest diversity of *mcr* gene types and ranks second only to *Escherichia* in *mcr* gene transmission propensity. Furthermore, the findings suggest that *Salmonella* is a major reservoir of *mcr*-9 and that it disseminates various *mcr* genes but at a lower rate than *Escherichia* and *Klebsiella*, while *Aeromonas* is disseminating *mcr*-3, albeit at a low rate, in the Chinese poultry sector. 

Other traffickers of *mcr* genes isolated at a low rate from the Chinese poultry sector include *Enterobacter* (three strains—one *mcr*-1-positive *E*. *cloacae* and one each of *mcr*-10-positive *E*. *roggenkampii* and *E*. *kobei*) [85,139,167], *Rauoltella* (three strains—one *mcr*-1-positive *R*. *planticola* and two *mcr*-8-positive *R*. *ornithinolytica*) [85,86], *Cronobacter sakazakii* (two *mcr*-1-carrying strains), *Proteus mirabilis* (one *mcr*-3.10-positive strain) [158,169], *Citrobacter braakii* (one *mcr*-1-positive strain) [134] and *Providencia alcalifaciens* (one *mcr*-1-positive strain) [85]. Thus, uncommon organisms such as *Rauoltella* and those in the *Proteae* group (*Proteus* and *Providencia*), thought to be naturally resistant to COL, are potential reservoirs of *mcr* genes. 

Although uncommon, a diversity of *Enterobacterales* from the Chinese poultry sector coexpressed different *mcr* gene types, albeit at a low prevalence. *mcr*-1 and *mcr*-2 were simultaneously harboured by 1.32% (32/2422) of *mcr*-positive *E*. *coli* isolates [57], while 2 (4.7%) of the 43 *mcr*-positive *K*. *pneumoniae* isolates coexpressed *mcr*-8 and *mcr*-10 [167]. It is speculated that selective pressure by different antimicrobials could facilitate the carriage of diverse *mcr* genes on a plasmid [18,236]. 

Information about the incidence of MGCB before and during the ban on nontherapeutic COL use is crucial in establishing the efficacy of the ban as an intervention strategy for curbing the problem of AMR. Since the ban was gazetted in November 2016 but its enforcement began in April 2017 in China [225,233], and the sporadic development of AMR resistance can occur following exposure to an antimicrobial agent [237], the assessment of the impact of the ban on the development of MGCB should therefore begin from In addition, since *E*. *coli* is established to have the highest propensity to acquire *mcr*-1, which is the most trafficked *mcr* gene type, it is ideal/logical to use the isolation prevalence of *mcr*-1-positive *E*. *coli* as the criterion for determining the impact of the ban on *mcr* gene-containing bacterial development. Logically, isolates recovered during a period that spanned before and during the ban enforcement without a clear indication of their date of isolation [58,63,135,157,160,162] (Table S1) are not good candidates for assessing the impact of the ban; hence, they were not included in this review. From publicly available data, before the ban enforcement (i.e., 2016 and earlier), 1262 (17.8%) of 6703 *E*. *coli* isolates from the Chinese poultry sector were *mcr*-1 gene carriers (Table S1). During the ban (from 2017 to May 2023), 335 (12.4%) of 2689 *E*. *coli* isolates harboured the *mcr*-1 gene [43,46,61,89,92,94,159,161,164] (Table S1). Thus, *mcr*-1-positive *E*. *coli* prevalence during the ban (12.4%) is less than that during pre-ban enforcement (17.8%). This suggests that the prevalence of *mcr*-1-positive *E*. *coli* in the Chinese poultry sector is decreasing. This is consistent with the results of studies on the impact of the COL ban on the prevalence of *mcr*-1-positive *E*. *coli* in the Chinese poultry sector [136,160,161]. Specifically, Wang et al. [160] reported that the *mcr*-1-positive *E*. *coli* prevalence in poultry farms decreased from 18.7% in 2015–2016 to 5.0% in 2017–2018; Zhang et al. [161] observed that *mcr*-1-positive *E*. *coli* was quite low in 2014 (2.3%) and 2015 (1.7%), increased to a peak in 2016 (12.6%) and 2017 (11.4%), and then decreased significantly in 2018 (1.7%) and 2019 (0.9%) after the ban was enforced; Yang et al. [136] analysed *E*. *coli* isolated between 1970 and 2019, including 567 chicken isolates, and reported that COL resistance and *mcr*-1 prevalence increased rapidly during the 2000s but decreased from 44.27% in 2010–2016 to 23.08% in 2017–2019 after the ban was enforced. The reasons suggested for the post-ban reduction in the prevalence of *mcr*-1-positive *E*. *coli* include *mcr*-mediated target modification of the lipid A moiety of LPS being the sole transferable mechanism of COL resistance in *E. coli*, *mcr*-1 expression imposing a considerable fitness cost for *E. coli* carrying *mcr*-bearing plasmids, the ban on using COL as a growth promoter significantly reducing the selection for *mcr*-1, and/or *mcr* genes being seldomly found together with other ARGs on the same MGEs reducing the likelihood of coselection by other antimicrobial agents [136]. Additionally, it was suggested that MCR-1 affects lipid homeostasis in bacterial cells, causing lipid remodelling, resulting in an outer membrane permeability defect, thus compromising the viability of Gram-negative bacteria [238]. Therefore, COL selective pressure in China was likely reduced because the ban on the use of COL as a growth promoter in China led to the reduced inclusion of COL as an additive in livestock feed in China [160]. This in turn favourably resulted in a marked reduction in COL production in China from 27,170 tonnes in 2015 to 2497 tonnes in 2018; hence, the sales of COL sulphate premixes declined from USD 71.5 million in 2015 to USD 8.0 million in The cumulative effect of the ban was a decline in global COL sulphate production from 13,746 tons in 2016 to 4292 tons in Furthermore, COL selective pressure in Chinese environments was indirectly reduced as the mean COL residue concentration in livestock manure was significantly reduced from 191.1 μg/kg in 2017 to 7.5 μg/kg in 2018, while the median relative abundance of *mcr-1* per 16S RNA was equally significantly reduced from 0.0009 in 2017 to 0.0002 in 2018 [160]. The carriage of *mcr*-1-positive *E*. *coli* in healthy and sick/hospitalized persons in China also appeared to be reduced with decreasing COL use [46,160,239]. Thus, enforcing a ban on nontherapeutic COL use in livestock is an effective strategy to curb the development and spread of MGCB [160]. 

Various plasmids, especially broad-host incompatibilities, formed the backbone of *mcr* in the isolates (Table 1). The IncI, IncH, IncF IncX4, IncP, Incp011, IncY, and IncN plasmids, among others, predominated in *E*. *coli* isolates. In *E*. *fergusonii* isolates, IncI and IncHI2 were the only backbones for *mcr*-1 [137,138,163]. The *Enterobacter* isolates contained only *mcr*-1-IncX4 and *mcr*-10-IncFII [85], while IncI2, IncX4, and IncHI2 (with only the IncHI2A plasmid associated with *mcr*-9) were associated with *mcr*-1 in *Salmonella* isolates [43]. In *Rauoltella* isolates, IncI and IncFII plasmids were the backbones of *mcr*-1 and *mcr*-8, respectively [85,86]. Thus, the diversity of plasmids is rapidly spreading *mcr* genes among *Enterobacterales* in China. These plasmids were mobilized with or without the use of COL since they remained stable after 14 days of passaging with and without COL [25]. However, *mcr*-1 was also transmitted among the isolates by prophage-mediated horizontal transmission (transduction) since *mcr*-1 was borne on a phage-like IncY plasmid in an *E*. *coli* strain [155]. Notably, *mcr*-9-IncHI2A existed in a diversity of *Salmonella* serotypes predominated by *S*. Thompson [98,99], thereby contrasting with high-income countries’ poultry sectors, wherein *mcr*-9 was predominantly associated with *S*. Saintpaul and *S*. Java [18], while *mcr*-10 with multiple resistance genes, including *tmexCD1-toprJ1*, was harboured by a hybrid IncFIB-HI1B plasmid in *E*. *coli* isolates [167]. Thus, suggesting that plasmid homologous recombination could occur in the recipient organisms following the acquisition of *mcr* with or without other resistance genes. 

Aside from plasmids, other mobile genetic elements (MGEs), such as insertion sequences (ISs) and transposons, were associated with *mcr* in the isolates. The composite transposon IS*Apl1* of the IS*30* insertion sequence family was the dominant transposon that facilitated the acquisition of *mcr*-1 by the isolates. The position of IS*Apl1* relative to *mcr*-1 in the plasmids varied among the isolates. IS*Apl1* flanked *mcr*-1 upstream in various plasmids, such as IncI2, IncHI2, IncFII, IncP, and so on, in *Escherichia* [25,50,59,141,143,144,157,158], in the IncI2 plasmid in *E. fergusonnii* [163], and in IncI2, IncHI2, and IncFIB–HI2 (hybrid plasmid) plasmids in *Salmonella* isolates [5]. However, IS*Apl1* only flanked *mcr*-1 downstream in IncHI2 and IncI plasmids in *E*. *coli* isolates [106]. These findings suggest that the IS*Apl1* position has diverged over time in a diversity of *mcr-*bearing plasmids in *Enterobacterales*.

The mobile transposon Tn*6330* (IS*Apl1*-*mcr*-1-*pap2*-IS*Apl1* or IS*Apl1*-*mcr*-1-*orf*-IS*Apl1*), which contains two copies of IS*Apl1* bracketing *mcr*-1 upstream and downstream, occurred in IncI and IncHI2 plasmids in *E*. *coli* isolates, even in isolates that coexpressed ESBL, pAmpC, and carbapenemase [25,106,143,148], and in the IncHI2 plasmid in *Salmonella* Tyhimurium [43]. Thus, IncHI2 and IncI plasmids could have more copies of ISs (especially IS*Apl1*) in multidrug-resistant isolates, thereby making these plasmid types more dangerous in *mcr* gene dissemination [240]. Tn*6330* often has a conserved segment, being the ancestral unit for *mcr*-1 transmission; interestingly, the variation in the conserved segment revealed that the divergence of the ancestral *mcr*-1 in China began in at least 1982, which also coincided with the time that COL use as a growth enhancer in poultry began in mainland China [240]. The presence of one or two copies of IS*Apl1* represents the recent acquisition of the *mcr* gene [241]. It has been reported that *mcr*-1 sequences with two copies of IS*Apl1* are less frequently found than sequences with only a single copy of IS*Apl1* [209]. 

Tn*6330* also translocated *mcr*-1 into the chromosome of some *E*. *coli* isolates [90,91], similar to *mcr*-1-positive *E*. *coli* isolates from humans and the environment in China [242]. Chromosomally integrated *mcr*-1 and *mcr*-3 also existed in isolates of *Salmonella* [96] and *Aeromonas* [170], respectively, showing that a diversity of *mcr* gene types could stabilize, persist, and vertically/clonally disseminate among different genera of *Enterobacterales* and non-fermenting Gram-negative bacilli. However, *mcr*-1 was located on a chromosomal integrative conjugative element (ICE), ICESk1, in *Salmonella* Kentucky from chicken meat [243], and IS*6* flanked *mcr*-1 downstream in the chromosome of *S*. Indiana from chicken meat [153], thus suggesting that ICEs (which are modular MGEs integrated into a host genome and are passively propagated during chromosomal replication and cell division) and ISs could be involved in the integration and mobilization of the chromosomally encoded *mcr* gene. Aside from chromosomal integration, polymorphic plasmids, which are chromosomally integrated plasmids [244], could also cause the persistence of *mcr* genes even after the withdrawal of COL. Additionally, the coevolution of host–plasmid pairs resulting from compensatory mutations in the host chromosome or the plasmid could diminish the fitness cost (following the acquisition of the plasmid), thereby allowing *mcr* plasmids to better persist [99]. 

Nonetheless, IS*Apl1* was lost entirely in *mcr*-1 plasmids in isolates of *E*. *coli* [106,154], *Rauoltella planticola* [85], and *E*. *fergusonii* [163], wherein it was replaced upstream of *mcr*-1 by *pap2*—a gene speculated to be compensating for phospholipid metabolism functions altered during LPS modification by COL resistance determinants [245]—and downstream by the *nikB* gene. This implies that the loss of IS*Apl1* does not hinder the transmission of the *mcr* gene. IS*Apl1* loss is suggested to imply the stabilization of the *mcr* gene and could also correspond to the adaptation of the gene to a new host [241]. It has been noted that most *mcr*-1-positive isolates do not present the IS*Apl1* sequence [209]. Yassin et al. [95] showed that *mcr*-1 with/without IS*Apl1* in avian isolates could be maintained at an unchanged level through 60 generations, thus reiterating that it may take a long time, if not practically impossible, to eliminate the *mcr* gene from the environment, even in the absence of COL use [23]. Remarkably, IS*Apl1* was entirely lost, which originally existed upstream of *mcr*-1 following a conjugative transfer of *mcr*-1-IncI2 to recipient cells in chicken meat at 25 °C [5]. Thus, this suggests that the environmental condition in which conjugation occurs could affect the genetic environment of the *mcr* gene in a plasmid, potentially causing the genetic context of the gene to change in different plasmid replicons. 

Other predominant ISs and transposons involved (having flanked/surrounded *mcr*) in the acquisition/transfer of *mcr*-1 by different plasmid types in *Enterobacterales* include IS*CR1*, IS*Ecp1*, and IS*2* [163]. Transposons, including IS*903*, IS*Kpn*, and IS*Ec36*, were downstream and upstream of *mcr*-10 in the IncFII plasmid in *Enterobacter* isolates [139]. In *Aeromonas* isolates, *mcr*-3.15 was flanked upstream by IS*Kpn3* and IS*As17* [235], and transposon Tn*6518* flanked *mcr*-3.6 [168], while a *Proteus* isolate possessed IS*As13* and IS*Aeca6* (of IS*3* family) upstream and downstream of *mcr*-3.10 in the IncI plasmid, respectively [169]. The transposon Tn*5939* and ISs (such as IS*Kpn26* and IS*903B*) were involved in capturing, transposing, and disseminating *mcr*-8 and *mcr*-10 in *K*. *pneumoniae* strains [51,166]. These findings suggest that in the Chinese poultry sector, the diversity of MGEs (ISs and transposons) facilitates the spread of *mcr* genes acquired/mobilized from heterogeneous sources. Notably, IS*Ecl1* was inserted in the intergenic region of *mcr*-8.2 and *copR* in a *K*. *pneumoniae* isolate [51], as occurred in swine *K*. *quasipneumoniae* [246]. This supports the speculation that IS*Ecl1* insertion occurred before *mcr*-8.2 mobilization and has no association with the translocation of *mcr*-8.2 [246]. Remarkably, IS*Asp1* and IS*Ecp1* flanked *mcr*-1 and *bla*_CTX-M-55_ in the IncI2 plasmid in an *E*. *coli* isolate [141] and *S*. *Albany* strain [165], indicating that different ISs are involved in the colocation of *mcr*-1 with ESBL genes on the same plasmid. Furthermore, in the chromosomes of *E*. *coli* and *Enterobacter* isolates, as well as in non-self-transmissible *mcr*-10-IncFII in *K*. *pneumoniae*, the tyrosine recombinase gene *xerC* (which mediates the mobilization of genetic elements) and *insCinsD*-like region were upstream and downstream of *mcr*-10, respectively [167], suggesting that the structure *xerC*-*mcr*-10-*insCinsD*-like is a conserved region in the chromosomes of *mcr*-10-bearing *Enterobacterales*. However, IS*Ec36* transposase Ins*C* was upstream and downstream of the conserved site, suggesting that the area surrounding *xerC*-*mcr*-10 is a high-frequency region for the insertion of MGEs, thus suggesting that there are diversified paths for *mcr*-10 transfer [167]. Nevertheless, nonmobile genetic elements such as the class 1 integron encoded by the integrase gene *Int*I was associated with *mcr*-1 in some *E*. *coli* isolates [59,60,108]. 

The *mcr*-positive isolates coexpressed additional resistance genes (Table 1). Thus, the Chinese poultry sector is a potential reservoir of cocktails of multiresistance genes. The beta-lactam resistance gene (*bla*) and the florfenicol (an animal-specific drug) determinant *floR* dominated amongst the resistance genes coexpressed in *mcr*-positive isolates [44,89,97,109] (Table 1 and Table S1), suggesting that β-lactam and florfenicol resistance select for COL resistance [247]. The selection pressure for such selections/gene mobilization is due to the massive use of β-lactams and florfenicol in Chinese livestock for prophylaxis and treatment, with 3236 tonnes of β-lactams used in animals in 2018 alone in China [248]. Specifically, *E*. *coli* isolates coexpressed *mcr* with not less than 90 additional resistance genes (Table 1), including genes encoding ESBLs (*bla*_CTX-M_, *bla*_SHV_, and *bla*_OXA_ variants) [46,61,89,106,108,109,136,142,143,144,146,147], pAmpC (*bla*_CMY_ gene) [143], and carbapenemases (*bla*_OXA_ and *bla*_NDM_ genes) [60,62,105,144,147,148,149,150]. These genes could confer resistance to 10 antimicrobial classes/categories. They also coexpressed plasmid-encoded genes conferring resistance to fluoroquinolones (*qnrS* and *oqx*), fluoroquinolones/aminoglycosides (*aac(6′)-Ib-cr*) [109], and fosfomycin (*fosA*) [46,63,94,105,106,147,148], thus indicating that the isolates are potential multi- to pandrug-resistant organisms. 

The *E*. *fergusonii* isolates coexpressed *mcr* with 43 additional resistance genes (including ESBL genes—*bla*_OXA-10_ and *fosA*) conferring resistance to 10 antimicrobial classes [137,138,163]. *Klebsiella* isolates contained >40 additional resistance genes (including *armA*, encoding 16S rRNA-methylase, conferring high-grade aminoglycoside resistance; ESBL; carbapenemase—*bla*_NDM-5_; *fosA*; and PMQR genes) conferring resistance to eight antimicrobial classes [49,133,135,167]; *Cronobacter* isolates harboured *mcr* and 6 additional resistance genes (including PMQR; carbapenemase—*bla*_NDM-9_; ESBL—*bla*_CTX-M-55/65_; and *fosA* genes) conferring resistance to four antimicrobial classes [158]; and *Aeromonas* isolates contained *mcr* and 21 extra resistance genes (including PMQR, *aac(6ʹ)-Ib-cr*, and ESBL genes) conferring resistance to eight antimicrobial classes [168,235], while 33 other resistance genes (including ESBL and PMQR genes) also conferring resistance to eight antimicrobial classes were coexpressed with *mcr* by *Rauoltella* isolates [86] (Table S1). Thus, the Chinese poultry sector constitutes a reservoir of multi- to pandrug-resistant organisms. Notably, some *mcr*-1-positive *E*. *coli* isolates coexpressed the chromosomal gene *pmrB* [62], implying that upregulated mutations in chromosomal genes and *mcr* confer COL resistance simultaneously. Remarkably, more than 10 additional antimicrobial resistance genes were colocated with the *mcr* gene on the same plasmids in some *E*. *coli* isolates [92,146], suggesting the possibility of megaplasmids driving COL and extensive-to-pandrug resistance in the Chinese poultry industry. 

Interestingly, a considerably high number of *mcr*-1-positive ESBL-, pAmpC-, and carbapenemase-producing organisms, as well as a high abundance of the corresponding genes, were detected in all samples collected over an entire sampling period during the ban enforcement (2017–2019) [46,59,139], suggesting that the ban on prophylactic COL use has had a limited influence on the prevalence of unrelated antibiotic resistance and that, even after the ban, organisms coproducing MCR-1, ESBL, and carbapenemase could still be circulating in the poultry environment for a considerably long time. The presence of *mcr*-1-positive ESBL-/carbapenemase-producing organisms during the ban enforcement may be related to the fact that, though the ban policy was gazetted November 2016, prophylactic COL use possibly continued until the enforcement of the ban began in April/May Therefore, the impact of a ban policy is best evaluated at least a year after the strict enforcement of the policy. Moreover, therapeutic COL use has continued in the Chinese livestock sector. 

Most worrisome is that some *Enterobacterales* coexpressed *mcr* with plasmid-mediated transmissible genes *tet*(X) and *tmexCD-toprJ*, conferring high-level tigecycline resistance. *E*. *coli* isolates from pigeons coexpressed *mcr*-1 with *tet*(X4) and *bla*_NDM-1_/*bla*_NDM5_ [92], while *K*. *pneumoniae* isolates from chickens contained *mcr*-8, *mcr*-10, *tmexCD1-toprJ1*, and *bla*_CTX-M-27_ [133,151,166,167]. This suggests that the Chinese poultry sector is a potential reservoir for the emergence and spread of pandrug-resistant organisms, posing emergent challenges to antimicrobial therapy. Tigecycline is a broad-spectrum third-generation (belonging to the glycylcycline class) tetracycline antibiotic used as a salvage drug to treat deadly human infections caused by MDR, XDR, and PDR Gram-positive and Gram-negative organisms, including carbapenem- and COL-resistant infections [101]. Exposure to antimicrobials, especially COL, could exert selection pressure for the emergence of a stable plasmid coharbouring *mcr*-1, *tet*(X), and *tmexCD-toprJ*, thereby accelerating the transmission of these genes among bacteria [249]. 

Phenotypically, some *mcr*-1-positive *E*. *coli* isolates exhibited exclusive COL resistance [135], confirming that the *mcr* gene does not necessarily confer resistance to other antimicrobial agents [23]. Interestingly, susceptibility to COL was exhibited by some *mcr*-1-positive strains among *E*. *coli* isolates [108] and *Salmonella* in which IS*26* interrupted *mcr*-1, making it inactive [153]. This suggests that the *mcr* gene plays other roles aside from COL resistance. It also supports that selective primary isolation using media supplemented with COL and/or detecting COL resistance solely by phenotypic methods potentially results in the silent spread of the *mcr* gene [18]. Thus, molecular characterization is critical in the surveillance of MGCB. Moreover, some *mcr*-9-bearing salmonellae exhibited considerable COL resistance (COL MIC < 4 µg/mL) [99], suggesting that these isolates might possess the *qseBC* two-component system, which is thought to induce COL resistance in *mcr*-9-bearing organisms [250]. This suggests that, contrary to earlier belief, outbreaks associated with *mcr*-9-positive COL-resistant *Salmonella* could be a threat to public and animal health. Thus, there is a need for antimicrobial susceptibility testing (AST) even after WGS and for the further investigation of COL resistance in *mcr*-9-positive isolates without the *qseBC* two-component system. 

Unsurprisingly, multidrug to extensive drug resistance patterns were demonstrated by most of the isolates that coexpressed *mcr* with ESBL, pAmpC, and carbapenemase genes, including isolates positive for TIG, fosfomycin, and PMQR genes [60,63,91,108,141,142,149,158]. This shows that potential superbugs capable of jeopardizing antimicrobial therapy in infected/colonized animals and persons are circulating in China. However, some of the organisms, including TIG-resistant strains, as well as isolates with megaplasmids, remained susceptible to highly important and critically important antibiotics, such as TIG, carbapenems, cefepime, cefoxitin, aztreonam, amikacin, and doxycycline, among others [63,91,92,141,158]. This means that some non-last-resort antibiotics could be useful in treating infections associated with *mcr*-bearing superbugs. Therefore, it is crucial to conduct AST before antibiotic prescription/administration and even after molecular characterization. This practice is critical to preserve HP-CIAs only for managing human MDR and XDR infections. Nonetheless, per antimicrobial stewardship principles [251], in cases where antibiotic therapy must be instituted early, a broad-spectrum antibiotic, which should be de-escalated to a narrow-spectrum agent, could be used in the meantime while awaiting the results of AST. 

Because extended-spectrum cephalosporins, carbapenems, and fosfomycin are not approved for use in livestock in China [149], the ESBL, pAmpC, carbapenemase, and fosfomycin determinants in the isolates were likely acquired from anthropogenically impacted sources. Moreover, genes encoding ESBL, pAmpC, carbapenemase, and fosfomycin resistance have mostly been detected in isolates from human–environmental sectors in China [18,149,158]. Furthermore, TIG has never been used in livestock in China or anywhere around the globe, but the overuse of older (first and second)-generation TETs such as oxytetracycline, chlortetracycline, and doxycycline in human and veterinary (especially livestock husbandry) medical practices, especially in LMICs, for over 60 years led to the development and emergence of plasmid-mediated transmissible TIG resistance [101]. So, as it is, carbapenems and TIG should not be approved/licensed for use in livestock to preserve their efficacy for human medicine. In addition, the use of TETs in human and veterinary medicine should immediately be reconsidered to curb plasmid-mediated TIG resistance spread. 

The heavy-metal resistance genes *qac* and *ter*, conferring resistance to quaternary ammonium compounds and tellurium (which is highly toxic to bacteria), respectively, existed in some *mcr*-1-positive *Enterobacterales* [44,59,153,154,164]. This suggests that potentially *mcr*-positive disinfectant/biocide-resistant organisms are circulating in the Chinese poultry environment. Such organisms could evade farm biosecurity lines, such as disinfectant foot dips and hand washes, and find their way into the public domain, thereby posing a challenge to public health. *mcr* gene-bearing disinfectant-resistant *E*. *coli* has been already isolated from human setting in China [252]. The heavy use of disinfectants (which usually contain copper and silver) in livestock farms, as well as the regular use of zinc, copper, and other heavy metals as feed supplements for poultry birds (including waterfowl) in China [105,253], possibly exerts selective pressure for the acquisition of heavy-metal resistance genes. Heavy metals and many biocides can co-select for AMR, including COL resistance, stimulate HGT, and alter the antibiotic resistance dynamics in a particular natural ecosystem [254]. 

In vitro studies showed that through the conjugation mechanism, *mcr* and other antimicrobial determinants on conjugative plasmids in the isolates are transferrable to other organisms [89,91,94,95,135]. Expectedly, isolates possessing type IV secretion systems (T4SSs), such as *virB* and pilus expression genes (*pilM*, *pilV*, and *pill*), among other conjugation machinery, successfully transferred ARGs to recipient organisms [94,255]. However, in some *E*. *coli* isolates, ISs/transposons truncated the T4SS gene, which affected the efficiency of conjugative plasmid transfer [150]. In addition, *mcr*-1-IncHI2 in *S*. Typhimurium isolates was untransferable [100], suggesting that a plasmid-borne *mcr* gene must not necessarily be spread by horizontal transmission, but rather clonally disseminated if the *mcr* gene is borne on a non-conjugative plasmid. 

The *mcr* transmissibility rate (HGT/conjugation frequency) appears to have been majorly influenced by the type of organism and the *mcr* gene. *E*. *fergusonii* isolates transferred *mcr*-1 to the recipient organism at a frequency of ~10^−4^ to 10^−2^ [137,163]; *E*. *coli* isolates demonstrated the highest propensity to transfer the *mcr* gene, having cotransferred *mcr*-1 (associated with IncX4, IncI2, and IncHI2 plasmids) with one or more non-coresident genes (including *bla*_NDM-1_, *bla*_NDM-4_, *bla*_NDM-5_, *bla*_CTXM-groups_, and *fosA*) to recipient organisms at frequencies ranging from 9.0 × 10^−10^ to 5.0 × 10^−3^ [45,105,106,142,159,160,179]. *Salmonella* isolates cotransferred *bla*_CTX-M-14_ with *mcr*-1-IncX4 and *mcr*-1-IncHI2 to recipient organisms at frequencies of 1.76 ± 0.58 × 10^−5^ and 2.1 × 10^−4^ to 3.9 × 10^−6^, respectively [43,100]. *Klebsiella* isolates conjugatively transferred *mcr*-7, *mcr*-8, and *tmexCD1-toprJ1* at frequencies ranging from 1.20 ± 0.326 × 10^−7^ to 4 × 10^−4^ [51,166,167], while *Rauoltella* isolates transferred *mcr*-8 to a recipient organism at frequencies of 4.17 ± 1.35 × 10^−7^ to 3.09 ± 1.29 × 10^−7^ [86]. Thus, through horizontal transmission, the isolates could spread multi- to pandrug resistance to other organisms, thereby jeopardizing antimicrobial therapy. Notably, *mcr*-1 and the carbapenem determinant *bla*_NDM-4_ were in the IncHI2/ST3-type plasmid of a chicken *E*. *coli* transconjugant [159], suggesting that coresident plasmids containing *mcr* and another resistance gene are more stable, confer more of a fitness advantage, and are easier to transfer and cotransfer than a single plasmid [29]. Therefore, coresident *mcr* plasmids could seriously threaten clinical therapy and public health. However, due to its low fitness cost, IncI appeared to be the commonest plasmid disseminating extensive and pandrug resistance in the Chinese poultry sector [256]. 

The host range of the plasmid also appears to have determined *mcr* transmissibility. For example, *mcr*-1-IncY conjugated with the genome of only one of three different recipient organisms (genera) at a frequency of 10^−4^ to 10^−1^ transconjugants per cell [155], suggesting that IncY is not a broad-host plasmid. The *mcr* variant seems to have also affected the HGT frequency since *mcr*-1-IncI2 conjugated with the genome of the recipient organism at frequencies ranging from 10^−1^ to 3.87 × 10^−2^ [25,142], whereas *mcr*-1.3-IncI2 was transferred at a frequency of 2 × 10^−3^ [144]. The HGT frequency also seems to have depended on the plasmid replicon type since *mcr*-1-IncX4 was transferred more rapidly than *mcr*-1-IncH [257]. It has been reported that *pixR*, which is specific to the IncX4 plasmid, increases its transmissibility, thereby promoting the invasion and persistence of *mcr*-1 [258]. This could explain the global dissemination and high prevalence of *mcr*-1-IncX. However, Li et al. [5] reported that *mcr*-1-IncHI2 in avian salmonellae was conjugatively transferred at a significantly higher rate (1.43 × 10^2^ colony-forming units (cfu)/g faeces) than *mcr*-1-IncX4 (0.3 × 10^2^ cfu/g faeces) in the mouse gut. This suggests that host factors, such as hormones, feed in the gut, receptors, and so on, can affect the transmissibility of the *mcr* gene in live animals. Lamentably, *mcr* plasmids (especially *mcr*-1 plasmids) are very stable, enabling the high survival of carriers exposed to COL, and have high transference rates, thus underlining the high incidence of *mcr*-1-positive isolates in the Chinese poultry sector. Aside from the conjugation mechanism of HGT/acquisition, *E*. *coli* isolates acquired *mcr* by a transformation mechanism since plasmid-borne *mcr*-1 in some isolates could only be transferred by transformation into the recipient organism [148]. 

The population structure of the *mcr*-bearing *E*. *coli* isolates is extensively diversified, heterogeneous, and without clonal restriction, having belonged to more than 170 sequence types (STs), including zoonotic pandemic/international high-risk extraintestinal pathogenic *E*. *coli* (HiR-ExPEC) clones ST10, ST156, ST48, ST117, ST58, ST88, ST117, ST410, ST167, ST127, ST131, ST69, ST354, ST95, ST617, ST38, ST648, and ST410 [259] (Table 1). Some of these HiR-ExPEC clones have established traits, such as ST10—the largest reservoir of *mcr*-1; ST131—an epidemic clone with *bla*_CTX-M-15_; ST410—a pandemic clone for cross-sectorial ESBL gene dissemination; and ST167—a high-risk clone with *bla*_NDM_ [87]. More worrisome is that *E*. *coli* isolates that coexpressed *mcr* with ESBL, pAmpC, and carbapenemases genes belonged to various HiR-ExPEC clones dominated by ST10 [63,159]. Worst of all, some of the isolates belonging to ExPEC clones, including ESBL/pAmpC/carbapenemase producers, harboured virulence genes that are typical markers of Uropathogenic *E*. *coli* (UPEC—*iut* and/or *papC/A*), Enterohaemorrhagic *E*. *coli* (EHEC—*stx1*), Enteropathogenic *E*. *coli* (EPEC—*eae*), and Enteroaggregative *E*. *coli* (EAEC—*aafII* and *ast*, encoding heat-stable EAST-1 toxin associated with human diarrhoea) pathotypes [147,164,179]. Moreover, the *mcr*-1-positive *E*. *coli* isolates are phylogenetically diversified, belonging to phylogroups A, B1, B2, and D (Table 1), further suggesting that they are virulent and commensal strains adaptable to diverse ecological niches since ExPEC strains mostly belong to phylogroups B2 and, to a lesser extent, D [260]. It has been noted that *mcr* expression increases bacterial survival fitness and virulence [261]. Thus, in China, there are diverse virulent multidrug to pandrug resistance *mcr*-gene-bearing *E*. *coli* clones that could cause intestinal and extraintestinal diseases that are difficult to nearly impossible to treat in poultry birds. Accordingly, several Chinese investigators isolated *mcr*-gene-positive ESBL-/carbapenemase-producing *E*. *coli* from birds with colibacillosis [49,59,60,61,62,63,158,159,161,179].

Heterogeneity also existed among *Aeromonas* isolates belonging to ST514 and ST513 [85]. Similarly, the transmission of *mcr*-1 among nontyphoidal salmonellae from Chinese poultry is not clonally/serotype-restricted, as a diversity of serovars, including ST17 *S*. Indiana, ST34 *S*. Typhimurium, ST5399 *S*. Ngor, and ST2529 *S*. Goldcoast, among others, were detected [43,44,98,100]. ST34 appears to be the predominant *mcr*-1-bearing *Salmonella* clone circulating in the Chinese poultry sector, and it remains the dominant *mcr*-1-harbouring clone associated with community diarrhoea in mainland China [43,262]. This suggests that the ST34 *S*. Typhimurium clone is exchanged via the farm-to-table route, probably due to increased intensive livestock farming. It is speculated that ST34 disseminated into human communities, especially from the Chinese pig sector rather than from the poultry sector since human ST34 strains are closely related to swine isolates [263]. ST34 is known to possess virulence genes and exhibit a multidrug resistance phenotype; this may be why it emerged as a major epidemic clone causing nosocomial infections and foodborne outbreaks globally [43,264]. 

#### 4.1.2. Southern Asia

##### India

Among 19 COL-resistant *E*. *coli* isolates, 2 (10.5%) strains carrying *mcr*-1 and 11 other resistance genes (including a PMQR gene) conferring resistance to seven antimicrobial classes (Table 1) on IncX1 and IncHI2 plasmids were detected [110], suggesting that diverse plasmids have spread *mcr*-1 in the Indian poultry sector. COL is heavily used in the Indian poultry sector [232,265]. Fortunately, the use of COL as a growth promoter in food-producing animals has been prohibited since 2019 in India [19]. Although India accounted for 2.2% of global veterinary antimicrobial use in 2017, a negligible decrease in consumption to 2.1% has been projected for this country by 2030 [16]. Regrettably, MGCB have been detected in isolates from humans and the environment in India [266].

##### Pakistan

A total of 267 (32.5%) strains carrying *mcr* genes (*mcr*-1—250 *E*. *coli*, 12 *K*. *pneumoniae*, and 1 each for *Proteus mirabilis* and *P*. *aeruginosa*; *mcr*-2—2 *Proteus mirabilis*) were detected among 822 isolates [8,172,174,175,176,177,178,179], suggesting that the *mcr* gene has spread among *Enterobacterales* and non-fermenting organisms in the ESKAPE group in the Pakistani poultry sector. Various plasmids (such as IncX4, IncI, IncF, IncH, IncP, IncY, and others) and ISs, including IS*Apl1*, IS*CR1*, and IS*Ecp1*, were associated with *mcr* genes in the *E*. *coli* isolates (Table 1), indicating that diverse MGEs drive COL resistance in the Indian subcontinent. In an *E*. *coli* isolate, *mcr*-1 was beside the *sdrl* gene (serine–aspartate repeat surface protein known to bind collagen) with yet unknown relevance in the IncI plasmid [174], suggesting that apart from transposons and ISs, there might be other genetic elements translocating *mcr*-1 into IncI plasmid.

The *mcr*-1-positive *E*. *coli* isolates were extensively diversified, belonging to 66 STs (dominated by ST361 and ST1035) (Table 1), including HiR-ExPEC clones ST10, ST69, ST95, ST744, ST131, ST88, ST410, ST354, and ST135 [259]. They also harboured virulence genes, including markers of APEC (*ompT*), EHEC (*astA*), and UPEC (*papC*), and coexpressed more than 20 additional resistance genes, including ESBL, carbapenemase (*bla*_IMP_, *bla*_OXA-48_ and *bla*_NDM-1_), and *tet*(X4) [67,178] (Table 1), conferring resistance to nine antimicrobial classes. Thus, the transmission of *mcr*-1 among the *E*. *coli* isolates was non-clonal. However, there appeared to be clonal dissemination of *tet*(X4) [178]. Although the *tet*(X4)-positive isolates were susceptible to meropenem [178], they pose threat to antimicrobial therapy, having transferred both *mcr*-1 and *tet*(X4) to recipient organisms at high frequencies of 1.59 ± 0.2 × 10^−6^ to 5.23 ± 0.2 × 10^−6^ and 1.53 ± 0.8 × 10^−1^ to 5.02 ± 0.4 × 10^−1^, respectively [178,179]. Thus, the Pakistani poultry industry is a potential source of multi- to pandrug-resistant *E*. *coli* clones that can potentially cause considerable economic losses to poultry farmers. Sadly, both *mcr*-1 and *tet*(X4) have dispersed to a diversity of organisms in the human–environmental ecosystem in Pakistan [101,267]. Pakistan is among the Asian countries in which COL use as a feed additive/growth promoter is high. Between 2017 and 2020, a net total of 275.5 tonnes (68.9 tonnes per year) of COL was imported into Pakistan for animal use, with an estimated 2.5 mg/kg of COL used in that period [8]. Although COL is considered an impermissible antibiotic growth promoter in Pakistan, there is no national legislation prohibiting the use of antibiotic growth promoters in this country [8].

##### Bangladesh 

Out of 3295 *Enterobacterales* isolated from poultry birds and the environment, 533 (16.2%) organisms harbouring *mcr* genes (*mcr*-1—421 *E*. *coli*, 12 *Salmonella*, 17 *Proteus*, 10 *Klebsiella*, 2 *Enterobacter*, and 1 *Shigella*; *mcr*-2—2 *Proteus mirabilis* and 1 each for *E*. *coli*, *K*. *pneumoniae*, *Salmonella*, and *Enterobacter*; and *mcr*-3—11 *E*. *coli*) were [39,40,41,53,65,66,88,93,181,182,183]. This suggests a considerably high incidence of a diversity of bacteria spreading various *mcr* gene types in the Bangladeshi poultry sector. Interestingly, a low proportion (only two *Proteus mirabilis*) of the *mcr*-positive isolates coexpressed *mcr*-1 with *mcr*-2, which increased the phenotypic COL resistance (higher MIC values) of the isolates [93]. Therefore, combining two *mcr* genes may enable bacteria to emerge as HiR bacterial clones [93]. Notably, the Bangladeshi poultry sector is a potential sink for emerging and disseminating diverse *mcr* genes since novel *mcr*-1 variants, including *mcr*-1.23, *mcr*-1.24, and *mcr*-1.25, were detected in *Salmonella* and *E*. *coli* isolates from the sector (GenBank Accession Numbers: NG_067235; NG_067236; NG_067237). In the *E*. *coli* isolates, *mcr*-1 flanked by single or multiple copies of IS*Apl1* and 45 additional resistance genes (including ESBL and PMQR genes) conferring resistance to nine antimicrobial classes on various plasmids (ColRNAI, IncHI1, IncFIB, IncN, and IncFIA, among others) were present [53,65] (Table 1). The *E*. *coli* isolates were extensively diversified, belonging to 17 STs [39], including HiR-ExPEC clone ST354 [259], and they possessed virulence genes, including markers of EPEC (*eaeA*) and EAEC (*ast*). Thus, various MGEs resulted in a diverse range of virulent MDR to XDR *E*. *coli* clones in Bangladesh. Although the use of all COL preparations in veterinary medicine has been banned in Bangladesh since March 2022 [8], COL is still frequently used in the Bangladeshi poultry sector. Accordingly, Islam et al. [93] reported a statistically significant association between COL usage (mostly among non-professionals for treatment, prophylaxis, and growth promotion) and *mcr* carriage by *E*. *coli* isolates. Unfortunately, MGCB have disseminated through the food chain into the Bangladeshi human population [40,66].

##### Nepal

Of 305 *E*. *coli* isolated between 2017 and 2019, 66 (21.6%) strains carrying *mcr*-1 on InK/B and IncI plasmids were detected [69,70,71], suggesting that promiscuous *mcr*-1 plasmids have widely spread in the Himalayas. COL is used for prophylaxis and growth enhancement in Nepalese poultry [71]. The *mcr*-positive *E*. *coli* isolates coexpressed virulence genes, including a marker for EAEC (*ast*) and 22 other resistance genes, including carbapenemase (*bla*_OXA-48_), ESBL, and PMQR genes, conferring resistance to seven antimicrobial classes [69,71] (Table 1). Thus, the Nepalese poultry sector is a potential reservoir of superbugs that could threaten public health, especially for tourists visiting these regions. Lamentably, *mcr*-1-habouring carbapenemase-producing *E*. *coli* have diffused into the human population in Nepal [71]. Being a landlocked nation and sandwiched between two emerging economies (China and India), MGCB can be easily transported to and from Nepal, posing a threat to the health of its population and that of its neighbours. 

#### 4.1.3. Western Asia (Middle East)

##### Lebanon

Among 617 Enterobactericeae isolated from 2015 to 2018, 346 (56.1%) strains carrying *mcr*-1 (315 *E*. *coli* and 31 *K*. *pneumoniae*) on IncX4 (majorly), IncI2, and IncHI2 plasmids and class one integrons were detected [47,131,184,185,186,187]. This indicates a high incidence of *mcr*-1 in the sector and the involvement of diverse genetic elements (mobile and nonmobile), especially the IncX4 plasmid, in the dissemination of COL resistance in Lebanese poultry for at least eight years. The isolates coexpressed *mcr* with 53 additional resistance genes, including ESBL, pAmpC, *fosA*, and PMQR genes, conferring resistance to nine antimicrobial classes, and they were extensively diversified, belonging to 35 STs (dominated by ST1140 and ST1011) (Table 1), including HiR-ExPEC clones ST10, ST354, and ST101, as well as ST2705, which is an *mcr*-1-positive clone that has also been isolated from human patients in Lebanon [187]. Thus, Lebanese poultry is a potential source of the spread of MDR and XDR *E*. *coli* clones capable of causing difficult-to-treat diseases. 

The high prevalence/endemicity of *mcr* in the Lebanese poultry sector requires urgent attention because of the mobility of refugee populations and the close geographical proximity of Lebanon to many European, African, and Middle Eastern countries and the Mediterranean Sea [23,268]. COL is extensively used unregulated in human medicine and as a growth enhancer and prophylactic in food animals in Lebanon [47,184,186]. This causes the sporadic emergence and development of COL resistance in the Lebanese poultry sector [236]. Nonetheless, humans may be a major source of MGCB in the Lebanese livestock sector since the amount of COL imported/used for the treatment of human infections in Lebanon increased by approximately 5 times from 72,135 flacons (1 MIU of COL per flacon) in 2010 to 348,500 flacons in 2017 [185]. 

##### Iraq

Amongst 200 *Acinetobacter baumannii* isolates, 26 (13%) strains that coexpressed *mcr*-1 with virulence genes and 17 other antimicrobial determinants (including carbapenemase—*bla*_VIM_, *bla*_IMP_, and *bla*_SIM_—and ESBL genes) conferring resistance to five antimicrobial classes were detected [121] (Table 1). This shows that the poultry sector in the Middle East and North Africa (MENA) region is a potential reservoir of superbugs that could pose a threat to public health (since *A*. *baumannii* is a major nosocomial pathogen worldwide), especially to tourists visiting the region. Carbapenems are not known to be used in the livestock sector in Iraq; thus, the presence of carbapenem determinants in the isolates suggests the possible diffusion of the organisms from humans (due to the prolonged war in Iraq) to animal settings. Regrettably, *mcr*-bearing *A*. *baumannii* has been isolated from humans in Iraq [269]. 

##### Türkiye 

Among 203 *E*. *coli* isolated between 2014 and 2019, 5 (2.4%) strains carrying *mcr*-1 on IncI2 and IncX plasmids and belonging to ST3941 (associated with human bloodstream infections), ST1049, and ST6094 clones were detected [111,112,270]. This shows that for more than half a decade at least, in the Greater Middle East region, *mcr*-1 has been spreading among diverse *E*. *coli* clones, albeit at a low prevalence. The isolates coexpressed *mcr*-1 with 17 other resistance genes, including ESBL and PMQR genes, conferring resistance to seven antimicrobial classes, as well as virulence genes, including a marker for EAEC (*ast*) [111,112] (Table 1). Interestingly, the isolates were closely related to *mcr*-1-positive *E*. *coli* strains from Asia and Europe, suggesting the possible intercontinental dissemination of the organisms, as Türkiye is a transcontinental country on both continents. Unfortunately, MGCB have been isolated from humans in Türkiye [271].

Notably, none of the 330 *Enterobacterales* isolated between 2012 and 2018 from poultry birds in Iran (also an LMIC in the Greater Middle East region) harboured *mcr*-1, *mcr*-2, *mcr*-3, or *mcr*-4 [272,273]. However, the chromosomal COL resistance genes *mgrB*, *pmrA*, *pmrB*, *phoP*, *phoQ*, *crrA*, and *crrB* existed in the organisms, indicating diverse chromosomal COL resistance mechanisms.

#### 4.1.4. Southeastern Asia

Most of the countries in Southeastern Asia (SEA) are heavy producers/exporters of poultry and aquaculture products. However, as in many other LMICs, there is currently no strict requirement for a prescription before the use of antimicrobials in animals in most countries in the SEA region [15]. The heavy use of COL and non-polymyxin antibiotics (an estimated overall average of 74.4 mg of in-feed antimicrobials was used to raise every 1 kg of live chicken in SEA, in addition to polymyxins, mostly along with other antimicrobials in the form of premixes) in the poultry sector exerted selective pressure for *mcr* gene acquisition in poultry sectors of the SEA region [274,275]. 

##### Lao People’s Democratic Republic

Out of 175 COL-resistant *E*. *coli* isolates, 37 (21.1%) strains carrying *mcr* genes (*mcr*-1—34 *E*. *coli*; *mcr*-3—3 *E*. *coli*) on IncX4, IncI2, IncP1, IncH, and IncFII plasmids were detected [87,122]. This means that diverse promiscuous plasmids have widely spread various *mcr* genes, mostly *mcr*-1, in the Laotian poultry meat sector. Tn*6330* was associated with *mcr*-1 in IncFIA and IncP1 plasmids, but a single IS*Apl1* occurred downstream of *mcr*-1 in the IncI2 plasmid [87], implying the IS-derived mobility of *mcr*-However, IS*Apl1* was completely absent in other plasmid sequences [122], thus stabilizing *mcr*-1 in a diverse range of plasmid backgrounds. In *mcr*-3 plasmids, IS*Kpn40*, IS*26*, and IS*15DI* were present, but IS*15D1* was restricted to IncFII and IncFIB plasmids [87,122]. Additionally, Tn*As2* and *dgk* were, respectively, upstream and downstream of *mcr*-3, except in the IncFIA plasmid, where Tn*As2* was lost downstream of the *mcr* gene. This suggests that the loss of Tn*As2* does not affect the transmission of *mcr*-These genetic elements enabled the isolates to transfer *mcr*-1 to other organisms at frequencies of 1 × 10^−8^ to 1 × 10^−1^. The *mcr*-1 plasmids exerted a low fitness burden and remained stable for >15 days of serial passaging in antibiotic-free cultured media [87]. Thus, *mcr*-1 is maintained and rapidly disseminated in the bacterial population even without antimicrobial selective pressure. 

The isolates were extensively diversified, belonging to various phylogroups and 20 STs, including HiR-ExPEC clones ST10, ST69, and ST648, and they coexpressed virulence genes and 32 additional resistance genes, including ESBL, *fosA*, and PMQR genes, conferring resistance to nine antimicrobial classes. Notably, a low percentage of the *mcr*-positive isolates were TIG-resistant, but fortunately, they remained susceptible to amikacin, piperacillin–tazobactam, imipenem, and meropenem [87]. Sadly, MGCB have diffused into human communities, even colonizing travellers who visited Laos [122]. Since COL is rarely used in human medicine in Laos, its unregulated use in livestock possibly led to the development of MGCB in this country [87].

##### Thailand

Of 507 COL-resistant *Enterobacterales* isolated between 2013 and 2020, 76 (15%) organisms carrying *mcr*-1 (1 *Salmonella* and 75 *E*. *coli*) on the IncX4 plasmid were detected [83,130]. This implies that the *mcr-*1 IncX4 plasmid has been widely spread among a diversity of *Enterobacterales* in the Thai poultry sector for at least a decade. The *E*. *coli* isolates belonged to ST2973, and they harboured an ESBL gene—*bla*_CTX-M-14_ [130]—showing that the organisms might have diffused from the human to the animal setting and vice versa. COL has been used for prophylaxis and treatment in the Thai poultry sector, which is made up of >90% small-scale family backyard farms, often associated with high human–livestock interaction, poor hygiene, low biosecurity, and few barriers to potential contact or bacterial transmission between farm animals, humans, and wildlife [15,276]. Although a ban on the use of COL as a growth promoter in livestock was put in place in 2017 in Thailand, there appears to be little or no compliance by farmers since 94% of sampled livestock farms in Thailand used COL after the ban [19,276]. Nonetheless, the human–environmental ecosystem in Thailand has also been reported as a potential reservoir of MGCB [23].

##### Malaysia

Out of 262 *Enterobacterales* isolated between 2013 and 2020, 77 (29.4%) organisms (57 *E*. *coli* and 1 *Salmonella enterica*) bearing *mcr*-1 on various plasmids were detected [54,68,120,189,190,277] (Table 1). In some *E*. *coli* isolates, *mcr*-1 was flanked by IS*Apl1* or *nikB* [68], whereas in *Salmonella* isolates, IS*Apl1* was constantly upstream of *mcr*-1 in the IncI plasmid [189]. The *E*. *coli* isolates were extensively diversified, belonging to various phylogroups (A, B1, B2, and D) and eight STs, including HiR-ExPEC clones ST410, ST155, ST69, ST93, ST345, and ST117, and they coexpressed 22 other resistance genes, including ESBL, carbapenemase—*bla*_OXA-48_, *bla*_NDM_, and *bla_I_*_MP_—*fosA*, and PMQR genes, conferring resistance to eight antimicrobial classes (Table 1). These findings indicate that due to the extensive use of COL in the Malaysian poultry sector, MGEs (plasmids and ISs) have facilitated the wide spread of *mcr*-1 among diverse virulent *Enterobacterales* in Malaysia since at least a decade ago. Lamentably, *mcr*-1-positive organisms have already been associated with diseases in humans in Malaysia [54]. Fortunately, a ban has been placed on the use of COL for nontherapeutic purposes in livestock in this country [19]. 

##### Vietnam

Among 872 *E*. *coli* isolates, 278 (31.9%) strains carrying *mcr*-1 on IncI2, IcX4, and IncH, among others, were detected [84,113,114,115,116,117,118,119]. Notably, diverse *mcr* genes, including *mcr*-1, *mcr*-3, *mcr*-4, and *mcr*-5, were detected at a higher occurrence rate by metagenomics than by culture-based studies. This is probably due to the higher sensitivity of the targeted metagenomics approach, which detects ARGs from non-viable bacteria and extracellular DNA, unlike culture-based or sequenced-based metagenomics approaches [56]. 

The *mcr*-1-positive *E*. *coli* isolates were extensively diversified, belonging to 14 STs [117], including HIR-ExPEC clones ST354 and ST10 [259], and they coexpressed virulence genes, including markers of EAEC and UPEC, and other resistance genes, including ESBL/pAmpC and PMQR genes, conferring resistance to different antimicrobial classes (Table 1). Thus, due to the extensive use of COL-fortified poultry feed for growth enhancement in Vietnam [38,56], commensal/virulent *E*. *coli* clones coproducing MCR-1 and ESBL/AmpC have been present in the Arabian Peninsula for more than a decade. The organisms could pose a threat to public health by transferring *mcr*-1 and other resistance determinants to recipient organisms at high frequencies of 5.1 × 10^−3^ to 9.4 × 10^−5^ [119]. Lamentably, MGCB have disseminated into the human–environmental ecosystem in many countries in mainland Southeast Asia (Indochinese peninsula) and to persons who travelled to this region [226].

##### Indonesia

Out of 58 COL-resistant *E*. *coli* isolated in 2017, 13 (22.4%) *mcr*-1-carrying strains were detected in the Indonesian poultry meat supply chain [55]. The *mcr*-bearing isolates were multiresistant, and one was *E*. *coli* O157:H7, which is a zoonotic Shiga-toxin-producing serotype that causes haemorrhagic diseases, including haemorrhagic colitis and haemolytic uraemic syndrome, which are associated with high morbidity and mortality, especially in children, worldwide. This means that for at least six years, virulent MGCB capable of causing foodborne disease outbreaks have been spreading in the poultry sector in Oceania since Indonesia is a transcontinental country in Asia and Oceania. The involvement of *mcr*-1-positive COL-resistant *E*. *coli* O157:H7 could complicate diseases, posing a danger to public health. Humans acquire *E. coli* O157:H7 via the faecal–oral route from humans or reservoir animals and the consumption/ingestion of undercooked contaminated liquids and foods. 

Notably, none of the 13 *Salmonella enterica* isolated from chicken eggs in the Philippines harboured the *mcr*-1 gene [278].

### 4.2. Africa 

In Africa, livestock, especially poultry, plays an important role in socioeconomic conditions, as 250–300 million people depend on livestock for their income and livelihood, with livestock representing an average of 30% of the agricultural gross domestic product (GDP) and approximately 10% of the total GDP [31]. Unfortunately, the use of antimicrobials, including critically important ones such as COL, in poultry, as well as in other settings, remains largely unregulated in Africa [11,30]. Several factors compounding the problem of AMR in Africa have been well detailed in many studies [13,31,279]. The situation is further worsened due to factors such as weak regulatory frameworks, easy access to non-OTC medications, the continuous use of frontline medication in feed and water, and the treatment of animals without a diagnosis and often without AST, leading to the transmission of MGCB from livestock to human–environmental ecosystems [11,30,31,279]. Thus, information on MGCB in the African poultry sector is needed to develop efficient strategies for curbing the problem. 

Twenty-nine publications investigated the *mcr* gene in a total of 1207 isolates from the poultry sector in 7 of the 53 LMICs (among 54 countries) in Africa (The World Bank, 2021) (Table 2). Two of the studies investigated the presence of *mcr*-1 directly in chicken meat and faecal samples [280,281]. They detected *mcr*-1 in 357 strains (353 *E*. *coli* and 2 each for *Citrobacter* and *K*. *pneumoniae*), *mcr*-4 in 31 *Salmonella*, *mcr*-5 in 1 *E*. *coli*, *mcr*-8 in 1 each for *E*. *coli* and *K*. *pneumoniae*, and *mcr*-9 in 1 *E*. *coli* among the tested isolates. 

#### 4.2.1. Northern Africa 

##### Tunisia 

A total of 116 (55.8%) strains carrying *mcr*-1 on IncHI2, IncI, IncP, and IncFI plasmids were detected among 208 *E*. *coli* isolates [72,73,74,75,192,193,282]. IS*Apl1* and *pap2* were upstream and downstream of *mcr-*1, respectively [192], and class one integrons were associated with virulence genes, including markers of EHEC (*stx1* and *stx2*) and UPEC (*papC*), and 19 other antimicrobial determinants, including ESBL and pAmpC genes, conferring resistance to five antimicrobial classes in some *mcr*-positive isolates [74,282] (Table 2). The *mcr*-positive isolates were extensively diversified, belonging to 17 STs, including HiR-ExPEC clones ST10, ST117, and ST69 (Table 2). Thus, there is a high dispersal of *mcr*-1 driven by MGEs (plasmids and IS) and non-MGEs (integrons) among commensal and pathogenic *E*. *coli* clones in the Tunisian poultry sector. The findings also suggest that the organisms acquired virulence and *mcr* genes together. The transfer of plasmids from avian pathogenic *E*. *coli* (APEC) to avian commensal *E*. *coli* confers virulence [283], which can increase losses due to an outbreak of difficult-to-treat diseases in poultry. 

##### Algeria 

Of 290 *Enterobacterales*, 16 (5.5%) *E*. *coli* harboured *mcr*-1 on IncHI2, IncFV, and IncFIIK plasmids with IS*Apl1* downstream of *mcr*-1 [123,194]. This may suggest a low prevalence of *mcr*-1, even though COL is used as a metaphylactic treatment and as a feed additive in the Algerian poultry industry [123], and the involvement of diverse plasmids in the circulation of the gene. The low incidence could be an underestimation since COL-resistant isolates in the *Proteae* group (*Providencia*, *Proteus*, *Serratia*, and *Morganella*) were not tested for *mcr* genes on the grounds that they were intrinsically resistant to COL [123]. However, mutations in *pmrA* and *pmrB* were detected in the *mcr*-negative isolates [123]. 

The *E*. *coli* isolates were extensively diversified, belonging to 14 STs (dominated by ST48 and ST224), including HiR-ExPEC clones ST10 and ST648 [259], and coexpressed a diversity of virulence genes, including markers of ExPEC and pathogenicity islands (PAIs) PAI II_536_, PAI III_536_, and PAI IV_536_, which are markers of UPEC [123] (Table 2). This *mcr*-1-bearing UPEC has already diffused into the human setting in Algeria and MENA region [284]. Thus, tourists visiting the MENA region can be colonized by MGCB. Nonetheless, only three isolates were recovered from five *mcr*-1-positive poultry faecal samples [280], further supporting that when using isolation alone, the magnitude of *mcr* genes in an ecological niche could be underestimated, thereby favouring the dissemination of the gene [18,30].

##### Egypt 

A total of 113 (25.2%) organisms carrying *mcr* genes (*mcr*-1—111 *E*. *coli* and 1 *Citrobacter freundii*; *mcr*-9—1 *E*. *coli*) on IncX4, InI2, and IncFII plasmids with or without IS*Apl1* and *pap2* and the IncHI2 (containing Tn*6330*) plasmid were detected among 438 *Enterobacterales* isolated between approximately 2010 and 2019 [76,77,78,79,80,195,196,285]. This indicates a high prevalence of *mcr* genes, dominated by *mcr*-1, in the Egyptian poultry sector. It also shows that *E*. *coli* coproducing MCR-1 and ESBL/pAmpC has been in Africa for at least more than 13 years. Notably, the *mcr*-1-positive *E*. *coli* isolates recovered in 2010 [76], are now the oldest African *mcr*-positive isolates. Although the presence of *qseBC* in the *mcr*-9-positive *E*. *coli* isolates was not assessed, the isolates coexpressed only the trimethoprim determinant *dfrA15* [79]. This means that *mcr*-9 confers phenotypic COL resistance and that non-polymyxin antimicrobials such as trimethoprim, which is frequently used as a premix in poultry feed, could exert selection pressure for the acquisition of the *mcr* gene. *mcr*-9 is widely disseminated in Egypt since it has also been detected in *Enterobacter* isolates from beef patties and human patients in this country [30].

The *mcr*-1-positive *E*. *coli* isolates were extensively diversified, belonging to 12 STs, including HiR-ExPEC clone ST10, and coexpressed virulence genes such as the *sitABCD* operon encoding, among other things, hydrogen peroxide resistance, and 43 additional resistance genes, including *tet*(X7), carbapenem—*bla*_VIM_ and *bla*_NDM_—ESBL, pAmpC, and *fosA* genes, conferring resistance to eight antimicrobial classes (Table 2). Thus, the isolates were multi- to pandrug-resistant virulent *E*. *coli* potentially insusceptible to antiseptics. Remarkably, *mcr*-1 was colocated with *tet*(X7) in the IncHI2 plasmid, which co-fused with other plasmids, forming a multireplicon plasmid that was conjugatively transferred to recipient organisms, thereby increasing the transconjugant COL and TIG resistance by >16- and >32-fold, respectively [79]. This further shows that the recombination of plasmids could lead to the evolution of an *mcr* and *tet*(X) co-integrated plasmid, and this would impact antimicrobial therapy. The presence of these superbugs in the Egyptian poultry sector poses a threat to public health, including to consumers in countries that import poultry birds/products from Egypt, as well as tourists visiting this country. COL is used in the Egyptian livestock industry as a therapeutic and prophylactic agent [284], and the presence of the *mcr* gene in the sector for 13 years at least could mean that MGCB disseminated from animals to human–environmental settings in Egypt [30]. 

##### Morocco

Three (25%) strains carrying *mcr*-1 were detected among twelve COL-resistant *E*. *coli* isolates [81], indicating that pathogenic MGCB are spreading in Morocco due to extensive COL use in the poultry sector, thus posing a risk to public health, even to persons in Europe since this continent shares land borders with Morocco.

#### 4.2.2. Southern Africa

##### South Africa

A total of 70 (58.8%) organisms carrying *mcr* genes (*mcr*-1—39 *E*. *coli*; *mcr*-4—31 *Salmonella*) were detected among 119 multidrug-resistant Enterobacteriaceae isolated between 2016 and 2019 [82,198,199]. Screening for the *mcr* gene in only COL-resistant isolates could account for the high *mcr* prevalence recorded in the South African poultry sector. In *E*. *coli* isolates, *mcr*-1 was flanked downstream by IS*Apl1* in the IncI2 plasmid, which also contained five other resistance genes in different antimicrobial classes [198], while class 1 and 2 integrons were associated with the *mcr*-4 and β-lactam resistance genes, including ESBL genes [82]. These findings show that for at least seven years, mobile and nonmobile genetic elements have facilitated the wide dissemination of *mcr* genes in the South African poultry sector. However, *mcr*-1-positive isolates lacking pilus did not transfer the gene to the recipient organism [198], and thus, the gene could be maintained by vertical transfer. The presence of MGCB in the South African poultry sector could impact the country’s economy since poultry is the largest single contributor to the agricultural sector in South Africa [286]. It could also result in the dissemination of MGCB to other countries in the Southern African region since the South African poultry industry dominates regional production in the Southern African Development Community (SADC), accounting for 80% of poultry meat/egg production among the 15 member states [286]. In human medicine, COL use in South Africa has been strictly controlled, having been listed as a Schedule 4 substance, which makes it a prescription-only medicine that is available only from a pharmacy dispensary [287]. However, its use in veterinary medicine in South Africa has not been strictly controlled, with a massive four tonnes of COL/other antimicrobials consumed by livestock in 2015 [288]. Evidently, some South African farms that yielded *mcr*-1-positive isolates used COL for prophylaxis and treatment [199]. 

##### Zimbabwe

Out of 22 MDR *E*. *coli* isolates, only 1 (4.5%) ST10 strain that coexpressed *mcr*-1 with virulence and other resistance genes, including ESBL and PMQR genes, was identified [200]. This implies that the HiR-ExPEC clone is present in the Zimbabwean poultry sector, albeit at a low rate, which is possibly an underestimation. Zimbabwe is a major poultry bird/product importer in Africa; therefore, animal trade (especially importation from the MENA region) might be the source of the highly dispersed ST10 *mcr*-1-positive *E*. *coli*. 

#### 4.2.3. Western Africa 

##### Nigeria

Among 450 *Enterobacterales*, 37 (8.2%) organisms carrying *mcr* genes (*mcr*-1—31 *E*. *coli*, 2 *K*. *pneumoniae*, and 1 *Citrobacter werkmanii*; *mcr*-5—1 *E*. *coli*; and *mcr*-8—1 each for *E*. *coli* and *K*. *pneumoniae*) were recorded [34,201,202,289]. The IncX4 plasmid has been the only backbone for *mcr* detected in Nigerian poultry isolates [201,202]. The *mcr*-1-positive *E*. *coli* isolates were extensively diversified, belonging to 17 STs, dominated by STs in CC10, and they coexpressed *mcr* with 19 other resistance genes, including ESBL and pAmpC genes, conferring resistance to seven antimicrobial classes [34,201,202,289] (Table 2). Thus, *mcr*-1 is circulating more than other *mcr* genes among a diversity of multidrug-resistant to extensively drug-resistant virulent/commensal bacteria in Nigeria, and *mcr*-1-positive CC10 *E*. *coli* is successfully disseminated in Western Africa, as in other parts of the world [34]. Some *E*. *coli* isolates of yet unknown STs expressed a novel *mcr*-1 variant named *mcr*-1.22 [34], suggesting that the Nigerian poultry sector is a potential reservoir of emerging lineages of MGCB. Interestingly, some of the *mcr*-1-postive *E*. *coli* isolates elicited injuries to the internal organs of birds, where they conjugatively transferred *mcr*-1 to other *Enterobacterales* in the chickens’ gut at frequencies of 5.0 × 10^−7^ to 4.5 × 10^−6^ [24]. This means that the emergence of MGCB in the Nigerian poultry sector is a threat to the public health and economic growth of Nigeria since the poultry sector is the largest agribusiness, contributing about 25% of total national meat production and significantly to this country’s GDP [34]. 

Over three decades, COL has been imported into Nigeria from Asian and European countries and heavily used for prophylaxis, first-line therapy in metaphylaxis/treatment, and possibly growth enhancement for economic gains in the Nigerian livestock sector, especially poultry [8,34,289]. COL is mostly marketed by non-professionals in Nigeria [34], and due to poor knowledge about COL, farmers misuse the drug by adding COL to birds’ drinking water, often in plastic troughs, thereby resulting in subinhibitory concentrations of COL [34]. This practice consequently promotes the conjugation/transfer of *mcr* and other resistance genes [28]. Additionally, non-adherence to withdrawal periods by the farmers leads to the persistence of MGCB in the gut of birds that receive COL and humans that consume COL residues in poultry meat [32]. Nonetheless, COL has never been available/used in human medicine in Nigeria, but unfortunately, MGCB have disseminated into the human setting in this country [11,30]. 

#### 4.2.4. East Africa 

##### Tanzania

Three *E*. *coli* isolated in 2019 from chickens contained *mcr*-1 (https://www.ncbi.nlm.nih.gov/pathogens/antimicrobial-resistance/ (accessed on 27 May 2023)). Thus, MCGB have been present in the Tanzanian poultry sector, possibly due to the uncontrolled use of antibiotics, including COL, in food animals [287]. MGCB have disseminated into the human setting, even colonizing hotel workers and tourists in Tanzania, possibly due to the frequent empirical use of drugs without a prescription and the limited sanitation of the food chain [290]. 

### 4.3. South America 

Most countries in South America are major producers/exporters of poultry products, and there is lax control of the use of CIAs, including COL, in most of these nations. In addition, up to a 99% increase in antibiotic usage in food animals is expected in Latin American countries by 2050 [227]. Therefore, information on the magnitude of MGCB on the continent can create the needed impetus for tackling the AMR menace. 

Eighteen publications investigated the *mcr* gene in a total of 1520 reported isolates from the poultry meat production chain in 6 (60%) of 10 LMICs (among the 12 nations) in South America (The World Bank, 2021) (Table 3). A total of 360 strains were reported to harbour the *mcr*-1 gene (355 *E*. *coli*, 3 *E*. *fergusonnii*, and 2 *Salmonella enterica* serovar Schwarzengrund), while 5 *E*. *coli* were reported to contain the *mcr*-5 gene. 

#### 4.3.1. Northern South America

Ecuador

Of 326 *E*. *coli* isolates, 145 (44.5%) *mcr*-1-positive strains were detected [203,204,205,206,207]. This means that due to the frequent use of antimicrobial-/COL-fortified feeds in Ecuador [203,204], a high percentage of poultry isolates from the Andean region are acquiring the *mcr*-1 gene. Interestingly, some of the *mcr*-positive strains were recovered from the Amazonian Forest region, meaning that MGCB have even disseminated to pristine regions of the world. Some *mcr*-1-positive strains were ESBL/pAmpC producers recovered between 2013 and 2016 [203], suggesting that for at least a decade, organisms coproducing MCR-1 and ESBL/pAmpC have been present in Ecuador. Furthermore, neither extended-spectrum cephalosporins nor carbapenem is known to be used in the Ecuadorian livestock sector, yet some isolates from backyard birds coexpressed *mcr*-1 with genes encoding ESBL and carbapenem resistance (*bla*_OXA-48_ and *bla*_NDM_) [203,207]. This clearly shows that proximity to poultry birds is a risk factor for the zoonotic transmission of MGCB. The organisms can transfer *mcr*-1 to other organisms, having transferred the gene to a recipient organism at a frequency of 1 × 10^−4^ [205]. Although Ecuador banned the veterinary use of COL in January 2020 with the attendant decrease in the sales of COL as a growth promoter, there is still no enforcement of the policy [206].

#### 4.3.2. Western South America 

##### Peru

Among 274 *E*. *coli* isolated from 2018 to 2020, 44 (16.1%) isolates that coexpressed *mcr*-1 with 17 other resistance genes, including ESBL and PMQR genes, on various plasmids, such as IncI2, IncFI, and so on, were detected [208,209]. The isolates were extensively diversified, belonging to seven STs, including ExPEC ST746, ST10, and ST345, and they harboured virulence genes, including markers of APEC (*ompT*), EHEC (*ast*), EPEC (*eae*), and UPEC (*papC*) [209] (Table 1). Thus, the Peruvian poultry sector has a considerably wide circulation of *mcr*-1 among multiresistant commensal and virulent *E*. *coli* clones. The genetic context of *mcr*-1 in the isolates was Tn*6300* in an IncHI1A-IncHI1B hybrid plasmid and the bracketing of *mcr*-1 upstream and downstream by *pap2* and *nikB* in the IncI2 plasmid [209]. This implies that the homologous recombination of *mcr* plasmids facilitates the rapid transmission of *mcr* genes by TnPeru has a widespread intensive system of poultry farming and ranks fifth among poultry meat producers in Latin America, recording one of the largest per capita consumption rates of chicken meat in South America [291]. Thus, these virulent and multiresistant MGCB in Peru’s poultry sector could pose a health threat to Peruvians and tourists visiting the country. Antimicrobials, including COL, are frequently used as growth stimulators in the Peruvian poultry sector [291]. Luckily, the importation, sale, and use of COL for nontherapeutic purposes in Peru have been banned since 2019. 

#### 4.3.3. Eastern South America 

##### Brazil 

In the recent past, a massive amount (up to 10 g/ton of feed) of polymyxins/COL was used in livestock feed as a growth enhancer in Brazil [125]. However, this practice has been banned in the country since November 2016 [125,292,293]. However, Brazil is projected to use 7.9% of the global veterinary antibiotics in FPA production by 2030 [16].

Amongst 686 *Enterobacterales* isolated between 2000 and 2016, 107 (15.3%) organisms carrying *mcr* genes (*mcr*-1—97 *E*. *coli*, 3 *E*. *fergusonnii*, and 2 *Salmonella enterica* serovar Schwarzengrund; *mcr*-5—5 *E*. *coli*) on IncI2, IncHII, IncFIB, IncB/O, and IncX4 (predominantly) plasmids, as well as a class one integron, were observed [124,125,210,211,212,213,214]. This reveals that before the ban, MGEs and integrons ensured the considerably wide dissemination of *mcr*-1 among a diversity of *Enterobacterales* in the Brazilian poultry industry. It also shows that, similar to Asia [163], *E*. *fergusonii* is an emerging reservoir of *mcr* genes in South America [214]. Some *mcr*-1-positive *E*. *coli* were from chickens that received antimicrobial agents, mostly bacitracin, but not polymyxins [211], implying that bacitracin, which is a commonly used (in large quantities without the need for withdrawal and with poor gastrointestinal absorption) in-feed antimicrobial growth promoter/therapy, could exert selective pressure for the development of MGCB. Xu et al. [294] showed bacitracin to be a non-COL usage risk for COL resistance and *mcr*-1 acquisition. Thus, even after the ban on COL, the extensive use of other antimicrobials, including bacitracin, would have exerted selective pressure for the bacterial acquisition of *mcr* genes.

The *E*. *coli* isolates coexpressed *mcr*-1 with virulence genes and 12 other antimicrobial determinants, including ESBL and pAmpC (in isolates recovered in 2013) and *qac* genes, conferring resistance to five antimicrobial classes and heavy metals [124,125,210,211,212,213,214]. They were also extensively diversified, belonging to phylogroups A, B1, B2, C, D, and F, and five STs (Table 4), including HiR-ExPEC clone STThese findings suggest that organisms coproducing MCR-1 and ESBL/pAmpC have been present in Latin America for at least a decade and that potentially disinfectant/heavy-metal-resistant *mcr*-1-positive strains are circulating in the Brazilian poultry sector. However, *Salmonella* isolates exclusively harboured *mcr*-1 flanked by a hypothetical protein and *pap2* upstream and downstream, respectively [212], suggesting that an unknown genetic element might be involved in the acquisition/transfer of the *mcr*-1 gene in Latin America. This also supports that selection pressure from the use of non-polymyxin antimicrobials does not always confer *mcr* acquisition. Furthermore, some *mcr*-positive isolates exhibited susceptibility to COL (MIC 0.25–1 mg/L) [211], further confirming that by using the recommended epidemiological cut-off value (ECV) of >2 mg/L as a criterion for resistance [295], COL-susceptible *mcr*-carrying strains could go undetected, thereby favouring the dissemination of MGCB [210,211]. This warrants the revision of COL MIC breakpoints for *Enterobacterales*. 

Brazil is among the topmost chicken meat exporters in the world, with the highest domestic consumption, and is the most populous country in South America [124,292]. Thus, MGCB from the Brazilian poultry sector could easily spread within the population in Brazil and to other parts of the globe. Unfortunately, MGCB have disseminated into human–environmental sectors in Brazil [23,102,296].

#### 4.3.4. Southern South America

Argentina

Among 168 *E*. *coli* isolated between 2013 and 2014, 38 (22.6%) strains carrying *mcr*-1 on the IncI2 plasmid were detected [215,216]. IS*Apl1* bracketed *mcr*-1.5 upstream and downstream in the isolates, which also coexpressed ESBL, pAmpC, and PMQR genes (Table 4). They were also extensively diversified, belonging to eight STs (dominated by ST155) (Table 4), including HiR-ExPEC clones ST10 and ST410 [259]. Thus, IncI and IS*Apl1* have constituted the backbones for *mcr* and ESBL/pAmpC genes in commensal/virulent *E*. *coli* in the Argentine poultry industry for a decade at least. The isolates could transfer resistance genes to other organisms, having transferred COL and fluoroquinolone resistance to recipient organisms at a frequency of ~1.5 × 10^−3^. Unfortunately, MGCB have disseminated into the human–environmental ecosystem in Argentina [23,102,296]. The veterinary antibiotic use in Argentina is expected not to rise above 1.5% of the global use from 2017 to 2030 [16]. Notably, none of the 18 and 22 *E*. *coli* isolated in 2011 from chickens in other LMICs in Southern South America, such as Colombia and Venezuela, respectively, harboured the *mcr*-1 or *mcr*-2 gene [76].

#### 4.3.5. Central South America

##### Paraguay

A total of 29 *E*. *coli* harbouring *mcr*-5 on IncI (majorly), IncFII, and IncHI, among other plasmids, were recovered from 16 (24%) of 66 birds sampled in 2012 [48]. The isolates coexpressed *mcr*-5 with eight other resistance genes, including ESBL and pAmpC genes (Table 3), conferring resistance to four antimicrobial classes. These indicate that the Paraguayan poultry sector is a potential reservoir of MDR *mcr*-positive organisms. There was a Tn3-mediated insertion of *mcr*-5 with *bla*_CTX-M-8_ in the IncI/ST113 plasmid (associated with the cross-sectorial spread of *bla*_CTX-M-8_ in South America), the cocarriage of *mcr*-5 with *bla*_CMY-2_ in IncI/ST12 (an epidemic plasmid lineage carrying *bla*_CMY-2_), and the positive conjugation of *mcr*-5-associated plasmids, suggesting the coselection of broad-spectrum β-lactams and the possible movement of *mcr*-5 between different clinically important replicons, thus making it easily acquired and transferrable to other organisms. However, some isolates could not transfer *mcr*-5 due to truncated transposons in the F29:A-:B- plasmid. The isolates were extensively diversified, belonging to 14 STs, dominated by the ST457 clone, which is associated with the cross-sectorial dissemination of ESBL-encoding genes (Table 3), including HiR-ExPEC clone ST38 [259]. Thus, there is no clonal restriction in the dissemination of *mcr*-5 among potentially virulent and multidrug-resistant to extensively drug-resistant organisms in Paraguay. 

#### 4.3.6. North America

##### Dominican Republic 

Among 581 *Enterobacterales*, 3 (0.51%) MDR ExPEC isolates of ST410 that coexpressed *mcr*-1 (with IS*Apl1* upstream of *mcr*-1) and many other resistance genes conferring resistance to aminoglycosides, fluoroquinolones (*qnrB19* and *qnrS1*), antifolates, β-lactams, and amphenicols on the IncX4 plasmid were detected [76,126]. This suggests that despite the low *mcr* gene incidence, the poultry sector in the Dominican Republic is a potential source for the dissemination of virulent MGCB into the human and environmental settings, thereby posing a risk to individuals visiting the DR. Unfortunately, MGCB have been isolated from individuals who returned to the US from the DR [297].

#### 4.3.7. Europe 

Before 2013, 28.7% of COL produced in China ended up in Europe, where a sum of 545.2 tonnes of active polymyxin ingredients, including COL and polymyxin B, was used, primarily in the poultry and swine sectors, in 22 European countries [18]. Despite the fact that COL was recommended only for treating animal diseases (not for metaphylaxis in livestock) in 2013 in Europe, it continued to be used for the prophylactic control of intestinal diseases in livestock on the continent, with polymyxins ranking as the fifth most sold antimicrobial class in 2013 [232,234]. Following the discovery of the *mcr* gene in 2015, in 2016 in Europe, COL was placed in category “B” as a restricted drug whose use in veterinary medicine should be limited to reduce the danger to public health and only be used when there is no other alternative [298]. Although European member countries were asked to reduce the consumption of COL by 65% to attain below 5 mg/PCU by 2020, some countries exceeded the threshold in 2018, and COL consumption in human medicine in Europe increased from 2005 to 2018, probably due to an increase in MDR/XDR infections [232]. Thus, selection pressure for COL resistance is being exerted, despite the recently reported decreased COL consumption in some European countries [299]. In the poultry sector in European HICs, the incidence of *mcr*-bearing bacteria was reported to range between 0.7 and 57.1% [18]. Information on the occurrence of MGCB in LMICs in Europe is crucial to understanding the magnitude of the AMR problem and for the reevaluation of the strategies to control AMR. Four publications investigated MGCB among a total of 104 reported isolates from the poultry meat production chain in three LMICs in Europe [10] (Table 4). The *mcr*-1 gene was reported to be harboured by 24 organisms (23 *E*. *coli* and 1 *Salmonella* Enteritidis) among the tested isolates. 

##### Romania

Out of 96 COL-resistant *E*. *coli* isolated between 2011 and 2017, 18 (18.8%) strains carrying *mcr*-1 associated with the transposon Tn*6300* on IncX4, IncHI2, and IncI plasmids were detected [218,219]. The isolates were extensively diversified, belonging to five STs, including HiR-ExPEC clones ST10 and ST57, and coexpressed 15 additional resistance genes, including pAmpC, carbapenemase—*bla*_OXA-162_— and PMQR genes, conferring resistance to six antimicrobial classes [218,219] (Table 4). Interestingly, all Tn*6330*-positive isolates were of ST57 and had the same serotype (ST57:O86H25) and almost identical genotype as per the virulence, plasmid, and resistance genes analysed [218]. Thus, Tn*6300* integrated *mcr*-1 into the chromosome, thereby ensuring the clonal dissemination of the gene. Nevertheless, *mcr*-1 was stabilized due to the loss of IS*Apl1* in some Romanian isolates [218]. Like *E*. *coli* isolated from the Chinese poultry sector (B. T. Liu et al., 2017), *bla*_OXA-162_ collocated with *mcr*-1 on the IncHI2 plasmid, with *bla*_OXA-162_ uniquely associated with transposon Tn*6237* [219]. These findings suggest that 13 years ago at least, various MGEs evolved commensal/virulent multidrug- and COL-resistant *E*. *coli* clones that might be from various sources in the poultry sector in Eastern Europe. Romania has the third-highest human antibiotic consumption in Europe and is one of the EU member countries that consumed more than the recommended amount of COL [218,232]; thus, the selection pressure for the development of MGCB could be maintained in the livestock sector. 

##### Russia

The *mcr*-1.1 variant, bracketed by IS*26* and *pap2* upstream and downstream, was detected on the IncX4 plasmid in *Salmonella* Enteritidis isolated in 2019 [127], suggesting that *mcr*-1 has been circulating in Eastern Europe, perhaps at a low rate, since 2019 at least. It also supports that in *Salmonella*, the loss of IS*Apl1* also enables the anchorage of *mcr*-1 on the plasmid, thereby ensuring the stability of *mcr* on plasmids [156]. The isolate was exclusively resistant to COL, confirming that selective pressure for the development of MGCB is not necessarily imposed using other antimicrobials and that carriage of the *mcr* gene does not necessarily confer multiple-drug resistance [23]. Although the consumption of COL in veterinary medicine in Russia is negligible [300], its use, as well as other antimicrobials, possibly exerts selection pressure for the development of MGCB. Russia consumed 1.8% of the global veterinary antibiotics sold in 2017 [16]. Moreover, the use of COL in human medicine has increased in Russia due to the increasing incidence of carbapenem-resistant infections [300]. Lamentably, MGCB have diffused into human and environmental sectors, including wildlife, in this country [127,301]. Nonetheless, the Russian–Ukraine war and the economic sanctions on Russia could potentially cause the increased use of antimicrobials (projected to reach 1.9% by 2030 [16]), including COL, in Russia, thereby increasing the problem of AMR in Eastern Europe. 

##### Serbia

Only five *E*. *coli* carrying *mcr*-1 on the IncX4 plasmid were recovered from the Serbian poultry sector [220]. The isolates were diversified, belonging to HiR-ExPEC clones ST640, ST58, and ST410, and they harboured virulence genes, including markers of UPEC, APEC, and EHEC. They also coexpressed 13 other resistance genes (including PMQR genes) conferring resistance to six antimicrobial classes [220]. These findings suggest that virulent MDR *mcr*-1-harbouring *E*. *coli* are present in the Serbian poultry sector and that the IncX4 plasmid is a common backbone for *mcr*-1 in virulent *E*. *coli* clones in the Balkan peninsula. Though the use of antibiotics, including COL, as growth promoters in Serbia was officially banned in 2010, with a noticeable continuous decline in COL consumption [220], the use of non-polymyxin veterinary antimicrobials could be exerting selective pressure for COL resistance. Since Serbia is landlocked, MGCB could disseminate to and from neighbouring countries. Notably, four amongst seven non-*mcr*-harbouring COL-resistant *Salmonella* Infantis from the Serbian poultry sector had *pmrB* gene mutations [302]. 

## 5. Control Strategies against the Spread of *mcr* Gene-Containing Organisms in the Poultry Sector in LMICs 

Since poultry farmworkers potentially transport MGCB through their workwear (gloves, clothes, and boots), the restricted movement of persons and fomites and improved biosecurity measures, such as the periodical changing of disinfectant solutions in foot dips at poultry farm/pen entrances/exits, the cautious removal of workwear after work, and the regular disinfection and washing of poultry farm/slaughterhouse workers’ paraphernalia (clothes and boots), are critical in breaking the transmission cycle of MGCB. Screening poultry pens with nets and the prompt removal of dead bird carcasses prevent flies and mammalian vectors from introducing resistant organisms into the farms or transporting them out. To reduce the risk of the zoonotic transmission of MGCB from humans to animals and vice versa, the hand hygiene of poultry bird handlers and poultry feed handlers and their use of PPE should be improved following the WHO-recommended WASH (water, sanitation and hygiene) protocol [13]. Improved environmental sanitation and the timely removal of poultry litter could reduce the load of MGCB in the litter, thereby preventing the recolonization of birds by the organisms. To reduce the risk of acquiring MGCB from animal manure, the anaerobic digestion and composting of livestock manure before use as an organic fertilizer have been suggested [229]. The periodical disinfection of the ventilators in pens could reduce the airborne transmission of MGCB within poultry farms. The regular washing and periodical disinfection (especially after each batch of birds) of feeding and drinking troughs could prevent MGCB from entering the poultry chain through these pieces of equipment. 

As observed, banning the prophylactic use (and use as a growth enhancer) of COL in livestock reduces the rate of colonization of birds by MGCB. However, since the banning of prophylactic antimicrobial use in LMICs could potentially lead to decreased meat production and increased meat prices, with an inevitable increase in local poverty, country-specific antimicrobial supply chains and veterinary practices must be well understood before embarking on any legislation and trade control strategies [8]. In addition, the inappropriate therapeutic use of COL facilitates the acquisition and spread of the *mcr* gene. Thus, education for veterinarians and farmers about COL (regarding the mechanism of action, posology, and effects) is warranted to reduce the selection pressure resulting from COL underdosing. The improved uptake of vaccination reduces the use of antimicrobials for preventable diseases. Non-antibiotic agents such as probiotics, synbiotics, and antimicrobial peptides are potential effective alternatives to antimicrobial agents [30]. The judicious use of animal feed additives containing non-antibiotics such as bacitracin and non-polymyxin antibiotics, especially antimicrobials that are not used in humans and, crucially, do not select for resistance against human antibiotics, could also reduce the pressure for the selection of COL resistance in the exposed bacterial population [8,303]. The coselection of *mcr* and heavy-metal resistance could be reduced by the cautious use of heavy metals in livestock. Since the coselection of COL and TIG resistance is rapidly emerging in the poultry sectors of LMICs, the nontherapeutic use of tetracyclines in livestock should immediately be reconsidered [101]. Imperatively, a reduction in the use of all antimicrobial agents at the primary level of poultry production in LMICs is crucial to mitigate the effects of the complex mechanisms of coselection and multidrug resistance from “Consumer Protection” and “One Health” perspectives [304]. 

The periodical screening of poultry birds, including day-old chicks, poultry farms, slaughterhouses, hatchery trays, and workers for the presence of MGCB on their hands/rectum could help in early detection and isolation and the possible decolonization of individuals harbouring these organisms. A hazard analysis of MGCB at critical points in meat processing, storage, retailing, and shipping facilities would help in reducing the spread of MGCB through poultry meat handling, consumption, and trade. The prolonged cooking of meat (which may affect the nutritional quality) destroys pathogenic MGCB [55], thereby potentially preventing the acquisition of these organisms by meat consumers. Screening for *mcr* genes in isolates from poultry, irrespective of the group, and the COL MIC exhibited by the isolate is crucial in curtailing the silent dissemination of MGCB. WGS and metagenomics, which can detect the *mcr* gene in unculturable organisms [56], are indispensable tools for estimating the magnitude of the *mcr* gene in samples/ecological niches.

## 6. Future Perspectives and Conclusions

There is a need for more evidence regarding the direct impact of a ban on prophylactic COL use and the resistome. Screening archived isolates, especially those dating back to pre- and early COL-use periods, by WGS is crucial to understanding the present and future impacts of the ban. The dedicated screening of bacteria in the *Proteae* group (*Proteus*, *Providencia*, *Serratia*, and *Morganella*) to assess the incidence and ecology of *mcr* carriage among them is warranted. Disaggregating data based on the date of sampling is crucial for understanding the evolution and trends of MGCB over time in a specific geographical location. Monitoring resistance against other antibiotics in livestock farms and the formulation of effective control policies/strategies are crucial to preventing the acquisition of *mcr* and other resistance genes by bacterial organisms. The development of rapid and affordable methods that could identify known (or even yet unknown) bacterial species as well as currently known *mcr* genes, including *mcr* genes yet to emerge, is warranted. For an adequate understanding of *mcr* gene persistence in the environment even after COL withdrawal, *mcr*-bearing isolates should be examined for emerging polymorphic MGEs (that is, chromosomally integrated plasmids), which enables the bacterium to have a biphasic lifestyle that accelerates the bacterial response to adverse environments [244].

This review showed that a diversity of organisms, including Acinetobacter baumannii, *E. coli*, *E. fergusonii*, *Klebsiella*, *Salmonella*, *Cronobacter*, *Citrobacter*, *Raoultella*, *Enterobacter*, *Proteus*, *Shigella*, *Providencia*, *Aeromonas*, and *Pseudomonas* spp., harbouring various *mcr* genes are widely spread in the poultry industry in LMICs. *E*. *coli* is the predominant organism spreading the mcr gene in the poultry meat/egg supply chain in LMICs. The extensive use of COL and other antimicrobial and non-antimicrobial agents for promoting growth and the prophylactic control and metaphylactic treatment of intestinal infections is a factor stimulating the development of COL-resistant organisms and prompting the acquisition of the mcr gene in the poultry sector. The ban on nontherapeutic COL use is effective in reducing the development of MGCB. However, the ban on prophylactic COL use appears to have a limited influence on the prevalence of unrelated antibiotic resistance. Sources of MGCB in the poultry sector include breeder birds, hatcheries, poultry farmworkers, feed and drinking water contaminated by anthropogenic/agricultural wastes/handlers, animal vectors, contaminated fomites, and poultry litter. A deep litter system might be a risk factor for colonization by MGCB. Isolates of poultry origin in LMICs contain mcr with many virulence and resistance genes, including pAmpC, ESBL, carbapenemase, plasmid-mediated quinolone, fosfomycin, and tigecycline resistance genes (such as *tet*(X3), *tet*(X4), *tet*(X7) and *tmexCD1-toprJ1*), conferring resistance to last-resort antimicrobials. Thus, they are superbugs that can potentially cause difficult-to-treat disease outbreaks with pandemic potential in poultry farms and the human population. Since there is little or no access to effective last-resort antibiotics for human medicine in LMICs, the uncontrolled spread of superbugs in the poultry meat chain can potentially result in the outbreak of difficult-to-treat zoonotic foodborne infections. Some poultry isolates in LMICs have acquired megaplasmids with numerous ARGs (some harbouring ≥10 genes). The further transmission of these megaplasmid-containing organisms through farm-to-fork transmission may lead to the actualization of O’Neill’s projection of 10 million human AMR infection-associated deaths by 2050. 

Plasmids, including conjugative plasmids of different replicons and incompatibility, truncated and composite transposons (especially Tn*6330*), insertion sequences (especially IS*Apl1*), and integrons are drivers of *mcr* genes in the poultry sectors of LMICs. However, chromosomal mechanisms are also involved in COL resistance among isolates from poultry in these regions. The IncHI2, IncI2, and IncX4 plasmids seem to be the predominant plasmid types in strains of poultry origin in LMICs. These plasmids rapidly spread *mcr* genes to other organisms since they were transferred to recipient strains at high frequencies. Prophages also mediate the horizontal dissemination of *mcr* genes by transduction in the poultry sector. Nonetheless, *mcr*-1, *mcr*-3, and *mcr*-10 have integrated into chromosomal DNA and non-conjugative plasmids in poultry strains from LMICs, enabling vertical transfer to their progenies, thus ensuring the persistence of the *mcr* gene among clonal lineages. The persistence of the *mcr* gene in the environment could change the dynamics of AMR, and this is of serious public health concern. Transmission of the *mcr* gene among poultry strains in LMICs is non-clonal, and diverse, highly virulent zoonotic pandemic/epidemic and commensal clones of *E*. *coli*, *Klebsiella*, and *Salmonella* are circulating in the poultry industries in these regions. 

Contact with poultry birds, poultry manure, flies that feed on/breed in poultry manure, mammalian vectors in the poultry environment, and poultry farm workers/their workwear and equipment are potential routes for the acquisition of MGCB. The consumption/handling of undercooked or raw poultry meat and associated products is a putative route for colonization by MGCB. Poultry meats can be contaminated at the slaughterhouse, at packaging and/or selling/retail points by handlers (slaughterhouse personnel, meat sellers, or buyers/consumers) of these meats, and by flies in open-air markets found in LMICs. Integrated poultry–fish farms are also potential routes for the spread of MGCB from livestock to the human–aquaculture ecosystem in LMICs. The trade of poultry birds/meat and an associated product is a route for spreading MGCB from LMICs to other places. Insufficiently treated/untreated poultry litter/manure/sewage and slaughterhouse sewage/manure are potential sources for disseminating *mcr* genes into the human, soil, botanical, and aquatic/aquaculture environments, especially when they are used as organic fertilizer in aquaculture and farmlands. 

**Figure 3:**
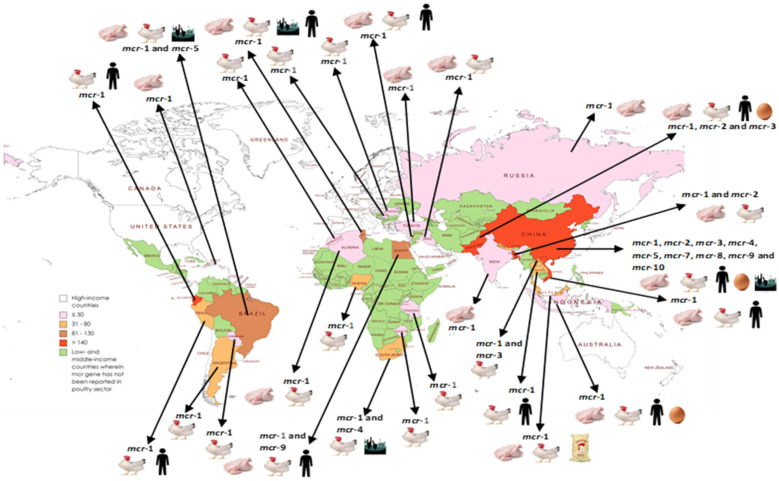
Distribution of mobile colistin resistance (*mcr*) gene-bearing organisms in poultry meat production and supply chains of low- and middle-income countries, 1970 to May 2023. 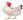: Poultry bird; 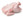: poultry meat; 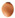: egg; 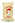: poultry feed; 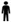: persons in contact with poultry bird/poultry product; 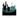: poultry environment (litter/manure, sewage, vectors, soil, or water). This map was created with https://www.mapchart.net/ (accessed on 30 May 2023).

Indeed, it is evident that *mcr*-1, *mcr*-2, *mcr*-3, *mcr*-4, *mcr*-5, *mcr*-7, *mcr*-8, *mcr*-9, and *mcr*-10 have disseminated in the poultry sector in LMICs (Figure 3). Thus, the poultry sector in LMICs is a huge (“phanthom resistomes”), underestimated reservoir of vast unseen determinants for last-resort antimicrobials. The farm-to-plate and farm-to-environmental transmission of superbugs from the poultry sector will increase if efforts to curtail the development and spread of *mcr*-gene-bearing organisms in the poultry meat supply chain in LMICs are not enhanced. This further highlights the need for the One Health approach.

## Figures and Tables

**Table 1:** Studies reporting on plasmid-mediated colistin resistance in isolates from poultry sector in Asian low- and middle-income countries.

Country	Source of Isolates	Date of Isolation (*mcr* Gene Assayed)	Number of Isolates Tested for *mcr*	Identified Gene (Number of Organisms)	Sequence Type	Virulence Genes (Phylogroup)	Plasmid (Associated Insertion Sequence and Integrons)	Additional Resistance Traits	References	
China	Poultry birds, meats, feed, organs, litter, sewage, bird market, and vectors in poultry environment	1970–2022 (*mcr*-1 to *mcr*-10)	16,288	*mcr*-1 (2400 *Escherichia coli*, 33 *Escherichia fergusonii*, 21 *Salmonella*, 9 *Klebsiella pneumoniae*, 3 *Citrobacter*, 2 *Cronobacter*, 1 *Enterobacter*, 1 *Rauoltella*, and 1 *Providencia*)	*E*. *coli*: ST6775, ST189, ST156, ST5454, ST602, ST362, ST117, ST2944, ST10338, ST1403, ST1421, ST162, ST3941, ST6321, ST69, ST7153, ST93, ST12735, ST297, ST2973, ST2847, ST101, ST617, ST10, ST1011, ST767, ST155, ST457, ST48, ST4204, ST533, ST1638, ST6395, ST4408, ST1968, ST83, ST21, ST696, ST226, ST3519, ST359, ST2179, ST124, ST6706, ST175, ST6740, ST65, ST1296, ST4935, ST612, ST171, ST7115, ST1158, ST2732, ST354, ST82, ST7189, ST616, ST88, ST77, ST542, ST5879, ST5865, ST1431, ST1290, ST873, ST971, ST952, ST5851, ST761, ST6050, ST351, ST361, ST744, ST3044, ST2491, ST2345, ST1642, ST5909, ST601, ST3944, ST870, ST3133, ST215, ST178, ST58, ST3481, ST5542, ST2914, ST68, ST501, ST38, ST2085, ST1060, ST827, ST131, ST2323, ST167, ST4214, ST302, ST540, ST227, ST3014, ST6257, ST410, ST452, ST3499, ST6484, ST95, ST2171, ST29, ST294, ST723, ST109, ST678, ST17, ST62, ST3, ST5694, ST127, ST2018, ST3489, ST6388, ST2736, ST206, ST2599, ST648, ST5498, ST1564, ST1589, ST3041, ST1286, ST5229, ST29, ST46, ST165, ST7157, ST220, ST16, ST7454, ST2929, ST2223, ST1582, ST5259, ST1251, ST219, ST4710, ST4477, ST423, ST224, ST168, ST43, ST4969, ST7584, ST8900, ST271, ST4753, ST1266, ST4129, ST7108, ST349, ST448, ST23, ST770, and ST22	*cvaC*, *etsC*, *hlyF*, *gad*, *ireA*, *iss*, *iuc*C, *iutA*, *terC*, *traT*, *Irea*, *iro*N, *aafII*, *eae*, *stx*1, *KpsM II*, *pap*A, *papC*, *fimD*, *pefC*, *mchF*, *ompT*, *sitA*, *entB,C,E,F*,*S*, *acrB*, *fepA*,*D,G*, *altB*, *ompA*, *gndA*, *galF*, *rpos*, *ugD*, *rfBK*, *gad*, *hra*, *lpfA*, *astA*, *gad*, *lpfA*, *cma*, *neuC*, *ompT* and *sitA* (A, B1, B2, and D)	IncI2, IncHI2, IncFIC, IncI, IncI1, IncFIA, IncFIB, IncFII, IncY, IncX2, IncX3, IncX4, IncHI2A, IncB/O/K/Z, IncP, IncN, Incp0111, IncA/C and IncL/M; and chromosomal (IS*AplI*, IS*Ecp1*, IS*CR1*, IS*Kpn3*, IS*As17*, IS26s, IS186B, IS*As13*, IS*Aeca6*, ∆IS*903B*, IS*As2*, IS*As20*, IS*Ecl1*, IS*Kpn26*, Δ1IS*Ahy2* and Δ2IS*Ahy2*, and *Intl*1	*tet*(X4), *tmexCD1*-*toprJ1, bla*_CTX-M-1_, *bla*_CTX-M-9_, *bla*_CTX-M-27_, *bla*_DHA-1_, *bla*_FOX-2_, *bla*_SHV-11_, *bla*_OXA-12_, *bla*_SHV_, *bla*_CEPH-A3-like_, *bla*_PSE_, *bla*_PER-3_, *bla*_CTX-M-14_, *bla*_CTX-M-15_, *bla*_CTX-M-28_, *bla*_SHV-28_, *bla*_CTX-M-44_, *bla*_TEM-99_, *bla*_CTX-M-3_, *bla*_CTX-M-55_, *bla*_CTX-M-64_, *bla*_CTX-M-137_, *bla*_CTX-M-65_, *bla*_CTX-M-66_, *bla*_CTX-M-82b_, *bla*_TEM-1A_, *bla*_TEM-1B_, *bla*_TEM-1D_, *bla*_CTX-M-27_, *bla*_SHV−73_, *bla*_TEM-141_, *bla*_TEM-122_, *bla*_MIR_, *bla*_CMY_, *bla*_CMY-2_, *ampC2*, *ampC1*, *bla*_NDM-1_, *bla*_NDM-4_, *bla*_TEM-198_, *bla*_OXA-1_, *bla*_OXA-10_, *bla*_NDM-5_, *erm*(B), *erm*(C), *erm*(42), *mef*(B), *mphI*, *lnu*(F), *rmtB, mdf*(A), *mph(A)*, *armA*, *aac(6)-Ia*, *aac(3′)-Iv*, *aacCA5*, *aadA*, *aac(6′)-Iaa*, *aac(6′)-Ib*, *aadA5*, *aadA7*, *aac(6ʹ)-Ib-cr*, *aadA16*, *aph(6)-Id*, *aadA1*, *aph(3′)-Ia*, *aph(3′)-Ib*, *aph(39)-IId*, *aph(300)-Ib*, *aph(39)-Ia*, *aac(3)-IId*, *aadA2*, *ant(6)-Ia*, *aac(3)-IIa*, *aac(3′)-IId*, *aph(4)-Ia*, *aphA2*, *ant(3′)-Ia*, *ant*(3″), *strA*, *strB*, *armA*, *sat2a*, *arr*, *tet*(A), *tet*(B), *tet*(C), *tet*(M), *tet*(D), I(E), *tet*(34), *te*t(O), *qnrA*, *qepA*, *qnrS*, *qnrD*, *qnrS1*, q*nrS4*, *qnrB4*, *qnrB52*, *oqxA*, *oqxB*, *fosA*, *fosA3*, *fosA7*, *floR*, *catA1*, *catB3*, *cml*, *cmlA*, *catB*, *cmlA1*, *ul1*, *sul2*, *sul3*, *dfrA1*, *dfrA5*, *dfrA7*, *dfrA10*, *dfrA12*, *dfrA14*, *dfrA17*, *dfrA27*, *qacL*, *qacE*, and mutations in *gyrA* and *parC*	[25,43,44,45,46,49,50,51,57,58,59,60,62,63,86,89,91,92,94,96,97,98,99,100,105,106,107,108,109,129,133,134,135,136,137,138,139,140,141,142,143,144,145,146,147,148,149,150,151,152,153,154,155,156,157,158,159,160,161,162,163,164,165,166,167,168,169,170,171]	
				*mcr*-2 (66 *E*. *coli*)						
				*mcr*-3 (5 *Aeromonas*, 1 *E*. *coli*, and 1 *Proteus*)						
				*mcr*-7 (3 *K. pneumoniae*)						
				*mcr*-8 (31 *K*. *pneumoniae* and 2 *Rauoltella*)						
				*mcr*-9 (131 *Salmonella*)						
				*mcr*-10 (2 *K*. *pneumoniae*, 2 *Enterobacter*, and 1 *E*. *coli*)						
	
					*Enterobacter*: ST1605 and ST1056					
					*K*. *pneumoniae*: ST11, ST15, ST37, ST3332, and ST395					
					*Salmonella*: ST292, ST399, ST34, and ST2529					
					*Aeromonas*: ST51, ST514, and ST216					
India	Poultry meat	2019 (*mcr*-1)	12	*mcr*-1 (2 *E*. *coli*)	ST50	*iss* and *gad*	IncX1 and IncHI2 (IS*Apl1*)	*aadA1*, *aadA2*, *aph(6)-Id*, *aph(3′)-Ib*,*bla*_TEM-1B_, *qnrS1*, *tetA*, *dfrA14*, *dfrA15*, *floR*, and *sul3*	[110]	
Pakistan	Chickens, chicken meat, internal organs, and secretions	2015–2020 (*mcr*-1 to *mcr*-10)	703	*mcr*-1 (228 *E*. *coli*, 12 *K*. *pneumoniae*, 1 *Pseudomonas aeruginosa*, and 1 *Proteus mirabilis*)	ST1035, ST131, ST1215, ST2279, ST88, ST1650, ST410, ST3059, ST10, ST95, ST2847, ST155, ST361, ST6395, ST244, ST23, ST7187, ST156, ST746 ST354, ST135, ST117, ST4085, ST761, ST744, ST58, ST1121, ST1267, ST221, ST11, ST392 ST6751, ST351, ST10009, ST38, ST617, ST694, ST3902, ST218, ST457, ST6635, ST2750, ST206, ST2690, ST1140, ST10010, ST8804, ST359, ST162, ST6635, ST93, ST4980, ST191, ST10011, ST1011, ST355, ST2224, ST2067, ST69, ST48, ST2253, ST2099, ST189, ST2207, ST2334, and ST6706	*ompT*, *hylF*, *iutA*, *iroN*, *iss*, *iucD*, *astA*, *tsh*, *papC*, and *cvi*	IncX4, IncF, IncY, p0111, IncH, IncI, IncQ, ColPVC, IncP, IncY, and so on (IS*CR1*, IS*Apl1*, and IS*ECP1*)	*tet*(X4), *bla*_CTX-M_, *bla*_CTX-M-15_, *bla*_TEM1B_, *bla*_TEM-1_, *bla*_SHV_, *bla*_CMY-2_, *bla*_NDM-1_, *bla*_KPC_, *bla*_OXA-48_, *bla*_IMP_, *tet*(A), *tet*(B), *sul1*, *floR*, *cmlA1*, *aadA1*, *strB*, *aac(6′)-Ib-cr*, *aph(3″)-Ib*, *aac(3)-IId*, *aph(3′)-Id*, *aph(3′)-Ia*, *aac(3)-IIa*, *aph(6′)-Ic*, *aph(6′)-Id*, *dfrA12*, *dfrA14*, *sul2*, *sul3*, and so on	[8,67,172,173,174,175,176,177,178,179,180]	
Bangladesh	Poultry birds, meats, farm environment, water, and cages	2017–2019 (*mcr*-1 to *mcr*-5)	3307	*mcr*-1 (471 *E*. *coli*, 17 *Proteus*, 10 *Klebsiella*, 12 *Salmonella*, 1 *Shigella*, and 2 *Enterobacter*)	ST1324, ST155, ST1818, ST354, ST178, ST43, ST4965, ST2705, ST1196, ST206, ST359, ST867, ST602, ST867, ST3107, ST48, and ST189	*iss*, *lpfA*, *eaeA*, *astA*, *etpD*, *air*, *eilA*, *cma, iroN*, *mchF*, *eilA*, *iha, ireA*, and *tssh*	IncHI1, IncHI, IncHI2, IncFIB, IncQ1, IncFIB, IncX1, and IncI2, IncN, IncFIA, ColRNAI, ColE10, ColpVC, and p0111 (IS*Apl1*)	*bla*_CTX-M-55_, *bla*_DHA-1_, *bla*_TEM-106_, *bla*_TEM-126_, *bla*_TEM-135_, *bla*_TEM-176_, *bla*_TEM-1A_, *bla*_TEM-1B_, *bla*_TEM-220_, *bla*_TEM-57_, *bla*_CTX-M-group-1_, *bla*_TEM_, *bla*_OXA-1_, *bla*_OXA-47_, *bla*_CTX-M-group-9_, *rmtB aac(3)-IId*, *aadA1*, *aadA2*, *aadA2b*, *aadA8b*,*aaph(3″)-Ib*,*’aph(3′)-Ia*, *aph(6)-Id*, *mdf*(A), *mph*(A), *catA1*, *cml*, *cmlA1*, *floR*, *qepA4*, *sul1*, *sul2*, *sul3*, *tet*(A), *tet*(B), *tet*(M), *dfrA1*, *dfrA12*, *dfrA14*, *dfrA17*, *qnrB2*, *qnrB4*, *qnrB7*, *qnrS*, *qnrB*. *qnrD3*, *qnrS1*, mutations in *gyrA*, *parC* *parE*, *pmrA*, and *pmrB*	[39,40,41,53,66,88,93,181,182,183]	
						*Salmonella*: *invA*				
				*mcr*-2 (2 *Proteus mirabilis*, 1 *E*. *coli*, 1 *K*. *pneumoniae*, 1 *Salmonella*, and 1 *Enterobacter*)						
				*mcr*-3 (11 *E*. *coli*)						
Nepal	Chickens and chicken vendors	2017–2019 (*mcr*-1 to *mcr*-10)	312	*mcr*-1 (68 *E*. *coli*)	ST23 and ST10	-	IncK/B and IncI2, IncI1, IncFIC(FII), and IncFIB	*bla*_OXA-48_, *bla*_CTX-M_, *tet*, *sul*, *qnr*, and *dfr*	[69,70,71]	
Lebanon	Chickens, chicken feed, litter, soil, and farmworkers	2017–2018 (*mcr*-1 to *mcr*-10)	617	*mcr*-1 (315 *E*. *coli* and 31 *K*. *pneumoniae*)	ST1011, ST6856, ST93, ST744, ST388, ST359, ST752, ST1421, ST6844, ST6115, ST354, ST1638, ST2705, ST48, ST206, ST398, ST1626, ST101, ST1140, ST226, ST2705, ST162, ST2936, ST3288, ST6448, ST746, ST1196, ST359, ST2220, ST5687, ST248, ST7458, ST115, ST1589, and ST3941	-	IncX4, IncI2, and IncHI2	*bla*_CMY-3_, *bla*_TEM-1B_, *bla*_TEM-1C_, *bla*_TEM-141_, *bla*_TEM_, *bla*_TEM-1_, *bla*_TEM-122_, *bla*_TEM-141_, *bla*_TEM-122_, *bla*_CTXM_, *bla*_CTXM-3_, *bla*_CTX-M-65_, *bla*_CTX-M-3_, *bla*_CTX-M-55_, *bla*_CMY-2_, *bla*_SHV_, *tet*(A), *tet*(B), *tet*(M), *tet*(), *qnrS1*, *gyr*A and *par*C, *sul1*, *sul2*, *sul3*, *dfrA15*, *dfrA17*, *dfrA1*, *dfrA14*, *dfrA17*, *dfrA5*, *floR*, *catA1*, *cmlA1*, *lnu*(F), *mdf*(A), *mph*(A), *mpH*(A), *fosA3*, *qnrS1*, aad, *aadA2*, *aadA5*, *aph(3′)-Ia*, *aph(6)-Id*, *aph(3″)-Ib*, *ant(3″)-Ia*, *aph(3′)-Iia*, *aac(3)-IId*, *aph(3′)-Ib*, *ant(3′)-Ia*, and *IntI1*	[35,47,184,185,186,187]	
Iraq	Chicken and turkey meats	2017–2019 (*mcr*-1)	200	*mcr*-1 (26 *Acinetobacter baumannii*)	-	*fimH*, *afa*/*draBC*, sfa/*foc DE*, *cnfI*, and *cnf2*	-	*bla*_CITM_, *bla*_IMP_, *bla*_SHV_, *bla*_SIM_, *bla*_VIM_, *bla*_OXA-58-like_, *bla*_OXA-24-like_, *bla*_OXA-23-like_, *bla*_OXA-51-like_, *aac(3)-I*, *aadA1*, *cmlA*, *cat1*, *sul1*, *dfrA1*, *tet*(B), and *tet*(A)	[121]	
Indonesia	Chickens, litter and drinking water in farms, and chicken meat	2017 (*mcr*-1)	58	*mcr*-1 (13 *E*. *coli*)	-	-	-	-	[55]	
Turkey	Chicken meat	2016–2019 (*mcr*-1 to *mcr*-10)	127	*mcr*-1 (5 *E*. *coli*)	ST3941, ST1049, and ST6094	*ast* and *gad*	IncX4 and IncI2	*bla*_TEM-1b_, *bla*_TEM-1c_, *bla*_CTX-M-8_, *qnrB19*, *mdf*(A), *tet*(A), *tet*(B), *sul1*, *sul2*, *dfrA1*, *floR*, *catA1*, *mphA*, *aph(3′)-Ib*, *aph(6)-Id*, and *aadA5*	[111,112]	
Lao Peoples Democratic Republic	Chickens and chicken meat	2018 (*mcr*-1 to *mcr*-8)	175	*mcr*-1 (34 *E*. *coli*)	ST165, ST10, ST1630, ST11090, ST2179, ST69, ST48, ST7352, ST212, ST48, ST206, ST165, ST46, ST117, ST5229, ST373, ST641, ST2690, ST648, and ST1585	*entC,E,B*, *fepA,B,D*, *fes*, *fimF*, *fimG*, *chuU,V,W*, *shuA* and *shuX* (A, B1, B2, C, D, E, F, G, and clade I)	IncX4, IncI2, IncP1, IncFII, IncFIA, IncFIB, IncR and IncHI1 (IS*15DI*,) IS*Kpn40*, and *IS26*	*bla*_TEM-1_, *bla*_CTX-M-55_, *bla*_CTX-M-123_, *aac(3)-IId*, *aadA1*, *aadA2*, *aadA2b*, *aph(300)-Ib*, *aph(6)-Id*, *aac(3)-IV*, *aac(6′)-Ib-cr*, *aph(4)-Ia*, *aph(6)-Id*, *aph(3″)-Ib*,’*aph(3′)-Ia*, *mdf*(A), *mph*(A), *cmlA1*, *catB3*, *floR*, *sul2*, *sul3*, *dfrA12*, *dfrA1*, *tet*(A), *tet*(M), *fosA3*, *arr-3*, *qnrS1*, *oqxA*, *oqxB*, and *qnrS13*	[87,122]	
				*mcr*-3 (3 *E*. *coli*)						
Thailand	Chickens and ducks	2013–2019 (*mcr*-1)	34	*mcr*-1 *(*75 *E*. *coli* and 1 *Salmonella)*	ST2973	-	-	*bla*_CTXM-14_	[83,130]	
Malaysia	Chickens, chicken meat, litter and feed	2013–2021 (*mcr*-1 to *mcr*-5)	262	*mcr*-1 (63 *E*. *coli* and 1 *Salmonella enterica*)	ST3489, ST93, ST540, ST69, ST154, ST345, ST196, ST1001, ST1638, ST155, ST2179, ST872, ST410, ST373, ST770, and ST117	A, B1, B2, and D	IncI2, IncHI1A, IncHI1B IncQ1, IncFIA, IncFIB, IncI1, IncI2, IncFIC, and ColpVc (IS*Apl1*)	*aadA1*, *aadA2*, *bla*_TEM-1B_, *bla*_CTX-M-55_, *bla*_TEM-52,_*bla*_NDM_, *bla*_OXA-48_, *bla*_IMP_, *qnrS1*, *fosA*, *mph(A)*, *mef(B)*, *floR*, *sul1*, *sul3*, *tet*(A), *tet*(M), *tet*(34), *dfrA12*, *aph(3′)-Ia*, *aac-3-IV*, *aadA1*, *aac(6)-lb*, *strA*, and *strB*	[54,68,120,188,189,190,191]	
				*mcr-5* (1 *Salmonella*)						
Vietnam	Chickens and chicken meat	2011–2019 (*mcr*-1 to *mcr*-10)	872	*mcr*-1 (278 *E*. *coli*)	ST156, ST50, ST162, ST155, ST93, ST 354, ST1158, ST2690, ST48, ST206, ST69, ST6726, ST648, ST656, and ST1602	*astA*, *csgG*, *entA,B,C,D,E,F,S*, *fepA,B*,C*,D,G*, *fes*, *ompA*, *aslA*, *csgB,D,F*, *fdeC*, *fimA,B,C,D,E,F,G,H,I*, *gspC,E,F,G,H,I,J,K,L,M*, *yagV/ecpE*, *yagW/ecpD*, *yagX/ecpC*, *yagY/ecpB*, *yagZ/ecpA*, *ykgk/ecpR*, *astA*, *chuS,T,U,V,W*, *chuY*, *fyuA*, *iroN*, *iutA*, *kpsD*, *kpsM*, and *papC*	IncHI2, IncI2, IncHI2A IncN, IncX4, IncY, and IncP-1 (IS*AplI*)	*bla*_TEM_, *bla*_CTX-M_, *bla*_CTX-M-2_, *bla*_CTX-M-65_, *bla*_CTX-M-9_, *bla*_CTX-M-1_, *bla*_OXA-1_, *bla*_CMY-2_, *bla*_CARB-3_, *bla*_CARB-2_, *bla*_CTX-M_, *ampC*, *bla*_OXA_, *bla*_TEM−1_, *tet*(A), *strA*, *strB*, *aac(3)-IIa*, *aac(6′)-IIa*, *ant(3″)-IIa*, *aph(3″)-Ib*, *aph(3′)-Ia*, *aph(6)-Id*, *aadA*, *fosA*, *sul2*, *sul3*, *dfrA*, *floR*, *catA4*, *catB3*, *emr*(E), *mphA*, *mphB*, *qnrD1*, *qnrS, qnrA*, and mutation in *gyrA,*	[38,84,113,114,115,116,117,118,119]	

**Table 2:** Studies reporting on plasmid-mediated colistin resistance in isolates from poultry sector in African low- and middle-income countries.

Country	Source of Isolate	Date of Isolation (*mcr* Gene Assayed)	Number of Isolates Tested for *mcr*	Identified Gene (Number of Organisms)	Sequence Type	Virulence Genes (Phylogroup)	Plasmid (Associated Insertion Sequence)	Additional Resistance Traits	References
Tunisia	Chickens and chicken meat	2015–2018 (*mcr*-1to *mcr*-10)	195	*mcr*-1 (116 *E*. *coli*)	ST398, ST4187, ST2197, ST10, ST69, ST349, ST57, ST1011, ST3882, ST5693, ST8932, ST162, ST2220, ST5686, ST57, ST117, and ST6798	*fimA*, *stx1*, *stx2*, *papC* and *aer* (A, B1, B2, D, and D2)	IncI1, IncI2, IncHI2, IncFIB, and IncP (IS*Apl1*)	*bla*_TEM_, *bla*_SHV_, *bla*_CMY-2_, *bla*_CTX-M-1_, *bla*_CTX-M-g-1_, *bla*_CTX-M-55_, *bla*_CTX-M-14_, *bla*_CMY_, *bla*_TEM-1b,_*bla*_CTX-M-1_, *tet*(A), *tet*(B), *aadA*, *aadA1 strA*, *strB*, *sul1*, *sul3*, *dfrA*, *floR*, *cmlA*, and *Int*1	[72,73,74,75,191,192,193]
Algeria	Chickens and chicken meat	2015–2017 (*mcr*-1 to *mcr*-9)	27	*mcr*-1 (16 *E*. *coli*)	ST48, ST758, ST224, ST168, ST1241, ST260, ST 5161, ST155, ST10, ST744, ST 4654, and ST648	*fimH*, *iro*N, *iutA*, *iucD*, *traT*, *cva*, *iss*, *ompT*, *sfa*, *foc*, *afa*, *dr*, *kpsMTII*, *papA*, *papC*, *hlyA*, *ireA* and *malX* (A, B1, and F)	IncHI2, IncFV, and IncFIIK (IS*Apl1*)	*bla*_TEM-1_, *bla*_SHV-1_, *sul1*, *sul2*, and *sul*	[123,190,194]
Egypt	Chickens, chicken farm workers, environment, and meats	2010–2020 (*mcr*-1 to *mcr*-10)	448	*mcr*-1 (111 *E*. *coli* and 1 *Citrobacter freundii*)	ST986, ST373, ST156, ST1011, ST5687, ST1125, ST371, ST398, ST196, ST101, ST115, and ST10	*cma*, *hemL*, *iroN*, *iss*, *pic*, *vat*, *hylE*, *ireA*, *mchF sitABCD* operon, and *iucABCD*/*iutA* operon	IncHI2, IncI2, and IncX4 (IS*Apl1* and Tn*6330*)	*tet*(X7), *bla*_TEM_, *bla*_TEM-1_, *bla*_CTXM_, *bla*_CTX-M-9_, *bla*_SHV-12_, *bla*_CTX-M-15_, *bla*_CTX-M-14_, *bla*_TEM-1B_, *bla*_CTX-M-1_, *bla*_OXA-1_, *bla*_CMY_, *bla*_NDM_, *bla*_VIM_, *bla*_SHV_, *sul1*, *sul2*, *sul3*, *dfrA1*, *dfrA14*, *dfrA7*, *dfrA12*, *dfrA15*, *fosA4*, *tet*(A), *aadA1*, *aphA*, *mphA*, *mrx*, *aadA2*, *aph(6)-Id*, *aac(3)- IIa*, *aph(39′)-Ib*, *aph(39)-Ia*, *strA*, *strB*, *lnu(F)*, *ere*(A), *erm*(B), *erm*(42), *mph*(A), *mph*(B), *mdfA*, *catA1*, *floR*, *cmlA1*, and *arr-2*	[76,77,78,79,80,195,196,197]
				*mcr*-9 (1 *E*. *coli*)					
Morocco	Chickens	2012–2017 (*mcr*-1)	12	*mcr*-1 (3 *E*. *coli*)	-	-	-	-	[81]
South Africa	Chickens and rats in poultry farms	2015-2019 (*mcr*-1 to *mcr*-5)	115	*mcr*-1 (39 *E*. *coli*)	-	-	IncI2 (IS*Apl1*)	*bla*_CTX-M-1_, *bla*_TEM_, *bla*_CTX-M-2_, *bla*_CTX-M-9_, *bla*_CTX-M-15_, *aadA1*, *strA*, *strB*, *tetA*, *sul3*, *dfrA12*, *IntI*1, and *IntI*2	[82,198,199]
				*mcr*-4 (31 *Salmonella*)					
Zimbabwe	Chickens and ducks	2017–2019 (*mcr*-1 to *mcr*-10)	21	*mcr*-1 (1 *E*. *coli*)	ST10	*fimH*, *csg*, *agn43*, *kpsD*, *kpsMll*, *ibeBC*, *fes*, *fepA*, *iucA*, *gspL*, and *hlyE*	IncF	*qnr*, *tet*, bla_TEM_, *bla*_OXA_, and *bla*_CTX-M_	[200]
Nigeria	Chickens, chicken farmers, chicken farm water, and live bird market environment	2018–2020 (*mcr*-1 to *mcr*-10)	450	*mcr*-1 (32 *E*. *coli*, 2 *K*. *pneumoniae*, and 1 *Citrobacter werkmanii*)	ST34, ST48, ST155, ST1286, ST226, ST10, ST656, ST4542, ST168, ST398, ST6836, ST746, and ST2485	-	IncX4	*bla*_TEM-93_, *bla*_TEM-57_, *bla*_CTX-M-55_, *bla*_CMY-47_, *bla*_TEM-1_, *tet*(G), *tet*(D), *tet*(C), *qnrB17*, *qnrB19*, *qnrS1*, *mdfA*, *aph(3)-Ib*, *aph(3)-Ia*, *aph(6)-Id*, *aadA1*, *aac(3)-IId*, *sul2*, and *catA1*	[34,201,202]
				*mcr*-5 (1 *E*. *coli*)					
				*mcr*-8 (1 *E*. *coli* and 1 *K*. *pneumoniae*)					

*mcr*, Mobile colistin resistance gene; -, no data; Additional resistance traits, resistance factors identified in one *mcr*-positive isolate or pooled factors in more than one *mcr*-bearing isolate; Virulence genes, genes from *mcr*-bearing *E*. *coli* isolates; Sequence type, Warwick multilocus sequence type of *mcr*-bearing *E*. *coli* isolates unless otherwise stated; Plasmid, plasmid types identified in one or pooled *mcr*-bearing isolates; Inc., incompatibility.

**Table 3:** Studies reporting on plasmid-mediated colistin resistance in isolates from poultry sector in South American low- and middle-income countries.

Country	Source of Isolate	Date of Isolation (*mcr* Gene Assayed)	Number of Isolates Tested for *mcr*	Identified Gene/Variant (Number of Organisms)	Sequence Type	Virulence Genes (Phylogroup)	Plasmid (Associated Insertion Sequence)	Additional Resistance Traits	References
Ecuador	Chickens	2013–2020 (*mcr*-1)	326	*mcr*-1 (145 *E*. *coli*)	ST394, ST5855, ST6995, ST6940, ST10, ST98, and ST3856	-	IncI	*bla*_CTX-M-2_, *bla*_CTX-M-14_, *bla*_CTX-M-65_, *bla*_CMY-2_, *bla*_OXA-48_, *bla*_CTXM-1_, *bla*_TEM_, *bla*_CTXM-9_, *bla*_NDM_, *fosA3*, *aadA5*, *dfrA17*, *sul1*, *tet*(B), and mutations in *gyrA* and *parC*	[203,204,205,206,207]
Peru	Chickens	2018–2020 (*mcr*-1 to *mcr*-10)	274	*mcr*-1 (44 *E*. *coli*)	ST23 ST10, ST48, ST602, ST746, ST46, and ST345	*gad*, *iss*, *astA*, *cba*, *cma*, *iha*, *iroN*, *lpfA*, *capU*, *kpsE*, *kpsMII_K5*, *ompT*, *sepA*, *traT*, *cea*, *cib*, *hlyF*, *stb*, *toxB*, *cif*, *espABFJ*, *nleABC*, *terC*, *tir*, *eae*, *perA*, *tsh*, *fyuA*, *ireA*, *irp2*, *iucC*, *iutA*, *sitA*, and *hlyF*	IncFIB, IncI1, IncFIC, IncX1, ColRNAI, and IncFII	*bla*_CTX-M-65_, *bla*_TEM-30_, *aadA1*, *aadA2*, *aph(4)-Ia*, *aac(3)-Iva*, *aac(3)-IIa*, *aac(3)-IId*, *aadA5*, *aadA15*, *aadA17*, *aph(3′)-Ia*, *aph(3′)-IIa*, *aph(6)-Id*, *mph*(A), *catA*, *floR*, *cmlA1*, *sul2*, *sul3*, *dfrA17*, *tet*(B), *tet*(A), *dfrA1*, and mutations in *gyrA* and *parC*	[208,209]
Brazil	Chickens and poultry meats	2000–2016 (*mcr*-1to *mcr*-4)	686	*mcr*-1 (97 *E*. *coli*, 3 *E*. *fergusonnii* and 2 *Salmonella enterica* serovar Schwarzengrund)	ST132, ST48, ST4419, ST96, ST522, and ST10	*iss*, *iroN*, *lpfA*, *mchB*, *mchC*, *mchF*, *hlyF*, *ompT*, *iss*, *iron* and *iutA*, *ireA*, and *gad* (A, B1, B2, and D)	IncX4, IncI2, IncHII, IncFIB, and IncB/O (IS*Ec12*)	*bla*_TEM_, *bla*_CTX-M-1_, *bla*_CTX-M-8_, *bla*_CTX-M-15_, *bla*_CTX-M-group 2_, *bla*_SHV_, *bla*_CMY-2_, *bl*a_CTX-M-8_, *aadA, aadA1*, *aadB*, *aac(3)-VI*, *aadA2*, *aadA5*, *aph(3′)-Ic*, *aph(3)-IA*, *sul1*, *sul2*, *dfr17*, *tet*(A), *tet*(B), *pco*D, *qac*, and *IntI*1	[124,125,210,211,212,213,214]
				*mcr*-5 (5 *E*. *coli*)					
Argentina	Chickens	2013–2014 (*mcr*-1)	168	*mcr*-1 (38 *E*. *coli*)	ST617, ST1141, ST410, ST155, ST1286, ST1011, ST10 and ST1408	-	IncI2 (IS*Apl1*)	*bla*_CTX-M-14_, *bla*_CMY-2_, *bla*_CTX-M-2_, *bla*_CMY-2_, *qnrA*, *qnrB*, *qnrD*, *qnrS*, *oqxB*, and *qepA*	[215,216]
Paraguay	Chickens	2012 (*mcr*-1 to *mcr*-5)	66	*mcr*-5 (29 *E*. *coli*)	ST457, ST38, ST57, ST8061, ST224, ST580, ST6853, ST189, ST93, and ST2705	F	IncI1, IncFII, IncHI1, IncI1-N, and IncFII-FIB	*bla*_CTX-M-8_, *bla*_CMY-2_, *bla*_SHV-12_, *bla*_TEM-1A_, *aph(6)-Id*, *aph(3″)-Ib*, *aph(3″)-Ib*, *sul2*, *mdf*(A), and *dfrA8*	[48,217]

*mcr*, Mobile colistin resistance gene; additional resistance traits, resistance factors identified in one *mcr*-positive isolate or pooled factors in more than one *mcr*-bearing isolate; virulence genes, genes from *mcr*-bearing *E*. *coli* isolates unless otherwise stated; sequence type, Warwick multilocus sequence type of *mcr*-bearing *E*. *coli* isolates; plasmid, plasmid types identified in one or pooled *mcr*-bearing isolates; Inc., incompatibility.

**Table 4:** Studies reporting on plasmid-mediated colistin resistance in isolates from poultry sector in European low- and middle-income countries.

Country	Source of Isolate	Date of Isolation (*mcr* Gene Assayed)	Number of Isolates Tested for *mcr*	Identified Gene (Number of Organisms)	Sequence Type	Virulence Genes (Phylogroup)	Plasmid (Associated Insertion Sequence)	Additional Resistance Traits	References
Romania	Chickens	2011–2017 (*mcr*-1 to *mcr*-10)	96	*mcr*-1 (18 *E*. *coli*)	ST744, ST57, ST156, ST10, and ST4980	*astA, gad, iss, ompT*, and *terC* (A, B1 and D)	IncHI2, IncX, IncF, and IncI (IS*Apl1* and Tn*6330*)	*bla*_TEM-1B,_*bla*_TEM-1_, *bla*_CMY_, *bla*_OXA-62_, *acc(3)-IIa*,*aph(3)-Ia*, *aph(3″)-Ib*, *aph(6)-Id*,*strA*, *strB*, *sul2*, *sul3*,*tet*(A), *floR*, and mutations in *gyrA* and *par*C	[218,219]
Russia	Chicken meat	2019 (*mcr*-1)	3	*mcr*-1 (1 *Salmonella* Enteritidis)	ST11	-	IncX4	-	[127]
Serbia	Turkeys	2020 (*mcr*-1 to *mcr*-5)	5	*mcr*-1 (5 *E*. *coli*)	ST410, ST641, and ST58	*cfaA-H*, *ecpA-E*, *elfA,C,D,H*, *hpcA-C*, *fimA,C-I*, *eaeH*, *aec15-32*, f*liC*, *sitA-D*, *iroB-E,N*, *fyuA*, *irp1/2*, *ybtA,E,P,Q,S-U,X*, *cah*, *ehaA*/*B/G*, *upaG*, *agn43*, *espL1,L4,R1,X1,X4*,*X5*, *ibeB/C*, *hlyE/clyA*, *uge*, *wzc*, *flaA*, *ompT*, and *rmlD*	IncX4	*bla*_TEM-1_, *aadA1*, *aadA2*, *strA*, *strB*, *sul2*, *sul3*, *dfrA12*, *qnrS1*, *tet*(A), *tet*(M), *cmlA1*, *floR*, and mutations in *gyrA* and *parC*	[220]

*mcr*, Mobile colistin resistance gene; -, no data; additional resistance traits, resistance factors identified in one *mcr*-positive isolate or pooled factors in more than one *mcr*-bearing isolate; virulence genes, genes from *mcr*-bearing *E*. *coli* isolates unless otherwise stated; sequence type, Warwick multilocus sequence type of *mcr*-bearing *E*. *coli* isolates; plasmid, plasmid types identified in one or pooled *mcr*-bearing isolates; Inc., incompatibility.

## Data Availability

Not applicable.

## Supplementary Materials

The following supporting information can be downloaded at https://www.mdpi.com/article/10.3390/antibiotics12071117/s1. Table S1: Studies reporting on plasmid-mediated colistin resistance in the poultry meat supply chain in China.

## Abbreviations

COL—colistin; AMR—antimicrobial resistance; LMICs—low- and middle-income countries; MGE—mobile genetic element; SDG—sustainable development goal; MDR—multidrug-resistant; XDR—extensively drug-resistant; PDR—pandrug-resistant; HGT—horizontal gene transfer; GNB—Gram-negative bacilli; CIA—critically important antibiotic; HP-CIA—highest-priority critically important antibiotic; ESBL—extended-spectrum beta-lactamase; pAMpC—plasmidic ampicillinase C; PMQR—plasmid-mediated quinolone resistance; ESKAPE—*Enterobacter*, *Staphylococcus*, *Klebsiella*, *Acinetobacter*, *Pseudomonas aeruginosa* and *Enterococcus*; FPAs—food-producing animals; MGCB—*mcr* gene-containing bacteria; DOC—day-old chicks; ST—sequence type; TIG—tigecycline; APEC—avian pathogenic *Escherichia coli*; HiR-ExPEC—high-risk extraintestinal pathogenic *Escherichia coli*; MENA—Middle East and North Africa; WASH—Water, sanitation and hygiene.

## References

[1] Gajdács M., Urbán E., Stájer A., Baráth Z. (2021). Antimicrobial Resistance in the Context of the Sustainable Development Goals: A Brief Review. Eur. J. Investig. Health Psychol. Educ..

[2] Murray C.J., Ikuta K.S., Sharara F., Swetschinski L., Robles Aguilar G., Gray A., Han C., Bisignano C., Rao P., Wool E. (2022). Global Burden of Bacterial Antimicrobial Resistance in 2019: A Systematic Analysis. Lancet.

[3] O’Neill J. Antimicrobial Resistance: Tackling a Crisis for the Health and Wealth of Nations. Rev. Antimicrob. Resist..

[4] Erian I., Phillips C.J.C. (2017). Public Understanding and Attitudes towards Meat Chicken Production and Relations to Consumption. Animals.

[5] Li W., Bai X., Sheng H., Chen J., Wang Z., Wang T., Sun R., Feng Z., Wang Y., Peng K. (2022). Conjugative Transfer of Mcr-1-Bearing Plasmid from Salmonella to *Escherichia coli* in Vitro on Chicken Meat and in Mouse Gut. Food Res. Int..

[6] Mottet A., Tempio G. (2017). Global Poultry Production: Current State and Future Outlook and Challenges. Worlds Poult. Sci. J..

[7] De Mesquita Souza Saraiva M., Lim K., do Monte D.F.M., Givisiez P.E.N., Alves L.B.R., de Freitas Neto O.C., Kariuki S., Júnior A.B., de Oliveira C.J.B., Gebreyes W.A. (2022). Antimicrobial Resistance in the Globalized Food Chain: A One Health Perspective Applied to the Poultry Industry. Braz. J. Microbiol..

[8] Umair M., Hassan B., Farzana R., Ali Q., Sands K., Mathias J., Afegbua S., Haque M.N., Walsh T.R., Mohsin M. (2023). International Manufacturing and Trade in Colistin, Its Implications in Colistin Resistance and One Health Global Policies: A Microbiological, Economic, and Anthropological Study. Lancet Microbe.

[9] Cuong N.V., Padungtod P., Thwaites G., Carrique-Mas J.J. (2018). Antimicrobial Usage in Animal Production: A Review of the Literature with a Focus on Low-and Middle-Income Countries. Antibiotics.

[10] The World Bank World Bank Country and Lending Groups. https://datahelpdesk.worldbank.org/knowledgebase/articles/906519-world-bank-country-and-lending-groups.

[11] Anyanwu M.U., Jaja I.F., Oguttu J.W., Jaja C.J., Chah K.F., Shodeinde Shoyinka V. (2021). Is Africa Ready for Mobile Colistin Resistance Threat?. Infect. Ecol. Epidemiol..

[12] Njoga E.O., Ogugua A.J., Nwankwo I.O., Awoyomi O.J., Okoli C.E., Buba D.M., Oyeleye F.A., Ajibo F.E., Azor N., Ogunniran T.M. (2021). Antimicrobial Drug Usage Pattern in Poultry Farms in Nigeria: Implications for Food Safety, Public Health and Poultry Disease Management. Vet. Ital..

[13] Ikhimiukor O.O., Odih E.E., Donado-Godoy P., Okeke I.N. (2022). A Bottom-up View of Antimicrobial Resistance Transmission in Developing Countries. Nat. Microbiol..

[14] Njoga E.O., Onunkwo J.I., Okoli C.E., Ugwuoke W.I., Nwanta J.A., Chah K.F. (2018). Assessment of Antimicrobial Drug Administration and Antimicrobial Residues in Food Animals in Enugu State, Nigeria. Trop. Anim. Health Prod..

[15] Coyne L., Arief R., Benigno C., Giang V.N., Huong L.Q., Jeamsripong S., Kalpravidh W., McGrane J., Padungtod P., Patrick I. (2019). Characterizing Antimicrobial Use in the Livestock Sector in Three South East Asian Countries (Indonesia, Thailand, and Vietnam). Antibiotics.

[16] Tiseo K., Huber L., Gilbert M., Robinson T.P., Van Boeckel T.P. (2020). Global Trends in Antimicrobial Use in Food Animals from 2017 to 2030. Antibiotics.

[17] Wang X., Zhang H., Long X., Xu X., Ren H., Mao D., Alvarez P.J.J., Luo Y. (2023). Global Increase of Antibiotic Resistance Genes in Conjugative Plasmids. Microbiol. Spectr..

[18] Anyanwu M.U., Jaja I.F., Okpala C.O.R., Jaja C.J.I., Oguttu J.W., Chah K.F., Shoyinka V.S. (2021). Potential Sources and Characteristic Occurrence of Mobile Colistin Resistance (Mcr) Gene-Harbouring Bacteria Recovered from the Poultry Sector: A Literature Synthesis Specific to High-Income Countries. PeerJ.

[19] Shen Y., Zhang R., Schwarz S., Wu C., Shen J., Walsh T.R., Wang Y. (2020). Farm Animals and Aquaculture: Significant Reservoirs of Mobile Colistin Resistance Genes. Environ. Microbiol..

[20] Mulchandani R., Wang Y., Gilbert M., Van Boeckel T.P. (2023). Global trends in antimicrobial use in food-producing animals: 2020 to 2030. PLOS Global Public Health.

[21] Hamame A., Davoust B., Cherak Z., Rolain J.M., Diene S.M. (2022). Mobile Colistin Resistance (Mcr) Genes in Cats and Dogs and Their Zoonotic Transmission Risks. Pathogens.

[22] Abavisani M., Bostanghadiri N., Ghahramanpour H., Kodori M., Akrami F., Fathizadeh H., Hashemi A., Rastegari-Pouyani M. (2023). Colistin Resistance Mechanisms in Gram-Negative Bacteria: A Focus on *Escherichia coli*. Lett. Appl. Microbiol..

[23] Anyanwu M.U., Jaja I.F., Nwobi O.C. (2020). Occurrence and Characteristics of Mobile Colistin Resistance (Mcr) Gene-Containing Isolates from the Environment: A Review. Int. J. Environ. Res. Public Health.

[24] Anyanwu M.U., Anyogu D.C., Chah K.F., Shoyinka V.S. (2022). Mobile Colistin Resistance (Mcr-1) Gene-Positive *Escherichia coli* from Chickens in Nigeria Is Potentially Pathogenic and Transfers Colistin Resistance to Other Organisms. Comp. Clin. Path.

[25] Liu Y.-Y., Wang Y., Walsh T.R., Yi L.-X., Zhang R., Spencer J., Doi Y., Tian G., Dong B., Huang X. (2016). Emergence of Plasmid-Mediated Colistin Resistance Mechanism MCR-1 in Animals and Human Beings in China: A Microbiological and Molecular Biological Study. Lancet Infect. Dis..

[26] Anyanwu M.U., Jaja I.F., Nwobi O.C., Mgbeahuruike A.C., Ikpendu C.N., Okafor N.A., Oguttu J.W. (2022). Epidemiology and Traits of Mobile Colistin Resistance (Mcr) Gene-Bearing Organisms from Horses. Microorganisms.

[27] Martiny H.-M., Munk P., Brinch C., Szarvas J., Aarestrup F.M., Petersen T.N. (2022). Global Distribution of Mcr Gene Variants in 214K Metagenomic Samples. mSystems.

[28] Xiao X., Zeng F., Li R., Liu Y., Wang Z. (2022). Subinhibitory Concentration of Colistin Promotes the Conjugation Frequencies of Mcr-1- and Bla NDM-5 -Positive Plasmids. Microbiol. Spectr..

[29] He K., Li W., Zhao B., Xu H., Pan Y., He D., Hu G., Wu H., Yuan L. (2022). Spreading Advantages of Coresident Plasmids Bla CTX-M-Bearing IncFII and Mcr-1-Bearing IncI2 in *Escherichia coli*. Antimicrob. Chemother..

[30] Anyanwu M.U., Okpala C.O.R., Chah K.F., Shoyinka V.S. (2021). Prevalence and Traits of Mobile Colistin Resistance Gene Harbouring Isolates from Different Ecosystems in Africa. Biomed. Res. Int..

[31] Van T.T.H., Yidana Z., Smooker P.M., Coloe P.J. (2020). Antibiotic Use in Food Animals Worldwide, with a Focus on Africa: Pluses and Minuses. J. Glob. Antimicrob. Resist..

[32] Mead A., Richez P., Azzariti S., Pelligand L. (2021). Pharmacokinetics of Colistin in the Gastrointestinal Tract of Poultry Following Dosing via Drinking Water and Its Bactericidal Impact on Enteric *Escherichia coli*. Front. Vet. Sci..

[33] Sharafi T., Ardebili A. (2019). Plastic Binding Feature of Polymyxins: The Effect on MIC Susceptibility Measurements. Infect. Drug Resist..

[34] Anyanwu M.U., Marrollo R., Paolucci M., Brovarone F., Nardini P., Chah K.F., Shoyinka S.V.O., Carretto E. (2021). Isolation and Characterisation of Colistin-Resistant Enterobacterales from Chickens in Southeast Nigeria. J. Glob. Antimicrob. Resist..

[35] Sia C.M., Greig D.R., Day M., Hartman H., Painset A., Doumith M., Meunier D., Jenkins C., Chattaway M.A., Hopkins K.L. (2020). The characterization of mobile colistin resistance (*mcr*) genes among 33000 *Salmonella enterica* genomes from routine public health surveillance in England. Microb. Genom..

[36] Apostolakos I., Piccirillo A. (2018). A Review on the Current Situation and Challenges of Colistin Resistance in Poultry Production. Avian Pathol..

[37] Coppola N., Cordeiro N.F., Trenchi G., Esposito F., Fuga B., Fuentes-Castillo D., Lincopan N., Iriarte A., Bado I., Vignoli R. (2022). Imported One-Day-Old Chicks as Trojan Horses for Multidrug-Resistant Priority Pathogens Harboring Mcr-9, RmtG, and Extended-Spectrum β-Lactamase Genes. Appl. Environ. Microbiol..

[38] Nguyen N.T., Nguyen H.M., Nguyen C.V., Nguyen T.v., Nguyen M.T., Thai H.Q., Ho M.H., Thwaites G., Ngo H.T., Baker S. (2016). Use of Colistin and Other Critical Antimicrobials on Pig and Chicken Farms in Southern Vietnam and Its Association with Resistance in Commensal *Escherichia coli* Bacteria. Appl. Environ. Microbiol..

[39] Ahmed S., Das T., Islam M.Z., Herrero-Fresno A., Biswas P.K., Olsen J.E. (2020). High Prevalence of Mcr-1-Encoded Colistin Resistance in Commensal *Escherichia coli* from Broiler Chicken in Bangladesh. Sci. Rep..

[40] Das T., Islam M.Z., Rana E.A., Dutta A., Ahmed S., Barua H., Biswas P.K. (2021). Abundance of Mobilized Colistin Resistance Gene (Mcr-1) in Commensal *Escherichia coli* from Diverse Sources. Microb. Drug Resist..

[41] Jalal M.S., Dutta A., Das T., Islam M.Z. (2020). First Detection of Plasmid-Mediated Colistin-Resistance Gene (Mcr-1, Mcr-2 and Mcr-3) in *Escherichia coli* Isolated from Breeder Poultry of Bangladesh. Int. J. Infect. Dis..

[42] Gantois I., Ducatelle R., Pasmans F., Haesebrouck F., Gast R., Humphrey T.J., Van Immerseel F. (2009). Mechanisms of Egg Contamination by Salmonella Enteritidis: Review Article. FEMS Microbiol. Rev..

[43] Hu Y., Fanning S., Nguyen S.V., Wang W., Liu C., Cui X., Dong Y., Gan X., Xu J., Li F. (2021). Emergence of a Salmonella Enterica Serovar Typhimurium ST34 Isolate, CFSA629, Carrying a Novel Mcr-1.19 Variant Cultured from Egg in China. J. Antimicrob. Chemother..

[44] Li C., Gu X., Zhang L., Liu Y., Li Y., Zou M., Liu B. (2022). The Occurrence and Genomic Characteristics of Mcr-1-Harboring Salmonella from Retail Meats and Eggs in Qingdao, China. Foods.

[45] Wang Y., Zhang R., Li J., Wu Z., Yin W., Schwarz S., Tyrrell J.M., Zheng Y., Wang S., Shen Z. (2017). Comprehensive Resistome Analysis Reveals the Prevalence of NDM and MCR-1 in Chinese Poultry Production. Nat. Microbiol..

[46] Zhao X., Liu Z., Zhang Y., Yuan X., Hu M., Liu Y. (2020). Prevalence and Molecular Characteristics of Avian-Origin Mcr-1-Harboring *Escherichia coli* in Shandong Province, China. Front. Microbiol..

[47] Al-Mir H., Osman M., Drapeau A., Hamze M., Madec J.Y., Haenni M. (2021). WGS Analysis of Clonal and Plasmidic Epidemiology of Colistin-Resistance Mediated by Mcr Genes in the Poultry Sector in Lebanon. Front. Microbiol..

[48] Nesporova K., Jamborova I., Valcek A., Medvecky M., Literak I., Dolejska M. (2019). Various Conjugative Plasmids Carrying the Mcr-5 Gene in *Escherichia coli* Isolates from Healthy Chickens in Paraguay. J. Antimicrob. Chemother..

[49] Wang R., Liu Y., Zhang Q., Jin L., Wang Q., Zhang Y., Wang X., Hu M., Li L., Qi J. (2018). The Prevalence of Colistin Resistance in *Escherichia coli* and *Klebsiella pneumoniae* Isolated from Food Animals in China: Coexistence of Mcr-1 and BlaNDM with Low Fitness Cost. Int. J. Antimicrob. Agents.

[50] Shen Y., Lv Z., Yang L., Liu D., Ou Y., Xu C., Liu W., Yuan D., Hao Y., He J. (2019). Integrated Aquaculture Contributes to the Transfer of Mcr-1 between Animals and Humans via the Aquaculture Supply Chain. Environ. Int..

[51] Yang X., Peng K., Zhang Y., Liu L., Li R. (2020). Characterization of a Novel Mcr-8.2-Bearing Plasmid in ST395 *Klebsiella pneumoniae* of Chicken Origin. Infect. Drug Resist..

[52] Yang Y.Q., Li Y.X., Lei C.W., Zhang A.Y., Wang H.N. (2018). Novel Plasmid-Mediated Colistin Resistance Gene Mcr-7.1 in *Klebsiella pneumoniae*. J. Antimicrob. Chemother..

[53] Ahmed S., Das T., Islam M., Herrero-Fresno A., Biswas P., Olsen J. (2020). Abundance of Mobilized Colistin Resistance Gene Mcr-1 in Genetically Diverse Commensal *Escherichia coli* in Broiler Chicken in Bangladesh. Int. J. Infect. Dis..

[54] Yu C.Y., Ang G.Y., Chin P.S., Ngeow Y.F., Yin W.F., Chan K.G. (2016). Emergence of Mcr-1-Mediated Colistin Resistance in *Escherichia coli* in Malaysia. Int. J. Antimicrob. Agents.

[55] Palupi M.F., Wibawan I.W.T., Sudarnika E., Maheshwari H., Darusman H.S. (2019). Prevalence of Mcr-1 Colistin Resistance Gene in *Escherichia coli* along 19 Broiler Meat Supply Chain in Indonesia. Biotropia.

[56] Bich V.T.N., Thanh L.V., Thai P.D., Van Phuong T.T., Oomen M., Driessen C., Beuken E., Hoang T.H., Van Doorn H.R., Penders J. (2019). An Exploration of the Gut and Environmental Resistome in a Community in Northern Vietnam in Relation to Antibiotic Use. Antimicrob. Resist. Infect. Control.

[57] Zhang J., Chen L., Wang J., Yassin A.K., Butaye P., Kelly P., Gong J., Guo W., Li J., Li M. (2018). Molecular Detection of Colistin Resistance Genes (Mcr-1, Mcr-2 and Mcr-3) in Nasal/Oropharyngeal and Anal/Cloacal Swabs from Pigs and Poultry. Sci. Rep..

[58] Shi X., Li Y., Yang Y., Shen Z., Cai C., Wang Y., Walsh T.R., Shen J., Wu Y., Wang S. (2021). High Prevalence and Persistence of Carbapenem and Colistin Resistance in Livestock Farm Environments in China. J. Hazard. Mater..

[59] Ding S., Han X., Li J., Gao W., Chen Z., Feng Y. (2018). Discovery of Multi-Drug Resistant, MCR-1 and ESBL-Coproducing ST117 *Escherichia coli* from Diseased Chickens in Northeast China. Sci. Bull..

[60] Liu X., Li R., Dong N., Ye L., Chan E.W.-C., Chen S. (2021). Complete Genetic Analysis of Plasmids Carried by Two Nonclonal Bla NDM-5—And Mcr-1 -Bearing *Escherichia coli* Strains: Insight into Plasmid Transmission among Foodborne Bacteria. Microbiol. Spectr..

[61] Song Y., Yu L., Zhang Y., Dai Y., Wang P., Feng C., Liu M., Sun S., Xie Z., Wang F. (2020). Prevalence and Characteristics of Multidrug-Resistant Mcr-1-Positive *Escherichia coli* Isolates from Broiler Chickens in Tai’an, China. Poult. Sci..

[62] Wang X., Liu Y., Qi X., Wang R., Jin L., Zhao M., Zhang Y., Wang Q., Chen H., Wang H. (2017). Molecular Epidemiology of Colistin-Resistant Enterobacteriaceae in Inpatient and Avian Isolates from China: High Prevalence of Mcr-Negative *Klebsiella pneumoniae*. Int. J. Antimicrob. Agents.

[63] Zhuge X., Ji Y., Tang F., Sun Y., Jiang M., Hu W., Wu Y., Xue F., Ren J., Zhu W. (2019). Population Structure and Antimicrobial Resistance Traits of Avian-Origin Mcr-1-Positive *Escherichia coli* in Eastern China, 2015 to 2017. Transbound. Emerg. Dis..

[64] Xiang Y., Liu Z., Yu G., Song Y., Li Y., Geng X., Ma L., Guo J., Tan L., Chen P. (2023). Genetic characteristic of coexisting of mcr-1 and blaNDM-5 in Escherichia coli isolates from lesion bearing animal organs. Front. Microbiol..

[65] Amin M.B., Sraboni A.S., Hossain M.I., Roy S., Mozmader T.A.U., Unicomb L., Rousham E.K., Islam M.A. (2020). Occurrence and Genetic Characteristics of Mcr-1-Positive Colistin-Resistant *E. coli* from Poultry Environments in Bangladesh. J. Glob. Antimicrob. Resist..

[66] Dutta A., Barua H., Jalal M.S., Dhar P.K., Biswas S.K., Biswas P.K. (2018). An Investigation of Plasmid-Mediated Colistin Resistance Mechanism, MCR in *Escherichia coli* of Human, Veterinary and Environmental Origin in Bangladesh. Int. Journal. Infect. Dis..

[67] Usman M., Rasool M.H., Khurshid M., Aslam B., Baloch Z. (2023). Co-Occurrence of Mcr-1 and Carbapenem Resistance in Avian Pathogenic *E. coli* Serogroup O78 ST95 from Cololibacillosis-Infected Broiler Chickens. Antibiotics.

[68] Petrillo M., Angers-Loustau A., Kreysa J. (2016). Possible Genetic Events Producing Colistin Resistance Gene *mcr*-1. Lancet Infect. Dis..

[69] Joshi P.R., Thummeepak R., Paudel S., Acharya M., Pradhan S., Banjara M.R., Leungtongkam U., Sitthisak S. (2019). Molecular Characterization of Colistin-Resistant *Escherichia coli* Isolated from Chickens: First Report from Nepal. Microb. Drug Resist..

[70] Bista S., Shrestha U.T., Dhungel B., Koirala P., Gompo T.R., Shrestha N., Adhikari N., Joshi D.R., Banjara M.R., Adhikari B. (2020). Detection of Plasmid-Mediated Colistin Resistant Mcr-1 Gene in *Escherichia coli* Isolated from Infected Chicken Livers in Nepal. Animals.

[71] Muktan B., Thapa Shrestha U., Dhungel B., Mishra B.C., Shrestha N., Adhikari N., Banjara M.R., Adhikari B., Rijal K.R., Ghimire P. (2020). Plasmid Mediated Colistin Resistant Mcr-1 and Co-Existence of OXA-48 among *Escherichia coli* from Clinical and Poultry Isolates: First Report from Nepal. Gut Pathog..

[72] Dhaouadi S., Soufi L., Hamza A., Fedida D., Zied C., Awadhi E., Mtibaa M., Hassen B., Cherif A., Torres C. (2020). Co-Occurrence of Mcr-1 Mediated Colistin Resistance and β-Lactamase-Encoding Genes in Multidrug-Resistant *Escherichia coli* from Broiler Chickens with Colibacillosis in Tunisia. J. Glob. Antimicrob. Resist..

[73] Grami R., Mansour W., Mehri W., Bouallègue O., Boujaâfar N., Madec J., Haenni M. (2016). Impact of Food Animal Trade on the Spread of Mcr-1-Mediated Colistin Resistance, Tunisia, July. Eurosurveillance.

[74] Hassen B., Abbassi M.S., Ruiz-Ripa L., Mama O.M., Hassen A., Torres C., Hammami S. (2020). High Prevalence of Mcr-1 Encoding Colistin Resistance and First Identification of BlaCTX-M-55 in ESBL/CMY-2-Producing *Escherichia coli* Isolated from Chicken Faeces and Retail Meat in Tunisia. Int. J. Food Microbiol..

[75] Saidani M., Messadi L., Chaouechi A., Tabib I., Saras E., Soudani A., Daaloul-Jedidi M., Mamlouk A., ben Chehida F., Chakroun C. (2019). High Genetic Diversity of Enterobacteriaceae Clones and Plasmids Disseminating Resistance to Extended-Spectrum Cephalosporins and Colistin in Healthy Chicken in Tunisia. Microb. Drug Resist..

[76] Lima Barbieri N., Nielsen D.W., Wannemuehler Y., Cavender T., Hussein A., Yan S., Nolan L.K., Logue C.M. (2017). Mcr-1 Identified in Avian Pathogenic *Escherichia coli* (APEC). PLoS ONE.

[77] Elmonir W., Abd El-Aziz N.K., Tartor Y.H., Moustafa S.M., Abo Remela E.M., Eissa R., Saad H.A., Tawab A.A. (2021). Emergence of Colistin and Carbapenem Resistance in Extended-Spectrum β-Lactamase Producing *Klebsiella pneumoniae* Isolated from Chickens and Humans in Egypt. Biology.

[78] Sadek M., Ortiz de la Rosa J.M., Abdelfattah Maky M., Korashe Dandrawy M., Nordmann P., Poirel L. (2021). Genomic Features of MCR-1 and Extended-Spectrum β-Lactamase-Producing Enterobacterales from Retail Raw Chicken in Egypt. Microorganisms.

[79] Soliman A.M., Ramadan H., Zarad H., Sugawara Y., Yu L., Sugai M., Shimamoto T., Hiott L.M., Frye J.G., Jackson C.R. (2021). Coproduction of Tet(X7) Conferring High-Level Tigecycline Resistance, Fosfomycin FosA4, and Colistin Mcr-1.1 in *Escherichia coli* Strains from Chickens in Egypt. Antimicrob. Agents Chemother..

[80] Badr H., Samir A., El-Tokhi E.I., Shahein M.A., Rady F.M., Hakim A.S., Fouad E.A., El-Sady E.F., Ali S.F. (2022). Phenotypic and Genotypic Screening of Colistin Resistance Associated with Emerging Pathogenic Escherichia coli Isolated from Poultry. Vet. Sci..

[81] Rahmatallah N., El Rhaffouli H., Laraqui A., Sekhsokh Y., Lahlou-Amine I., El Houadfi M., Fihri O.F. (2018). Detection of Colistin Encoding Resistance Genes MCR-1 in Isolates Recovered from Broiler Chickens in Morocco. Saudi J. Pathol. Microbiol..

[82] Ramatla T., Mileng K., Ndou R., Mphuti N., Syakalima M., Lekota K.E., Thekisoe O.M.M. (2022). Molecular Detection of Integrons, Colistin and β-Lactamase Resistant Genes in Salmonella Enterica Serovars Enteritidis and Typhimurium Isolated from Chickens and Rats Inhabiting Poultry Farms. Microorganisms.

[83] Sudatip D., Mostacci N., Tiengrim S., Thamlikitkul V., Chasiri K., Kritiyakan A., Phanprasit W., Thinphovong C., Abdallah R., Baron S.A. (2023). The Risk of Pig and Chicken Farming for Carriage and Transmission of *Escherichia coli* Containing Extended-Spectrum Beta-Lactamase (ESBL) and Mobile Colistin Resistance (Mcr) Genes in Thailand. Microb. Genom..

[84] Nguyen P.T.L., Hung T.T.M., Anh T.H., Thai P.D., Tan L.M., Thanh N.H., Lien N.T.P., Tho N.T.T., Ha H.T.A., Nguyen C. (2022). Carriage of Plasmid-Mediated Colistin Resistance-1-Positive *Escherichia coli* in Humans, Animals, and Environment on Farms in Vietnam. Am. J. Trop. Med. Hyg..

[85] Wang X., Wang Y., Wang Y., Zhang S., Shen Z., Wang S. (2018). Emergence of the Colistin Resistance Gene Mcr-1 and Its Variant in Several Uncommon Species of Enterobacteriaceae from Commercial Poultry Farm Surrounding Environments. Vet. Microbiol..

[86] Wang X., Wang Y., Zhou Y., Wang Z., Wang Y., Zhang S., Shen Z. (2019). Emergence of Colistin Resistance Gene Mcr-8 and Its Variant in *Raoultella ornithinolytica*. Front. Microbiol..

[87] Zhou Y., Farzana R., Sihalath S., Rattanavong S., Vongsouvath M., Mayxay M., Sands K., Newton P.N., Dance D.A.B., Hassan B. (2022). A One-Health Sampling Strategy to Explore the Dissemination and Relationship between Colistin Resistance in Human, Animal, and Environmental Sectors in Laos. Engineering.

[88] Sobur A.M., Ievy S., Haque Z.F., Nahar A., Zaman S.B., Rahman M.T. (2019). Emergence of Colistin-Resistant *Escherichia coli* in Poultry, House Flies, and Pond Water in Mymensingh, Bangladesh. J. Adv. Vet. Anim. Res..

[89] Hu Z., Chen X., Wang Z., Guo G., Xu Z., Zhou Q., Wei X., Liu Y., Zhou L., Tan Z. (2022). Whole-Genome Analyses of APEC Carrying Mcr-1 in Some Coastal Areas of China from 2019 to 2020. J. Glob. Antimicrob. Resist..

[90] Lu X., Xiao X., Liu Y., Li Y., Li R., Wang Z. (2019). Chromosome-Mediated Mcr-1 in *Escherichia coli* Strain L73 from a Goose. Int. J. Antimicrob. Agents.

[91] Lu X., Du Y., Peng K., Zhang W., Li J., Wang Z., Li R. (2022). Coexistence of Tet (X4), Mcr-1, and Bla NDM-5 in ST6775 *Escherichia coli* Isolates of Animal Origin in China. Microbiol. Spectr..

[92] Lu X., Zhang W., Mohsin M., Wang M., Li J., Wang Z., Li R. (2023). The Prevalence of Plasmid-Mediated Colistin Resistance Gene *Mcr-1* and Different Transferability and Fitness of *Mcr-1* -Bearing IncX4 Plasmids in *Escherichia coli* from Pigeons. Microbiol. Spectr..

[93] Islam S., Urmi U.L., Rana M., Sultana F., Jahan N., Hossain B., Iqbal S., Hossain M.M., Mosaddek A.S.M., Nahar S. (2020). High Abundance of the Colistin Resistance Gene Mcr-1 in Chicken Gut-Bacteria in Bangladesh. Sci. Rep..

[94] Hu J., Yang J., Chen W., Liu Z., Zhao Q., Yang H., Sun Z., Chen X., Li J. (2022). Prevalence and Characteristics of Mcr-1-Producing *Escherichia coli* in Three Kinds of Poultry in Changsha, China. Front. Microbiol..

[95] Yassin A.K., Zhang J., Wang J., Chen L., Kelly P., Butaye P., Lu G., Gong J., Li M., Wei L. (2017). Identification and Characterization of Mcr Mediated Colistin Resistance in Extraintestinal *Escherichia coli* from Poultry and Livestock in China. FEMS Microbiol. Lett..

[96] Hu Y., Nguyen S.V., Liu C., Wang W., Dong Y., Fanning S., Li F. (2019). Complete Genome and Plasmid Sequences of Seven Isolates of *Salmonella enterica* Subsp. Enterica Harboring the Mcr-1 Gene Obtained from Food in China. Microbiol. Resour. Announc..

[97] Li X.P., Fang L.X., Song J.Q., Xia J., Huo W., Fang J.T., Liao X.P., Liu Y.H., Feng Y., Sun J. (2016). Clonal Spread of Mcr-1 in PMQR-Carrying ST34 Salmonella Isolates from Animals in China. Sci. Rep..

[98] Lyu N., Feng Y., Pan Y., Huang H., Liu Y., Xue C., Zhu B., Hu Y. (2021). Genomic Characterization of Salmonella Enterica Isolates from Retail Meat in Beijing, China. Front. Microbiol..

[99] Sheng H., Ma J., Yang Q., Li W., Zhang Q., Feng C., Chen J., Qin M., Su X., Wang P. (2022). Prevalence and Characteristics of Mcr-9-Positive Salmonella Isolated from Retail Food in China. LWT.

[100] Tang B., Chang J., Zhang L., Liu L., Xia X., Hassan B.H., Jia X., Yang H., Feng Y. (2020). Carriage of Distinct Mcr-1-Harboring Plasmids by Unusual Serotypes of *Salmonella*. Adv. Biosyst..

[101] Anyanwu M.U., Nwobi O.C., Okpala C.O.R., Ezeonu I.M. (2022). Mobile Tigecycline Resistance: An Emerging Health Catastrophe Requiring Urgent One Health Global Intervention. Front. Microbiol..

[102] Oliveira C.C., Lopes E.S., Barbosa D.R., Pimenta R.L., Sobrinho N.M.B.A., Coelho S.M.O., Souza M.M.S., Coelho I.S. (2019). Occurrence of the Colistin Resistance Mcr-1 Gene in Soils from Intensive Vegetable Production and Native Vegetation. Eur. J. Soil. Sci..

[103] He Y., Yuan Q., Mathieu J., Stadler L., Senehi N., Sun R., Alvarez P.J.J. (2020). Antibiotic Resistance Genes from Livestock Waste: Occurrence, Dissemination, and Treatment. NPJ Clean Water.

[104] Lv Z., Shen Y., Liu W., Ye H., Liu D., Liu J., Fu Y., Peng C., Chen K., Deng X. (2022). Prevalence and Risk Factors of Mcr-1-Positive Volunteers after Colistin Banning as Animal Growth Promoter in China: A Community-Based Case–Control Study. Clin. Microbiol. Infect..

[105] Bai S., Yu Y., Kuang X., Li X., Wang M., Sun R., Sun J., Liu Y., Liao X. (2022). Molecular Characteristics of Antimicrobial Resistance and Virulence in *Klebsiella pneumoniae* Strains Isolated from Goose Farms in Hainan, China. Appl. Environ. Microbiol..

[106] Cao Y.P., Lin Q.Q., He W.Y., Wang J., Yi M.Y., Lv L.C., Yang J., Liu J.H., Guo J.Y. (2020). Co-Selection May Explain the Unexpectedly High Prevalence of Plasmid-Mediated Colistin Resistance Gene Mcr-1 in a Chinese Broiler Farm. Zool. Res..

[107] Huang X., Yu L., Chen X., Zhi C., Yao X., Liu Y., Wu S., Guo Z., Yi L., Zeng Z. (2017). High Prevalence of Colistin Resistance and *mcr-1* Gene in *Escherichia coli* Isolated from Food Animals in China. Front. Microbiol..

[108] Shafiq M., Huang J., Shah J.M., Ali I., Rahman S.U., Wang L. (2021). Characterization and Resistant Determinants Linked to Mobile Elements of ESBL-Producing and Mcr-1-Positive *Escherichia coli* Recovered from the Chicken Origin. Microb. Pathog..

[109] Wang J., Tang B., Lin R., Zheng X., Ma J., Xiong X., Jiang H., Yang H., Ding B. (2022). Emergence of Mcr-1- and BlaNDM-5-Harbouring IncHI2 Plasmids in *Escherichia coli* Strains Isolated from Meat in Zhejiang, China. J. Glob. Antimicrob. Resist..

[110] Ghafur A., Shankar C., GnanaSoundari P., Venkatesan M., Mani D., Thirunarayanan M.A., Veeraraghavan B. (2019). Detection of Chromosomal and Plasmid-Mediated Mechanisms of Colistin Resistance in *Escherichia coli* and *Klebsiella pneumoniae* from Indian Food Samples. J. Glob. Antimicrob. Resist..

[111] Kurekci C., Aydin M., Nalbantoglu O.U., Gundogdu A. (2018). First Report of *Escherichia coli* Carrying the Mobile Colistin Resistance Gene Mcr-1 in Turkey. J. Glob. Antimicrob. Resist..

[112] Adiguzel M.C., Baran A., Wu Z., Cengiz S., Dai L., Oz C., Ozmenli E., Goulart D.B., Sahin O. (2021). Prevalence of Colistin Resistance in *Escherichia coli* in Eastern Turkey and Genomic Characterization of an Mcr-1 Positive Strain from Retail Chicken Meat. Microb. Drug Resist..

[113] Le P.Q., Awasthi S.P., Hatanaka N., Hinenoya A., Hassan J., Ombarak R.A., Iguchi A., Tran N.T.T., Dao K.V.T., Vien M.Q. (2021). Prevalence of Mobile Colistin Resistance (Mcr) Genes in Extended-Spectrum β-Lactamase-Producing *Escherichia coli* Isolated from Retail Raw Foods in Nha Trang, Vietnam. Int. J. Food Microbiol..

[114] Malhotra-Kumar S., Xavier B.B., Das A.J., Lammens C., Hoang H.T.T., Pham N.T., Goossens H. (2016). Colistin-Resistant *Escherichia coli* Harbouring Mcr-1 Isolated from Food Animals in Hanoi, Vietnam. Lancet Infect. Dis..

[115] Nakayama T., le Thi H., Thanh P.N., Minh D.T.N., Hoang O.N., Hoai P.H., Yamaguchi T., Jinnai M., Do P.N., Van C.D. (2022). Abundance of Colistin-Resistant *Escherichia coli* Harbouring Mcr-1 and Extended-Spectrum β-Lactamase-Producing *E. coli* Co-Harbouring Bla CTX-M-55 or -65 with Bla TEM Isolates from Chicken Meat in Vietnam. Arch. Microbiol..

[116] Nguyen T.N., Khong D.T., Le H.v., Tran H.T., Phan Q.N., Le H.T.T., Kawahara R., Yamamoto Y. (2021). Quantitative Analysis of Colistin-Resistant *Escherichia coli* in Retail Meat from Local Vietnamese Markets. Biomed. Res. Int..

[117] Trung N.V., Matamoros S., Carrique-Mas J.J., Nghia N.H., Nhung N.T., Chieu T.T.B., Mai H.H., van Rooijen W., Campbell J., Wagenaar J.A. (2017). Zoonotic Transmission of Mcr-1 Colistin Resistance Gene from Small-Scale Poultry Farms, Vietnam. Emerg. Infect. Dis..

[118] Vounba P., Rhouma M., Arsenault J., Bada Alambédji R., Fravalo P., Fairbrother J.M. (2019). Prevalence of Colistin Resistance and Mcr-1/Mcr-2 Genes in Extended-Spectrum β-Lactamase/AmpC-Producing *Escherichia coli* Isolated from Chickens in Canada, Senegal and Vietnam. J. Glob. Antimicrob. Resist..

[119] Yamaguchi T., Kawahara R., Harada K., Teruya S., Nakayama T., Motooka D., Nakamura S., do Nguyen P., Kumeda Y., van Dang C. (2018). The Presence of Colistin Resistance Gene Mcr-1 and -3 in ESBL Producing *Escherichia coli* Isolated from Food in Ho Chi Minh City, Vietnam. FEMS Microbiol. Lett..

[120] Aklilu E., Raman K. (2020). MCR-1 Gene Encoded Colistin-Resistant *Escherichia coli* in Raw Chicken Meat and Bean Sprouts in Malaysia. Int. J. Microbiol..

[121] Ghaffoori Kanaan M.H., Al-Shadeedi S.M.J., Al-Massody A.J., Ghasemian A. (2020). Drug Resistance and Virulence Traits of Acinetobacter Baumannii from Turkey and Chicken Raw Meat. Comp. Immunol. Microbiol. Infect. Dis..

[122] Moser A.I., Kuenzli E., Campos-Madueno E.I., Büdel T., Rattanavong S., Vongsouvath M., Hatz C., Endimiani A. (2021). Antimicrobial-Resistant *Escherichia coli* Strains and Their Plasmids in People, Poultry, and Chicken Meat in Laos. Front. Microbiol..

[123] Chaalal N., Touati A., Yahiaoui-Martinez A., Aissa M.A., Sotto A., Lavigne J.P., Pantel A. (2021). Colistin-Resistant Enterobacterales Isolated from Chicken Meat in Western Algeria. Microb. Drug Resist..

[124] Cyoia P.S., Koga V.L., Nishio E.K., Houle S., Dozois C.M., de Brito K.C.T., de Brito B.G., Nakazato G., Kobayashi R.K.T. (2019). Distribution of ExPEC Virulence Factors, BlaCTX-M, FosA3, and Mcr-1 in *Escherichia coli* isolated from Commercialized Chicken Carcasses. Front. Microbiol..

[125] Monte D.F., Mem A., Fernandes M.R., Cerdeira L., Esposito F., Galvão J.A., Franco B.D.G.M., Lincopan N., Landgraf M. (2017). Chicken Meat as a Reservoir of Colistin-Resistant *Escherichia coli* Strains Carrying Mcr-1 Genes in South America. Antimicrob. Agents Chemother..

[126] Perdomo A., Webb H.E., Bugarel M., Friedman C.R., Francois Watkins L.K., Loneragan G.H., Calle A. (2023). First Known Report of Mcr-Harboring Enterobacteriaceae in the Dominican Republic. Int. J. Environ. Res. Public Health.

[127] Kuleshov K.V., Pavlova A.S., Shedko E.D., Mikhaylova Y.V., Margos G., Hepner S., Chebotar I.V., Korneenko E.V., Podkolzin A.T., Akimkin V.G. (2021). Mobile Colistin Resistance Genetic Determinants of Non-Typhoid *Salmonella enterica* Isolates from Russia. Microorganisms.

[128] Johura F.T., Tasnim J., Barman I., Biswas S.R., Jubyda F.T., Sultana M., George C.M., Camilli A., Seed K.D., Ahmed N. (2020). Colistin-resistant *Escherichia coli* carrying *mcr-1* in food, water, hand rinse, and healthy human gut in Bangladesh. Gut Pathog.

[129] Yang C., Han J., Berglund B., Zou H., Gu C., Zhao L., Meng C., Zhang H., Ma X., Li X. (2022). Dissemination of BlaNDM-5 and Mcr-8.1 in Carbapenem-Resistant *Klebsiella pneumoniae* and *Klebsiella quasipneumoniae* in an Animal Breeding Area in Eastern China. Front. Microbiol..

[130] Sakdinun P., Sriwongsa N., Wongmuk S. (2018). Detection of Colistin Resistance and Mcr-1 Gene in Salmonella Isolated from Feces of Poultry in Western Thailand during 2013–2016. KKU Vet. J..

[131] Dandachi I., Fayad E., Sleiman A., Daoud Z., Rolain J.M. (2020). Dissemination of Multidrug-Resistant and Mcr-1 Gram-Negative Bacilli in Broilers, Farm Workers, and the Surrounding Environment in Lebanon. Microb. Drug Resist..

[132] Al-Mir H., Osman M., Azar N., Madec J.Y., Hamze M., Haenni M. (2019). Emergence of Clinical Mcr-1-Positive *Escherichia coli* in Lebanon. J. Glob. Antimicrob. Resist..

[133] Wang X., Sun N., Liu X., Li F., Sun J., Huang J., Li R., Wang L. (2022). Small Clone Dissemination of TmexCD1-ToprJ1–Carrying *Klebsiella pneumoniae* Isolates in a Chicken Farm. J. Glob. Antimicrob. Resist..

[134] Liu J., Yang Y., Li Y., Liu D., Tuo H., Wang H., Call D.R., Davis M., Zhang A. (2018). Isolation of an IncP-1 plasmid harbouring mcr-1 from a chicken isolate of citrobacter braakii in China. Int. J. Antimicrob. Agents.

[135] Li F., Cheng P., Li X., Liu R., Liu H., Zhang X. (2022). Molecular Epidemiology and Colistin-Resistant Mechanism of Mcr-Positive and Mcr-Negative *Escherichia coli* Isolated from Animal in Sichuan Province, China. Front. Microbiol..

[136] Yang L., Shen Y., Jiang J., Wang X., Shao D., Lam M.M.C., Holt K.E., Shao B., Wu C., Shen J. (2022). Distinct Increase in Antimicrobial Resistance Genes among *Escherichia coli* during 50 Years of Antimicrobial Use in Livestock Production in China. Nat. Food.

[137] Tang B., Chang J., Chen Y., Lin J., Xiao X., Xia X., Lin J., Yang H., Zhao G. (2022). *Escherichia fergusonii*, an Underrated Repository for Antimicrobial Resistance in Food Animals. Microbiol. Spectr..

[138] Tang B., Chen Y., Zhang L., Chang J., Xia X., Yang H. (2020). Complete Genome Sequence of Colistin-Resistant Escherichia Fergusonii Strain EFCF056. Microbiol. Resour. Announc..

[139] Lei C.W., Zhang Y., Wang Y.T., Wang H.N. (2020). Detection of Mobile Colistin Resistance Gene Mcr-10.1 in a Conjugative Plasmid from Enterobacter Roggenkampii of Chicken Origin in China. Antimicrob. Agents Chemother..

[140] Shen Z., Wang Y., Shen Y., Shen J., Wu C. (2016). Early emergence of mcr-1 in Escherichia coli from food-producing animals. Lancet Infect. Dis..

[141] Sun J., Li X.P., Yang R.S., Fang L.X., Huo W., Li S.M., Jiang P., Liao X.P., Liu Y.H. (2016). Complete Nucleotide Sequence of an IncI2 Plasmid Coharboring blaCTX-M-55 and mcr-1. Antimicrob. Agents Chemother..

[142] Li X., Li L., Yu L., Liu S., Liu L., Wei X., Song Y., Liu C., Jiang M., Wang F. (2020). Prevalence of Avian-Origin Mcr-1–Positive *Escherichia coli* with a Potential Risk to Humans in Tai’an, China. Poult. Sci..

[143] Wu C., Wang Y., Shi X., Wang S., Ren H., Shen Z., Wang Y., Lin J., Wang S. (2018). Rapid Rise of the ESBL and Mcr-1 Genes in *Escherichia coli* of Chicken Origin in China, 2008–2014. Emerg. Microbes Infect..

[144] Yang Y.Q., Li Y.X., Song T., Yang Y.X., Jiang W., Zhang A.Y., Guo X.Y., Liu B.H., Wang Y.X., Lei C.W. (2017). Colistin Resistance Gene Mcr-1 and Its Variant in *Escherichia coli* Isolates from Chickens in China. Antimicrob. Agents Chemother..

[145] Chen L., Zhang J., Wang J., Butaye P., Kelly P., Li M., Yang F., Gong J., Yassin A.K., Guo W. (2018). Newly Identified Colistin Resistance Genes, Mcr-4 and Mcr-5, from Upper and Lower Alimentary Tract of Pigs and Poultry in China. PLoS ONE.

[146] Zhang P., Wang J., Wang X., Bai X., Ma J., Dang R., Xiong Y., Fanning S., Bai L., Yang Z. (2019). Characterization of Five *Escherichia coli* Isolates Co-Expressing ESBL and Mcr-1 Resistance Mechanisms from Different Origins in China. Front. Microbiol..

[147] Zou M., Ma P.P., Liu W.S., Liang X., Li X.Y., Li Y.Z., Liu B.T. (2021). Prevalence and Antibiotic Resistance Characteristics of Extraintestinal Pathogenic *Escherichia coli* among Healthy Chickens from Farms and Live Poultry Markets in China. Animals.

[148] Yang R.S., Feng Y., Lv X.Y., Duan J.H., Chen J., Fang L.X., Xia J., Liao X.P., Sun J., Liu Y.H. (2016). Emergence of NDM-5- and MCR-1-Producing *Escherichia coli* Clones ST648 and ST156 from a Single Muscovy Duck (*Cairina moschata*). Antimicrob. Agents Chemother..

[149] Yao X., Doi Y., Zeng L., Lv L., Liu J.H. (2016). Carbapenem-Resistant and Colistin-Resistant *Escherichia coli* Co-Producing NDM-9 and MCR-1. Lancet Infect. Dis..

[150] Liu Z., Liu Y., Xi W., Liu S., Liu J., Mu H., Chen B., He H., Fan Y., Ma W. (2021). Genetic Features of Plasmid- and Chromosome-Mediated Mcr-1 in *Escherichia coli* Isolates from Animal Organs with Lesions. Front. Microbiol..

[151] Sun S., Gao H., Liu Y., Jin L., Wang R., Wang X., Wang Q., Yin Y., Zhang Y., Wang H. (2020). Co-existence of a novel plasmid-mediated efflux pump with colistin resistance gene mcr in one plasmid confers transferable multidrug resistance in *Klebsiella pneumoniae*. Emerg. Microbes Infect..

[152] Wang Y., Lyu N., Liu F., Liu W.J., Bi Y., Zhang Z., Ma S., Cao J., Song X., Wang A. (2021). More diversified antibiotic resistance genes in chickens and workers of the live poultry markets. Environ. Int..

[153] Mei C.-Y., Jiang Y., Ma Q.-C., Lu M.-J., Wu H., Wang Z.-Y., Jiao X., Wang J. (2022). Chromosomally and Plasmid-Located Mcr in Salmonella from Animals and Food Products in China. Microbiol. Spectr..

[154] Li W., Li Y., Jia Y., Sun H., Zhang C., Hu G., Yuan L. (2021). Genomic Characteristics of Mcr-1 and BlaCTX-M-Type in a Single Multidrug-Resistant *Escherichia coli* ST93 from Chicken in China. Poult. Sci..

[155] Zhang C., Feng Y., Liu F., Jiang H., Qu Z., Lei M., Wang J., Zhang B., Hu Y., Ding J. (2017). A Phage-like IncY Plasmid Carrying the Mcr-1 Gene in *Escherichia coli* from a Pig Farm in China. Antimicrob. Agents Chemother..

[156] Wang R., van Dorp L., Shaw L.P., Bradley P., Wang Q., Wang X., Jin L., Zhang Q., Liu Y., Rieux A. (2018). The Global Distribution and Spread of the Mobilized Colistin Resistance Gene Mcr-1. Nat. Commun..

[157] Liu F., Zhang R., Yang Y., Li H., Wang J., Lan J., Li P., Zhu Y., Xie Z., Jiang S. (2020). Occurrence and Molecular Characteristics of Mcr-1- Positive *Escherichia coli* from Healthy Meat Ducks in Shandong Province of China. Animals.

[158] Liu L., Feng Y., Zhang X., McNally A., Zong Z. (2017). New Variant of Mcr-3 in an Extensively Drug-Resistant *Escherichia coli* Clinical Isolate Carrying Mcr-1 and Bla_NDM-5_. Antimicrob. Agents Chemother..

[159] Liu B.T., Song F.J., Zou M., di Zhang Q., Shan H. (2017). High Incidence of *Escherichia coli* Strains Coharboring Mcr-1 and BlaNDM from Chickens. Antimicrob. Agents Chemother..

[160] Liu X., Geng S., Chan E.W.-C., Chen S. (2019). Increased Prevalence of *Escherichia coli* Strains from Food Carrying BlaNDM and Mcr-1-Bearing Plasmids That Structurally Resemble Those of Clinical Strains, China, 2015 to 2017. Eurosurveillance.

[161] Wang Y., Xu C., Zhang R., Chen Y., Shen Y., Hu F., Liu D., Lu J., Guo Y., Xia X. (2020). Changes in Colistin Resistance and Mcr-1 Abundance in *Escherichia coli* of Animal and Human Origins Following the Ban of Colistin-Positive Additives in China: An Epidemiological Comparative Study. Lancet Infect. Dis..

[162] Zhang W., Zhang T., Wang C., Liang G., Lu Q., Wen G., Guo Y., Cheng Y., Wang Z., Shao H. (2022). Prevalence of Colistin Resistance Gene Mcr-1 in *Escherichia coli* Isolated from Chickens in Central China, 2014 to 2019. J. Glob. Antimicrob. Resist..

[163] Fan R., Li C., Duan R., Qin S., Liang J., Xiao M., Lv D., Jing H., Wang X. (2020). Retrospective Screening and Analysis of Mcr-1 and BlaNDM in Gram-Negative Bacteria in China, 2010. Front. Microbiol..

[164] Lin J., Tang B., Zheng X., Chang J., Ma J., He Y., Yang H., Wu Y. (2022). Emergence of Incl2 Plasmid-Mediated Colistin Resistance in Avian Escherichia Fergusonii. FEMS Microbiol. Lett..

[165] Tang B., Wang J., Zheng X., Chang J., Ma J., Wang J., Ji X., Yang H., Ding B. (2022). Antimicrobial Resistance Surveillance of *Escherichia coli* from Chickens in the Qinghai Plateau of China. Front. Microbiol..

[166] Yang Y.Q., Zhang A.Y., Ma S.Z., Kong L.H., Li Y.X., Liu J.X., Davis M.A., Guo X.Y., Liu B.H., Lei C.W. (2016). Co-Occurrence of Mcr-1 and ESBL on a Single Plasmid in *Salmonella enterica*. J. Antimicrob. Chemother..

[167] Sun C., Cui M., Zhang S., Wang H., Song L., Zhang C., Zhao Q., Liu D., Wang Y., Shen J. (2019). Plasmid-Mediated Tigecycline-Resistant Gene Tet(X4) in *Escherichia coli* from Food-Producing Animals, China, 2008–2018. Emerg. Microbes Infect..

[168] Xu L., Wan F., Fu H., Tang B., Ruan Z., Xiao Y., Luo Q. (2022). Emergence of Colistin Resistance Gene Mcr-10 in Enterobacterales Isolates Recovered from Fecal Samples of Chickens, Slaughterhouse Workers, and a Nearby Resident. Microbiol. Spectr..

[169] Wang X., Zhai W., Wang S., Shen Z., Wang Y., Zhang Q. (2020). A Novel Transposon, Tn6518, Mediated Transfer of Mcr-3 Variant in ESBL-Producing Aeromonas Veronii. Infect. Drug Resist..

[170] Wang X., Zhai W., Li J., Liu D., Zhang Q., Shen Z., Wang S., Wang Y. (2018). Presence of an Mcr-3 Variant in Aeromonas Caviae, Proteus Mirabilis, and *Escherichia coli* from One Domestic Duck. Antimicrob. Agents Chemother..

[171] Ling Z., Yin W., Li H., Zhang Q., Wang X., Wang Z., Ke Y., Wang Y., Shena J. (2017). Chromosome-Mediated Mcr-3 Variants in Aeromonas Veronii from Chicken Meat. Antimicrob. Agents Chemother..

[172] Lv D., Duan R., Fan R., Mu H., Liang J., Xiao M., He Z., Qin S., Yang J., Jing H. (2021). BlaNDM and Mcr-1 to Mcr-5 Gene Distribution Characteristics in Gut Specimens from Different Regions of China. Antibiotics.

[173] Haroon A., Abbas M.A., Rahim A., Siddique N. (2019). The Mcr-1 Gene Transformation through Conjugation Assay in Avian Pathogenic *E. coli* from Pakistan. Int. J. Biosci..

[174] Azam M., Ehsan I., Sajjad-ur-Rahman, Saleemi M.K., Javed M.R., Mohsin M. (2017). Detection of the Colistin Resistance Gene Mcr-1 in Avian Pathogenic *Escherichia coli* in Pakistan. J. Glob. Antimicrob. Resist..

[175] Lv J., Mohsin M., Lei S., Srinivas S., Wiqar R.T., Lin J., Feng Y. (2018). Discovery of a Mcr-1-Bearing Plasmid in Commensal Colistin-Resistant *Escherichia coli* from Healthy Broilers in Faisalabad, Pakistan. Virulence.

[176] Javed H., Saleem S., Zafar A., Ghafoor A., Bin Shahzad A., Ejaz H., Junaid K., Jahan S. (2020). Emergence of Plasmid-Mediated Mcr Genes from Gram-Negative Bacteria at the Human-Animal Interface. Gut Pathog..

[177] Rafique M., Potter R.F., Ferreiro A., Wallace M.A., Rahim A., Ali Malik A., Siddique N., Abbas M.A., D’Souza A.W., Burnham C.A.D. (2020). Genomic Characterization of Antibiotic Resistant *Escherichia coli* Isolated from Domestic Chickens in Pakistan. Front. Microbiol..

[178] Zulqarnain M., Sarwar N., Anjum A.A., Firyal S., Yaqub T., Rabbani M. (2021). Molecular Detection of Colistin Resistance Gene (MCR-1) in *E. coli* Isolated from Cloacal Swabs of Broilers. Pak. Vet. J..

[179] Li R., Lu X., Munir A., Abdullah S., Liu Y., Xiao X., Wang Z., Mohsin M. (2022). Widespread Prevalence and Molecular Epidemiology of Tet(X4) and Mcr-1 Harboring *Escherichia coli* Isolated from Chickens in Pakistan. Sci. Total Environ..

[180] Shafiq M., Rahman S.U., Bilal H., Ullah A., Noman S.M., Zeng M., Yuan Y., Xie Q., Li X., Jiao X. (2022). Incidence and Molecular Characterization of ESBL-Producing and Colistin-Resistant *Escherichia coli* Isolates Recovered from Healthy Food-Producing Animals in Pakistan. J. Appl. Microbiol..

[181] Ali M.W., Utsho K.S., Karmakar S., Hoque M.N., Rahman M.T., Hassan J. (2023). First Report on the Molecular Characteristics of Mcr-1 Colistin Resistant *E. coli* Isolated from Retail Broiler Meat in Bangladesh. Int. J. Food Microbiol..

[182] Jamil A., Zahoor M.A., Nawaz Z., Siddique A.B., Khurshid M. (2022). Genetic Diversity of *Escherichia coli* Coharboring *mcr*-1 and Extended Spectrum Beta-Lactamases from Poultry. Biomed Res. Int..

[183] Uddin M.B., Alam M.N., Hasan M., Hossain S.M.B., Debnath M., Begum R., Samad M.A., Hoque S.F., Chowdhury M.S.R., Rahman M.M. (2022). Molecular Detection of Colistin Resistance Mcr-1 Gene in Multidrug-Resistant *Escherichia coli* Isolated from Chicken. Antibiotics.

[184] Uddin M.B., Hossain S.B., Hasan M., Alam M.N., Debnath M., Begum R., Roy S., Harun-Al-rashid A., Chowdhury M.S.R., Rahman M.M. (2021). Multidrug Antimicrobial Resistance and Molecular Detection of MCR-1 Gene in Salmonella Species Isolated from Chicken. Animals.

[185] Hmede Z., Kassem I.I. (2018). The Colistin Resistance Gene Mcr-1 Is Prevalent in Commensal *Escherichia coli* Isolated from Preharvest Poultry in Lebanon. Antimicrob. Agents Chemother..

[186] Kassem I.I., Mann D., Li S., Deng X. (2021). Draft Genome Sequences and Resistome Analysis of Multidrug-Resistant Mcr-1-Harbouring *Escherichia coli* Isolated from Pre-Harvest Poultry in Lebanon. J. Glob. Antimicrob. Resist..

[187] Mikhayel M., Leclercq S.O., Sarkis D.K., Doublet B. (2021). Occurrence of the Colistin Resistance Gene Mcr-1 and Additional Antibiotic Resistance Genes in ESBL/AmpC-Producing *Escherichia coli* from Poultry in Lebanon: A Nationwide Survey. Microbiol. Spectr..

[188] Abou Fayad A., el Azzi M., Sleiman A., Kassem I.I., Bawazeer R.A., Okdah L., Doumith M., Alghoribi M.F., Matar G.M. (2022). Acquired Resistome and Plasmid Sequencing of Mcr-1 Carrying MDR Enterobacteriaceae from Poultry and Their Relationship to STs Associated with Humans. JAC Antimicrob. Resist..

[189] Aklilu E., Harun A., Singh K.K.B. (2022). Molecular characterization of bla_NDM_, bla_OXA-48_, mcr-1 and bla_TEM-52_ positive and concurrently carbapenem and colistin resistant and extended spectrum beta-lactamase producing Escherichia coli in chicken in Malaysia. BMC Vet Res..

[190] Hu Y., Liu F., Lin I.Y.C., Gao G.F., Zhu B. (2016). Dissemination of the Mcr-1 Colistin Resistance Gene. Lancet Infect. Dis..

[191] Lemlem M., Aklilu E., Mohammed M., Kamaruzzaman F., Zakaria Z., Harun A., Devan S.S. (2023). Molecular detection and antimicrobial resistance profiles of Extended-Spectrum Beta-Lactamase (ESBL) producing *Escherichia coli* in broiler chicken farms in Malaysia. PLoS ONE.

[192] Karim M.R., Zakaria Z., Hassan L., Mohd Faiz N., Ahmad N.I. (2023). Antimicrobial Resistance Profiles and Co-Existence of Multiple Antimicrobial Resistance Genes in mcr-Harbouring Colistin-Resistant Enterobacteriaceae Isolates Recovered from Poultry and Poultry Meats in Malaysia. Antibiotics.

[193] Maamar E., Alonso C.A., Hamzaoui Z., Dakhli N., Abbassi M.S., Ferjani S., Saidani M., Boutiba-Ben Boubaker I., Torres C. (2018). Emergence of Plasmid-Mediated Colistin-Resistance in CMY-2-Producing *Escherichia coli* of Lineage ST2197 in a Tunisian Poultry Farm. Int. J. Food Microbiol..

[194] Oueslati W., Rjeibi M.R., Benyedem H., Mamlouk A., Souissi F., Selmi R., Ettriqui A. (2022). Prevalence, Risk Factors, Antimicrobial Resistance and Molecular Characterization of Salmonella in Northeast Tunisia Broiler Flocks. Vet. Sci..

[195] Hadjadj L., Riziki T., Zhu Y., Li J., Diene S.M., Rolain J.M. (2017). Study of Mcr-1 Gene-Mediated Colistin Resistance in Enterobacteriaceae Isolated from Humans and Animals in Different Countries. Genes.

[196] Moawad A.A., Hotzel H., Neubauer H., Ehricht R., Monecke S., Tomaso H., Hafez H.M., Roesler U., El-Adawy H. (2018). Antimicrobial Resistance in Enterobacteriaceae from Healthy Broilers in Egypt: Emergence of Colistin-Resistant and Extended-Spectrum β-Lactamase-Producing. Escherichia coli. Gut Pathog..

[197] Ramadan H., Soliman A.M., Hiott L.M., Elbediwi M., Woodley T.A., Chattaway M.A., Jenkins C., Frye J.G., Jackson C.R. (2021). Emergence of Multidrug-Resistant *Escherichia coli* Producing CTX-M, MCR-1, and FosA in Retail Food from Egypt. Front. Cell. Infect. Microbiol..

[198] Moawad A.A., Hotzel H., Hafez H.M., Ramadan H., Tomaso H., Braun S.D., Ehricht R., Diezel C., Gary D., Engelmann I. (2022). Occurrence, Phenotypic and Molecular Characteristics of Extended-Spectrum Beta-Lactamase-Producing Escherichia coli in Healthy Turkeys in Northern Egypt. Antibiotics.

[199] Perreten V., Strauss C., Collaud A., Gerber D. (2016). Colistin Resistance Gene Mcr-1 in Avian-Pathogenic *Escherichia coli* in South Africa. Antimicrob. Agents Chemother..

[200] Hassan I.Z., Wandrag B., Gouws J.J., Qekwana D.N., Naidoo V. (2021). Antimicrobial Resistance and Mcr-1 Gene in *Escherichia coli* Isolated from Poultry Samples Submitted to a Bacteriology Laboratory in South Africa. Vet. World.

[201] Takawira F.T., Pitout J.D.D., Thilliez G., Mashe T., Gutierrez A.V., Kingsley R.A., Peirano G., Matheu J., Midzi S.M., Mwamakamba L.W. (2022). Faecal Carriage of ESBL Producing and Colistin Resistant *Escherichia coli* in Avian Species over a 2-Year Period (2017–2019) in Zimbabwe. Front. Cell. Infect. Microbiol..

[202] Ngbede E.O., Poudel A., Kalalah A., Yang Y., Adekanmbi F., Adikwu A.A., Adamu A.M., Mamfe L.M., Daniel S.T., Useh N.M. (2020). Identification of Mobile Colistin Resistance Genes (Mcr-1.1, Mcr-5 and Mcr-8.1) in Enterobacteriaceae and Alcaligenes Faecalis of Human and Animal Origin, Nigeria. Int. J. Antimicrob. Agents.

[203] Vinueza-Burgos C., Ortega-Paredes D., Narvaéz C., de Zutter L., Zurita J. (2019). Characterization of Cefotaxime Resistant *Escherichia coli* Isolated from Broiler Farms in Ecuador. PLoS ONE.

[204] Aworh M.K., Kwaga J.K.P., Hendriksen R.S., Okolocha E.C., Thakur S. (2021). Genetic Relatedness of Multidrug Resistant *Escherichia coli* Isolated from Humans, Chickens and Poultry Environments. Antimicrob. Resist. Infect. Control.

[205] Yamamoto Y., Calvopina M., Izurieta R., Villacres I., Kawahara R., Sasaki M., Yamamoto M. (2019). Colistin-Resistant *Escherichia coli* with Mcr Genes in the Livestock of Rural Small-Scale Farms in Ecuador. BMC Res. Notes.

[206] Loayza-Villa F., Salinas L., Tijet N., Villavicencio F., Tamayo R., Salas S., Rivera R., Villacis J., Satan C., Ushiña L. (2020). Diverse *Escherichia coli* Lineages from Domestic Animals Carrying Colistin Resistance Gene Mcr-1 in an Ecuadorian Household. J. Glob. Antimicrob. Resist..

[207] Bastidas-Caldes C., Guerrero-Freire S., Ortuño-Gutiérrez N., Sunyoto T., Gomes-Dias C.A., Ramírez M.S., Calero-Cáceres W., Harries A.D., Rey J., de Waard J.H. (2023). Colistin Resistance in *Escherichia coli* and *Klebsiella pneumoniae* in Humans and Backyard Animals in Ecuador. Rev. Panam. Salud Pública.

[208] Bastidas-Caldes C., Cisneros-Vásquez E., Zambrano A., Mosquera-Maza A., Calero-Cáceres W., Rey J., Yamamoto Y., Yamamoto M., Calvopiña M., de Waard J.H. (2023). Co-Harboring of Beta-Lactamases and Mcr-1 Genes in *Escherichia coli* and *Klebsiella pneumoniae* from Healthy Carriers and Backyard Animals in Rural Communities in Ecuador. Antibiotics.

[209] Murray M., Salvatierra G., Dávila-Barclay A., Ayzanoa B., Castillo-Vilcahuaman C., Huang M., Pajuelo M.J., Lescano A.G., Cabrera L., Calderón M. (2021). Market Chickens as a Source of Antibiotic-Resistant *Escherichia coli* in a Peri-Urban Community in Lima, Peru. Front. Microbiol..

[210] Carhuaricra D., Duran Gonzales C.G., Rodríguez Cueva C.L., Ignacion León Y., Silvestre Espejo T., Marcelo Monge G., Rosadio Alcántara R.H., Lincopan N., Espinoza L.L., Maturrano Hernández L. (2022). Occurrence and Genomic Characterization of Mcr-1-Harboring *Escherichia coli* Isolates from Chicken and Pig Farms in Lima, Peru. Antibiotics.

[211] Fernandes M., Moura Q., Sartori L., Silva K., Cunha M., Esposito F., Lopes R., Otutumi L., Gonçalves D., Dropa M. (2016). Silent Dissemination of Colistin-Resistant *Escherichia coli* in South America Could Contribute to the Global Spread of the Mcr-1 Gene. Eurosurveillance.

[212] Lentz S.A., de Lima-Morales D., Cuppertino V.M., de Nunes L.S., da Motta A.S., Zavascki A.P., Barth A.L., Martins A.F. (2016). Letter to the Editor: *Escherichia coli* Harbouring Mcr-1 Gene Isolated from Poultry Not Exposed to Polymyxins in Brazil. Eurosurveillance.

[213] Moreno L.Z., Gomes V.T.M., Moreira J., de Oliveira C.H., Peres B.P., Silva A.P.S., Thakur S., La Ragione R.M., Moreno A.M. (2019). First Report of Mcr-1-Harboring Salmonella Enterica Serovar Schwarzengrund Isolated from Poultry Meat in Brazil. Diagn. Microbiol. Infect. Dis..

[214] Barbieri N.L., Pimenta R.L., de Melo D.A., Nolan L.K., de Souza M.M.S., Logue C.M. (2021). Mcr-1 Identified in Fecal *Escherichia coli* and Avian Pathogenic *E. coli* (APEC) From Brazil. Front. Microbiol..

[215] Pontes L.D.S., Pimenta R., Silveira M.C., Tavares-Teixeira C.B., Pereira N.F., da Conceiçao Neto O.C., de Oliveira Santos I.C., da Costa B.S., Carvalho-Assef A.P.D.A., de Souza M.M.S. (2021). Letter to the Editor: Escherichia Fergusonii Harboring IncHI2 Plasmid Containing Mcr-1 Gene-A Novel Reservoir for Colistin Resistance in Brazil. Microb. Drug Resist..

[216] Dominguez J.E., Figueroa Espinosa R.A., Redondo L.M., Cejas D., Gutkind G.O., Chacana P.A., Di Conza J.A., Fernández-Miyakawa M.E. (2017). Plasmid-Mediated Colistin Resistance in *Escherichia coli* Recovered from Healthy Poultry. Rev. Argent. Microbiol..

[217] Dominguez J.E., Faccone D., Tijet N., Gomez S., Corso A., Fernández-Miyakawa M.E., Melano R.G. (2019). Characterization of *Escherichia coli* carrying Mcr-1-Plasmids Recovered from Food Animals from Argentina. Front. Cell. Infect. Microbiol..

[218] Nesporova K., Valcek A., Papagiannitsis C., Kutilova I., Jamborova I., Davidova-Gerzova L., Bitar I., Hrabak J., Literak I., Dolejska M. (2021). Multi-Drug Resistant Plasmids with Esbl/Ampc and Mcr-5.1 in Paraguayan Poultry Farms: The Linkage of Antibiotic Resistance and Hatcheries. Microorganisms.

[219] Maciuca I.E., Cummins M.L., Cozma A.P., Rimbu C.M., Guguianu E., Panzaru C., Licker M., Szekely E., Flonta M., Djordjevic S.P. (2019). Genetic Features of Mcr-1 Mediated Colistin Resistance in CMY-2-Producing *Escherichia coli* From Romanian Poultry. Front. Microbiol..

[220] Bortolaia V., Ronco T., Romascu L., Nicorescu I., Milita N.M., Vaduva A.M., Leekitcharoenphon P., Kjeldgaard J.S., Hansen I.M., Svendsen C.A. (2021). Co-Localization of Carbapenem (BlaOXA-162) and Colistin (Mcr-1) Resistance Genes on a Transferable IncHI2 Plasmid in *Escherichia coli* of Chicken Origin. J. Antimicrob. Chemother..

[221] Mišić D., Kiskaroly F., Szostak M.P., Cabal A., Ruppitsch W., Bernreiter-Hofer T., Milovanovic V., Feßler A.T., Allerberger F., Spergser J. (2021). The First Report of Mcr-1-Carrying *Escherichia coli* Originating from Animals in Serbia. Antibiotics.

[222] Ortega-Paredes D., Barba P., Zurita J. (2016). Colistin-Resistant *Escherichia coli* Clinical Isolate Harbouring the Mcr-1 Gene in Ecuador. Epidemiol. Infect..

[223] Gelbíčová T., Baráková A., Florianová M., Jamborová I., Zelendová M., Pospíšilová L., Koláčková I., Karpíšková R. (2019). Dissemination and Comparison of Genetic Determinants of Mcr-Mediated Colistin Resistance in Enterobacteriaceae via Retailed Raw Meat Products. Front. Microbiol..

[224] Nishino Y., Shimojima Y., Suzuki Y., Ida M., Fukui R., Kuroda S., Hirai A., Sadamasu K. (2017). Detection of the Mcr-1 Gene in Colistin-Resistant *Escherichia coli* from Retail Meat in Japan. Microbiol. Immunol..

[225] Wang Y., Liu F., Zhu B., Gao G.F. (2020). Metagenomic data screening reveals the distribution of mobilized resistance genes tet (X), mcr and carbapenemase in animals and humans. J. Infect..

[226] Skov R.L., Monnet D.L. (2016). Plasmid-Mediated Colistin Resistance (Mcr-1 Gene): Three Months Later, the Story Unfolds. Eurosurveillance.

[227] Von Wintersdorff C.J.H., Wolffs P.F.G., Van Niekerk J.M., Beuken E., Van Alphen L.B., Stobberingh E.E., Oude Lashof A.M.L., Hoebe C.J.P.A., Savelkoul P.H.M., Penders J. (2016). Detection of the Plasmid-Mediated Colistin-Resistance Gene Mcr-1 in Faecal Metagenomes of Dutch Travellers. J. Antimicrob. Chemother..

[228] Van Boeckel T.P., Brower C., Gilbert M., Grenfell B.T., Levin S.A., Robinson T.P., Teillant A., Laxminarayan R. (2015). Global Trends in Antimicrobial Use in Food Animals. Proc. Natl. Acad. Sci. USA.

[229] Yam E.L.Y., Hsu L.Y., Yap E.P.H., Yeo T.W., Lee V., Schlundt J., Lwin M.O., Limmathurotsakul D., Jit M., Dedon P. (2019). Antimicrobial Resistance in the Asia Pacific Region: A Meeting Report. Antimicrob. Resist. Infect. Control.

[230] Gao Y., Lu C., Shen D., Liu J., Ma Z., Yang B., Ling W., Waigi M.G. (2019). Elimination of the Risks of Colistin Resistance Gene (Mcr-1) in Livestock Manure during Composting. Environ. Int..

[231] Li J., Shi X., Yin W., Wang Y., Shen Z., Ding S., Wang S. (2017). A Multiplex SYBR Green Real-Time PCR Assay for the Detection of Three Colistin Resistance Genes from Cultured Bacteria, Feces, and Environment Samples. Front. Microbiol..

[232] Valiakos G., Kapna I. (2021). Colistin Resistant Mcr Genes Prevalence in Livestock Animals (Swine, Bovine, Poultry) from a Multinational Perspective. A Systematic Review. Vet. Sci..

[233] Binsker U., Käsbohrer A., Hammerl J.A. (2022). Global Colistin Use: A Review of the Emergence of Resistant Enterobacterales and the Impact on Their Genetic Basis. FEMS Microbiol. Rev..

[234] Walsh T.R., Wu Y. (2016). China Bans Colistin as a Feed Additive for Animals. Lancet Infect. Dis..

[235] Webb H.E., Angulo F.J., Granier S.A., Scott H.M., Loneragan G.H. (2017). Illustrative Examples of Probable Transfer of Resistance Determinants from Food Animals to Humans: Streptothricins, Glycopeptides, and Colistin. F1000Research.

[236] Shen Y., Xu C., Sun Q., Schwarz S., Ou Y., Yang L., Huang Z., Eichhorn I., Walsh T.R., Wang Y. (2018). Prevalence and Genetic Analysis of Mcr-3-Positive Aeromonas Species from Humans, Retail Meat, and Environmental Water Samples. Antimicrob. Agents Chemother..

[237] Borowiak M., Baumann B., Fischer J., Thomas K., Deneke C., Hammerl J.A., Szabo I., Malorny B. (2020). Development of a Novel Mcr-6 to Mcr-9 Multiplex PCR and Assessment of Mcr-1 to Mcr-9 Occurrence in Colistin-Resistant *Salmonella enterica* Isolates from Environment, Feed, Animals and Food (2011–2018) in Germany. Front. Microbiol..

[238] Dandachi I., Chaddad A., Hanna J., Matta J., Daoud Z. (2019). Understanding the Epidemiology of Multi-Drug Resistant Gram-Negative Bacilli in the Middle East Using a One Health Approach. Front. Microbiol..

[239] Feng S., Liang W., Li J., Chen Y., Zhou D., Liang L., Lin D., Li Y., Zhao H., Du H. (2022). MCR-1-Dependent Lipid Remodelling Compromises the Viability of Gram-Negative Bacteria. Emerg. Microbes Infect..

[240] Shen C., Zhong L.L., Yang Y., Doi Y., Paterson D.L., Stoesser N., Ma F., El-Sayed Ahmed M.A.E.G., Feng S., Huang S. (2020). Dynamics of Mcr-1 Prevalence and Mcr-1-Positive *Escherichia coli* after the Cessation of Colistin Use as a Feed Additive for Animals in China: A Prospective Cross-Sectional and Whole Genome Sequencing-Based Molecular Epidemiological Study. Lancet Microbe.

[241] Jiang Y., Zhang Y., Lu J., Wang Q., Cui Y., Wang Y., Quan J., Zhao D., Du X., Liu H. (2020). Clinical Relevance and Plasmid Dynamics of Mcr-1-Positive *Escherichia coli* in China: A Multicentre Case-Control and Molecular Epidemiological Study. Lancet Microbe.

[242] Snesrud E., McGann P., Chandler M. (2018). The Birth and Demise of the ISApl1-Mcr-1-ISApl1 Composite Transposon: The Vehicle for Transferable Colistin Resistance. mBio.

[243] Shen C., Zhong L.L., Ma F., El-Sayed Ahmed M.A.E.G., Doi Y., Zhang G., Liu Y., Huang S., Li H.Y., Zhang L. (2020). Genomic Patterns and Characterizations of Chromosomally-Encoded Mcr-1 in *Escherichia coli* Populations. Gut Pathog..

[244] Wang J., Jiang Y., Ji R.Y., Wang Z.Y., Lu M.J., Wu H., Mei C.Y., Li Q.C., Jiao X. (2022). Colistin- and Tigecycline-Resistant CTX-M-14-Producing *Salmonella enterica* Serovar Kentucky ST198 from Retail Chicken Meat, China. Int. J. Antimicrob. Agents.

[245] Chang M.X., Zhang J., Zhang J.F., Ding X.M., Lu Y., Zhang J., Li R., Jiang H.X. (2022). Formation, Transmission, and Dynamic Evolution of a Multidrug-Resistant Chromosomally Integrated Plasmid in *Salmonella* spp.. Front. Microbiol..

[246] Gallardo A., Ugarte-Ruiz M., Hernández M., Miguela-Villoldo P., Rodríguez-Lázaro D., Domínguez L., Quesada A. (2020). Involvement of Hpap2 and Dgka Genes in Colistin Resistance Mediated by Mcr Determinants. Antibiotics.

[247] Yang X., Liu L., Wang Z., Bai L., Li R. (2019). Emergence of Mcr-8.2-Bearing *Klebsiella quasipneumoniae* of Animal Origin. J. Antimicrob. Chemother..

[248] Rhouma M., Letellier A. (2017). Extended-Spectrum β-Lactamases, Carbapenemases and the Mcr-1 Gene: Is There a Historical Link?. Int. J. Antimicrob. Agents.

[249] Shi L., Zhang J., Lu T., Zhang K. (2022). Metagenomics Revealed the Mobility and Hosts of Antibiotic Resistance Genes in Typical Pesticide Wastewater Treatment Plants. Sci. Total Environ..

[250] Lu X., Xiao X., Liu Y., Li R., Wang Z. (2021). Emerging Opportunity and Destiny of Mcr-1—And Tet (X4)-Coharboring Plasmids in *Escherichia coli*. Microbiol. Spectr..

[251] Kieffer N., Royer G., Decousser J.-W., Bourrel A.-S., Palmieri M., Ortiz De La Rosa J.-M., Jacquier H., Denamur E., Nordmann P., Poirel L. (2019). Mcr-9, an Inducible Gene Encoding an Acquired Phosphoethanolamine Transferase in *Escherichia coli*, and Its Origin. Antimicrob. Agents Chemother..

[252] Ohji G., Doi A., Yamamoto S., Iwata K. (2016). Is De-Escalation of Antimicrobials Effective? A Systematic Review and Meta-Analysis. Int. J. Infect. Dis..

[253] Zhang S., Sun H., Lao G., Zhou Z., Liu Z., Cai J., Sun Q. (2022). Identification of Mobile Colistin Resistance Gene Mcr-10 in Disinfectant and Antibiotic Resistant *Escherichia coli* from Disinfected Tableware. Antibiotics.

[254] Mustafa G.R., Zhao K., He X., Chen S., Liu S., Mustafa A., He L., Yang Y., Yu X., Penttinen P. (2021). Heavy Metal Resistance in Salmonella Typhimurium and Its Association with Disinfectant and Antibiotic Resistance. Front. Microbiol..

[255] Aslam B., Khurshid M., Arshad M.I., Muzammil S., Rasool M., Yasmeen N., Shah T., Chaudhry T.H., Rasool M.H., Shahid A. (2021). Antibiotic Resistance: One Health One World Outlook. Front. Cell. Infect. Microbiol..

[256] Virolle C., Goldlust K., Djermoun S., Bigot S., Lesterlin C. (2020). Plasmid Transfer by Conjugation in Gram-Negative Bacteria: From the Cellular to the Community Level. Genes.

[257] Wu R., Yi L.X., Yu L.F., Wang J., Liu Y., Chen X., Lv L., Yang J., Liu J.H. (2018). Fitness Advantage of Mcr-1-Bearing IncI2 and IncX4 Plasmids in Vitro. Front. Microbiol..

[258] Yang Q.E., Tansawai U., Andrey D.O., Wang S., Wang Y., Sands K., Kiddee A., Assawatheptawee K., Bunchu N., Hassan B. (2019). Environmental Dissemination of Mcr-1 Positive Enterobacteriaceae by *Chrysomya* Spp. (Common Blowfly): An Increasing Public Health Risk. Environ. Int..

[259] Yi L., Durand R., Grenier F., Yang J., Yu K., Burrus V., Liu J.H. (2022). PixR, a Novel Activator of Conjugative Transfer of IncX4 Resistance Plasmids, Mitigates the Fitness Cost of Mcr-1 Carriage in *Escherichia coli*. mBio.

[260] Manges A.R., Geum H.M., Guo A., Edens T.J., Fibke C.D., Pitout J.D.D. (2019). Global Extraintestinal Pathogenic *Escherichia coli* (Expec) Lineages. Clin. Microbiol. Rev..

[261] Massella E., Giacometti F., Bonilauri P., Reid C.J., Djordjevic S.P., Merialdi G., Bacci C., Fiorentini L., Massi P., Bardasi L. (2021). Antimicrobial Resistance Profile and Expec Virulence Potential in Commensal *Escherichia coli* of Multiple Sources. Antibiotics.

[262] Li W., Liu Z., Yin W., Yang L., Qiao L., Song S., Ling Z., Zheng R., Wu C., Wang Y. (2021). Mcr Expression Conferring Varied Fitness Costs on Host Bacteria and Affecting Bacteria Virulence. Antibiotics.

[263] Lu J., Quan J., Zhao D., Wang Y., Yu Y., Zhu J. (2019). Prevalence and Molecular Characteristics of Mcr-1 Gene in Salmonella Typhimurium in a Tertiary Hospital of Zhejiang Province. Infect. Drug Resist..

[264] Zheng B., Feng Y. (2019). MCR-1-Producing *Salmonella typhimurium* ST34 Links Animal Foods to Human Community Infections. eBioMedicine.

[265] Vázquez X., Forcelledo L., Balboa-Palomino S., Fernández J., Rodicio M.R. (2022). Nosocomial Pneumonia Caused in an Immunocompetent Patient by the Emergent Monophasic ST34 Variant of *Salmonella enterica* Serovar Typhimurium: Treatment-Associated Selection of Fluoroquinolone and Piperacillin/Tazobactam Resistance. Antibiotics.

[266] Ray M., Manjunath A., Halami P.M. (2022). Prevalence of Polymyxin Resistance through the Food Chain, the Global Crisis. J. Antibiot..

[267] Singh S., Pathak A., Kumar A., Rahman M., Singh A., Gonzalez-Zorn B., Prasad K.N. (2018). Emergence of Chromosome-Borne Colistin Resistance Gene Mcr-1 in Clinical Isolates of *Klebsiella pneumoniae* from India. Antimicrob. Agents Chemother..

[268] Hameed F., Khan M.A., Muhammad H., Sarwar T., Bilal H., Rehman T.U. (2019). Plasmid-Mediated Mcr-1 Gene in Acinetobacter Baumannii and Pseudomonas Aeruginosa: First Report from Pakistan. Rev. Soc. Bras. Med. Trop..

[269] Kassem I.I., Osman M. (2022). A Brewing Storm: The Impact of Economic Collapse on the Access to Antimicrobials in Lebanon. J. Glob. Antimicrob. Resist..

[270] Al-Kadmy I.M.S., Ibrahim S.A., Al-Saryi N., Aziz S.N., Besinis A., Hetta H.F. (2020). Prevalence of Genes Involved in Colistin Resistance in Acinetobacter Baumannii: First Report from Iraq. Microb. Drug Resist..

[271] Güzel M., Avşaroğlu M.D., Soyer Y. (2020). Determination of Colistin Resistance in *Escherichia coli* Isolates from Foods in Turkey, 2011–2015. Food and Health.

[272] Kansak N., Aksaray S., Aslan M., Adaleti R., Gönüllü N. (2021). Detection of Colistin Resistance among Multidrug-Resistant *Klebsiella pneumoniae* and *Escherichia coli* Clinical Isolates in Turkey. Acta Microbiol. Immunol. Hung..

[273] Pishnian Z., Haeili M., Feizi A. (2019). Prevalence and Molecular Determinants of Colistin Resistance among Commensal Enterobacteriaceae Isolated from Poultry in Northwest of Iran. Gut Pathog..

[274] Ilbeigi K., Askari Badouei M., Vaezi H., Zaheri H., Aghasharif S., Kafshdouzan K. (2021). Molecular survey of *mcr1* and *mcr2* plasmid mediated colistin resistance genes in *Escherichia coli* isolates of animal origin in Iran. BMC Res. Notes.

[275] Nhung N.T., Cuong N.v., Thwaites G., Carrique-Mas J. (2016). Antimicrobial Usage and Antimicrobial Resistance in Animal Production in Southeast Asia: A Review. Antibiotics.

[276] Olaitan A.O., Thongmalayvong B., Akkhavong K., Somphavong S., Paboriboune P., Khounsy S., Morand S., Rolain J.-M. (2015). Clonal Transmission of a Colistin-Resistant *Escherichia coli* from a Domesticated Pig to a Human in Laos. J. Antimicrob. Chemother..

[277] Sudatip D., Chasiri K., Kritiyakan A., Phanprasit W., Thinphovong C., Tiengrim S., Thamlikitkul V., Abdallah R., Baron S.A., Rolain J.M. (2021). A One Health Approach to Assessing Occupational Exposure to Antimicrobial Resistance in Thailand: The FarmResist Project. PLoS ONE.

[278] Devan S.S., Aklilu E., Hamdan R.H., Lemlem M., Zakaria Z. (2022). Detection of colistin-resistant Escherichia coli isolated from broiler chickens in Kelantan, Malaysia. Trop Biomed..

[279] Umali D.v. (2019). Antimicrobial Susceptibility Profiles and Detection of Colistin Resistance Mcr-1 Gene in Salmonella Enterica Isolates from Chicken Eggs in Metro Manila, Philippines. Philipp. J. Vet. Med..

[280] Founou L.L., Founou R.C., Essack S.Y. (2016). Antibiotic Resistance in the Food Chain: A Developing Country-Perspective. Front. Microbiol..

[281] Chabou S., Leangapichart T., Okdah L., Le Page S., Hadjadj L., Rolain J.M. (2016). Real-Time Quantitative PCR Assay with Taqman^®^ Probe for Rapid Detection of MCR-1 Plasmid-Mediated Colistin Resistance. New Microbes New. Infect..

[282] Di Francesco A., Salvatore D., Sakhria S., Bertelloni F., Catelli E., Ben Yahia S., Tlatli A. (2023). Colistin Resistance Genes in Broiler Chickens in Tunisia. Animals.

[283] Dhaouadi S., Romdhani A., Bouglita W., Chedli S., Chaari S., Soufi L., Cherif A., Mnif W., Abbassi M.S., Elandoulsi R.B. (2023). High Biofilm-Forming Ability and Clonal Dissemination among Colistin-Resistant *Escherichia coli* Isolates Recovered from Cows with Mastitis, Diarrheic Calves, and Chickens with Colibacillosis in Tunisia. Life.

[284] Cummins M.L., Reid C.J., Chowdhury P.R., Bushell R.N., Esbert N., Tivendale K.A., Noormohammadi A.H., Islam S., Marenda M.S., Browning G.F. (2019). Whole Genome Sequence Analysis of Australian Avian Pathogenic *Escherichia coli* That Carry the Class 1 Integrase Gene. Microb. Genom..

[285] Zakaria A.S., Edward E.A., Mohamed N.M. (2021). Genomic Insights into a Colistin-Resistant Uropathogenic *Escherichia coli* Strain of O23:H4-St641 Lineage Harboring Mcr-1.1 on a Conjugative Inchi2 Plasmid from Egypt. Microorganisms.

[286] Abdel-Rahman M.A.A., Hamed E.A., Abdelaty M.F., Sorour H.K., Badr H., Hassan W.M., Shalaby A.G., Mohamed A.A., Soliman M.A., Roshdy H. (2023). Distribution pattern of antibiotic resistance genes in *Escherichia coli* isolated from colibacillosis cases in broiler farms of Egypt. Vet World.

[287] Theobald S., Etter E.M.C., Gerber D., Abolnik C. (2019). Antimicrobial Resistance Trends in *Escherichia coli* in South African Poultry: 2009–2015. Foodborne Pathog. Dis..

[288] Labuschagne Q., Schellack N., Gous A., Bronkhorst E., Schellack G., Van Tonder L., Truter A., Smith C., Lancaster R., Kolman S. (2016). COLISTIN: Adult and Paediatric Guideline for South Africa. S. Afr. J. Infect. Dis..

[289] Ngbede E.O., Adekanmbi F., Poudel A., Kalalah A., Kelly P., Yang Y., Adamu A.M., Daniel S.T., Adikwu A.A., Akwuobu C.A. (2021). Concurrent Resistance to Carbapenem and Colistin Among Enterobacteriaceae Recovered from Human and Animal Sources in Nigeria Is Associated with Multiple Genetic Mechanisms. Front. Microbiol..

[290] Mendelson M., Brink A., Gouws J., Mbelle N., Naidoo V., Pople T., Schellack N., van Vuuren M., Rees H., Banoo S. (2018). The One Health Stewardship of Colistin as an Antibiotic of Last Resort for Human Health in South Africa. Lancet Infect. Dis..

[291] Büdel T., Kuenzli E., Clément M., Bernasconi O.J., Fehr J., Mohammed A.H., Hassan N.K., Zinsstag J., Hatz C., Endimiani A. (2019). Polyclonal Gut Colonization with Extended-Spectrum Cephalosporin-and/or Colistin-Resistant Enterobacteriaceae: A Normal Status for Hotel Employees on the Island of Zanzibar, Tanzania. J. Antimicrob. Chemother..

[292] Dávalos-Almeyda M., Guerrero A., Medina G., Dávila-Barclay A., Salvatierra G., Calderón M., Gilman R.H., Tsukayama P. (2022). Antibiotic Use and Resistance Knowledge Assessment of Personnel on Chicken Farms with High Levels of Antimicrobial Resistance: A Cross-Sectional Survey in Ica, Peru. Antibiotics.

[293] Rabello R.F., Bonelli R.R., Penna B.A., Albuquerque J.P., Souza R.M., Cerqueira A.M.F. (2020). Antimicrobial Resistance in Farm Animals in Brazil: An Update Overview. Animals.

[294] Rau R.B., De Lima-Morales D., Wink P.L., Ribeiro A.R., Martins A.F., Barth A.L. (2018). Emergence of Mcr-1 Producing Salmonella Enterica Serovar Typhimurium from Retail Meat: First Detection in Brazil. Foodborne Pathog. Dis..

[295] Xu F., Zeng X., Hinenoya A., Lin J. (2018). MCR-1 Confers Cross-Resistance to Bacitracin, a Widely Used In-Feed Antibiotic. mSphere.

[296] EUCAST EUCAST. https://eucast.org/.

[297] Quiroga C., Nastro M., Di Conza J. (2019). Current Scenario of Plasmid-Mediated Colistin Resistance in Latin America. Rev. Argent. Microbiol..

[298] Webb H.E., Kim J.Y., Tagg K.A., Kapsak C.J., Tobolowsky F., Birhane M.G., Francois Watkins L., Folster J.P. (2022). Fourteen Mcr-1-Positive *Salmonella enterica* Isolates Recovered from Travelers Returning to the United States from the Dominican Republic. Microbiol. Resour. Announc..

[299] Andrade F.F., Silva D., Rodrigues A., Pina-Vaz C. (2020). Colistin Update on Its Mechanism of Action and Resistance, Present and Future Challenges. Microorganisms.

[300] Miranda C., Igrejas G., Capita R., Alonso-Calleja C., Poeta P. (2022). Worldwide Colistin Use and Spread of Resistant- Enterobacteriaceae in Animal Production. The Global Antimicrobial Resistance Epidemic–Innovative Approaches and Cutting-Edge Solutions [200Working Title].

[301] Ageevets V., Lazareva I., Mrugova T., Gostev V., Lobzin Y., Sidorenko S. (2019). IncX4 Plasmids Harbouring Mcr-1 Genes: Further Dissemination. J. Glob. Antimicrob. Resist..

[302] Tarabai H., Valcek A., Jamborova I., Vazhov S.V., Karyakin I.V., Raab R., Literak I., Dolejska M. (2019). Plasmid-Mediated *mcr-1* Colistin Resistance in Escherichia coli from a Black Kite in Russia. Antimicrob Agents Chemother.

[303] Jovčić B., Novović K., Filipić B., Velhner M., Todorović D., Matović K., Rašić Z., Nikolić S., Kiškarolj F., Kojić M. (2020). Genomic Characteristics of Colistin-Resistant Salmonella Enterica Subsp. Enterica Serovar Infantis from Poultry Farms in the Republic of Serbia. Antibiotics.

[304] Okpala C.O.R., Anyanwu M.U., Łukanko S., Nwobi O.C., Korzeniowska M., Ezeonu I.M. (2021). Animal-Food-Human Antimicrobial Resistance Fundamentals, Prevention Mechanisms and Global Surveillance Trends: A Terse Review. Appl. Food Biotechnol..

