# Supplementary material for: Mobile Colistin Resistance (mcr) Gene-Containing Organisms in Poultry Sector in Low- and Middle-Income Countries: Epidemiology, Characteristics, and One Health Control Strategies
Source: Antibiotics (Basel). 2023 Jun 28;12(7):1117. doi: 10.3390/antibiotics12071117 (PMC10376608; doi:10.3390/antibiotics12071117)
Supplement: Supplementary file 1 [file antibiotics-12-01117-s001.zip › antibiotics-2440343-supplementary.pdf]

Table S1: Studies reporting on plasmid mediated colistin resistance in the poultry meat supply chain in China

Source of isolate	Date of isolation (*mcr* gene assayed)	Number of isolates tested for *mcr*	Identified gene/variant (Number of organism)	Sequence type and/or phylogroup (Virulence genes)	Plasmid (Associated Insertion sequence)	Additional resistance traits	Reference
Chicken meats	2011-2014 (*mcr*-1)	357	*mcr*-1 (35 *E. coli*)	-	IncI2 and IncHI2 (IS*AplI*)	*fosA3*, $bla_{CTX\text{-}M\text{-}44}$ and *oqxB*	Liu *et al.* (2016)*; Chipping Zhu *et al.* (2016)*
Chickens	1970-2014 (*mcr*-1)	1160	*mcr*-1 (104 *E. coli*)	-	-	-	Shen *et al.* (2016)*
Mucovy duck	2015 (*mcr*-1)	2	*mcr*-1(2 *E. coli*)	ST648 and ST156 (B1 and D)	IncI2 and IncHI2	$bla_{NDM\text{-}5}$, $bla_{CTX\text{-}M\text{-}55}$, $bla_{TEM\text{-}1}$, $bla_{CMY\text{-}2}$, *rmtB*, *aac(6')-Ib* and *fosA3*	Yang *et al.* (2016)*
Chickens and ducks	2014-2015 (*mcr*-1 to *mcr*-3)	448	*mcr*-1 (13 *E. coli* from chickens and 3 *E. coli* from ducks)	-	-	-	Yassin *et al.* (2017)*
Chickens	2015 (*mcr*-1)	3	*mcr*-1 (2 *Cronobacter sakazakii* and 1 *E. coli*)	-	-	*C. sakazakii*: *floR*, *qnrS*, $bla_{NDM\text{-}9}$, *fosA*, $bla_{CTX\text{-}M\text{-}65}$ and $bla_{CTX\text{-}M\text{-}55}$; *E. coli*: as above except *qnrS*, $bla_{CTX\text{-}M\text{-}55}$ and $bla_{NDM\text{-}9}$	Liu *et al.* (2017)*
Chicken meat	2014 (*mcr*-1)	1	*mcr*-1 (1 *E. coli*)	ST167	IncI2	$bla_{NDM\text{-}9}$, *fosA3*, *rmtB*, $bla_{CTX\text{-}M\text{-}65}$ and *floR*	Yao *et al.* (2016)*
Chicken	2012 (*mcr*-1)	1	*mcr*-1 (1 *E. coli*)	ST1011	IncI2 (IS*Apl1* for *mcr*-1 and IS*Ecp1* for ESBL gene)	$bla_{CTX\text{-}M\text{-}55}$	Sun *et al.* (2016)*
Chickens	2007-2015 (*mcr*-1)	16	*mcr*-1 (2 salmonellae)	ST34	IncHI2, IncI2, IncFIB and IncFII	*oqxAB*, *aac(6')-Ib-cr* and *floR*	Li *et al.* (2016)*
Chickens	2014-2015 (*mcr*-1)	53	*mcr*-1 (4 salmonellae)	ST292 *Salmonella enterica* serovar Albany	IncI2 (IS*Apl1* for *mcr*-1 and IS*Ecp1* for ESBL gene)	$bla_{CTX\text{-}M\text{-}55}$	Yang *et al.* (2016)*
Chickens	2011-2012 (*mcr*-1)	96	*mcr*-1 (2 *E. coli*)	ST117 and ST22	IncFIC,IncFII, IncFIB, IncI1 and IncI2	Class 1 integron, $bla_{TEM\text{-}1B}$, $bla_{TEM\text{-}99}$, $bla_{CTX\text{-}M\text{-}14}$, *qacE*, sul1, *dfrA17*, *catA1*, *tet*(B) and *aadA5*	Ding *et al.* (2018)*
Chickens	2015-2016 (*mcr*-1)	262	*mcr*-1 (6 *E. coli*)	-	IncY	-	Zhang *et al.* (2017)*
Chickens	2010-2014 (*mcr*-1)	981	*mcr*-1 (45 *E. coli*)	-	-	-	Chen *et al.* (2017)*
Chickens	2007-2014 (*mcr*-1)	200	*mcr*-1 (182 *E. coli*)	-	-	-	Huang *et al.* (2017)*
Chickens, drinking water, slaughterhouse, and farm sewage	2016 (*mcr*-1)	228	*mcr*-1 (102 *E. coli* and 1 *Klebsiella pneumoniae*)	ST156, ST6388, ST2736, ST155, ST206, ST224, ST156, ST2599, ST648, ST5498, ST162, ST354 and ST48	IncHI2, IncI2 and IncFIB	$bla_{NDM\text{-}9}$, $bla_{NDM\text{-}5}$ and $bla_{NDM\text{-}4}$	Wang *et al.* (2018)*

Chickens	2010-2015 (*mcr*-1)	1136	*mcr*-1 (58 *E. coli*) and *mcr*-1.3 (1 *E. coli*)	ST48, ST10, ST616, ST88, ST77, ST542, ST5879, ST5865, ST1431, ST1290, ST873, ST971, ST952, ST5851, ST101, ST761, ST6050, ST351, ST361, ST744, ST117, ST3044, ST2491, ST2345, ST1642, ST5909, ST601, ST3944, ST870, ST3133, ST617, ST215, ST178, ST58, ST155, ST3481 and ST5542	IncI2 (IS*2*)	$bla_{\text{CTX-M-1}}$ and $bla_{\text{CTX-M-9}}$	Yang *et al.* (2017)*
Chickens and chicken meats	2017 (*mcr*-1 to *mcr*-7)	3	*mcr*-3.15 and *mcr*-3-like2 (1 *Aeromonas media*), *mcr*-3.16 and *mcr*-3-like4 (1 *A. salmonicida*), and *mcr*-3.17 and *mcr*-3-lik3 (1 *A. allosaccharophila*)	*ahh1*	IS*Kpn3* and IS*As17*	$bla_{\text{OXA-10}}$, *cmlA1*, *dfrA14*, *mph(A)*, *tet(E)*, *aadA1* $bla_{\text{CEPH-A3-like}}$, $bla_{\text{FOX-2}}$, $bla_{\text{OXA-12}}$, *dfrA17*, *sul1* and *tet(E)*	Shen *et al.* (2018)***
Chickens	2015-2016 (*mcr*-1 and *mcr*-2)	443	*mcr*-1 (388 *E. coli*) and *mcr*-2 (66 *E. coli*); *mcr*-1 and *mcr*-2 (32 *E. coli*)	-	-	-	Zhang *et al.* (2018)*
Chickens	2016 (*mcr*-1-*mcr*-8)	15	*mcr*-8.1 and *mcr*-8.4 (2 *Raoultella ornithinolytica*)	-	IncFII (ΔIS*903B*)	*aadA1*, *aph(3')-Ia*, *strA*, *strB*, *aac(6')-Ib*, *armA*, *fosA*, *mph(E)*, *floR*, *cml*, *qnrB4*, *sul*, *tet*(B), *tet*(34), $bla_{\text{TEM-1B}}$, $bla_{\text{OXA-1}}$, $bla_{\text{DHA-1}}$, *aac(3)-IVa*, *aph(4)-Ia*, *aadA2*, *mph(A)*, *cat*, *qnrS4*, *oqxAB*, *qnrB52*, *sul1*, *sul2*, *sul3*, *tet*(A), *tet*(O), *tet*(B) and $bla_{\text{SHV-73}}$	W ang *et al.* (2019)*
Chickens	2010-2015 (*mcr*-1 to *mcr*-5 and *mcr*-7)	10	*mcr*-1 (7 *K. pneumoniae*) and *mcr*-7 (3 *K. pneumoniae*)	ST15	IncI2	*mcr*-7.1 strain: $bla_{\text{CTX-M-55}}$; *mcr*-1 strain: $bla_{\text{SHV-28}}$, *oqxAB*, *fosA* $bla_{\text{CTX-M-55}}$ and *aph(3')-Ia*	Yang *et al.* (2018)*
Chickens and ducks	2015-2017	1360	*mcr*-1.1 (170 *E. coli*), *mcr*-1.3 and	ST2732, ST2914, ST68, ST501,	IncI2, IncX4, IncFIB, IncFII,	*strA*, *strB*, *aph(3')-Ia*, *aac(3')-IId*, aac(3')-Iva, *fosA3*,	Zhuge *et al.* (2019)**

	(*mcr*-1 to *mcr*-5)		*mcr*-1.7 (1 *E. coli* each)	ST38, ST2085, ST117, ST10, ST1060, ST827, ST131, ST617, ST7153, ST48, ST93, ST58, ST2323, ST155, ST224, ST602, ST167, ST4214, ST302, ST216, ST34, ST540, ST227, ST3014, ST162, ST6257, ST410, ST452, ST3499, ST6484, ST1011, ST95, ST2171, ST648, ST457, ST29, ST294, ST723, ST109, ST678, ST17, ST62, ST3, ST5694, ST101, ST359 and ST127	IncHI2A, IncB/O/K/Z, IncA/C and IncL/M	*catA1*, *catB*, *cmlA1*, *floR*, *sul1*, *sul2*, *dfrA*, *tet*(A), *tet*(B), *tet*(M), *oqxA*, *oqxB*, *qnrS*, *qnrA*, *qepA*, *qnr*, *aac(6')Ib-cr* and *qnrD*	
Chickens	2014-2015 (*mcr*-1)	120	*mcr*-1 (70 *E. coli*)	ST10 and ST156	IncI	bla_{NDM}	Wang *et al.* (2017)*
Ducks	2017 (*mcr*-3 and WGS)	*mcr*-3 detected in 3 cloacal samples	*mcr*-3.10 (1 each of *Aeromonas caviae*, *E. coli* and *Proteus mirabilis*)	*E. coli*: ST457; *A. caviae*: ST513	IncI2 (IS*As13* and IS*Aeca6*)	*E. coli*: *ant(6)-Ia* and *tet(D)*	Wang *et al.* (2018)***
Chickens and environment	2016 (*mcr*-1)	52	*mcr*-1 (22 *E.* coli, 5 *Salmonella*, 1 *Providencia alcalifaciens* and 1 *Enterobacter cloacae*) and *mcr*-1.3 (1 *Rauoltella planticola*)	-	IncX4 and IncI2 (IS*Apl1*)	-	Wang *et al.* (2018)*
Chicken	2016 (*mcr*-1)	1	*mcr*-1 (1 *E. coli*)	ST457	IncFIB, IncFII and IncI2	$bla_{CTX-M-27}$, *aadA2*, *aph(3')-Ia*, strA/B, *rmtB*, bla_{TEM-1B}, *fosA*, *mph(A)*, erm(B), *floR*, *sul1*, *sul2* *tet(A)* and *dfrA12*	Zhang *et al.* (2019)*
Chickens	2013 (*mcr*-1 and *mcr*-2)	168	*mcr*-1 (10 *E. coli*)	ST2018, ST37, ST117, ST359, ST155 and ST3489	IncI2	$bla_{CTX-M-15}$ and bla_{SHV-11}	Wang *et al.* (2017)*
Ducks	2018 (*mcr*-1)	125	*mcr*-1 (1 *Salmonella*	ST399 and ST2529	IncX4 and IncHI2	*aph(6)-Id*, *aph(3'')-Ib*, *aac(6')-Iaa*,	Tang *et al.* (2020)***

			Goldcoast and 1 *Salmonella* Ngor)			*aadA2*, *aac(6')-Iaa*, *aac(3)-IIa*, *aadA5*, *aph(4)-Ia*, *aac(3)-IV*, *aadA5*, *bla*$_{CTX-M-55}$, *bla*$_{CTX-M-14}$, *floR*, *sul1*, *sul2*, *drfA12*, *dfrA17*, *tet(A)*, *oqxB*, *oqXA*, *qnrS1*, *fosA3* and *mph(A)*	
Chickens	2008-2014 (*mcr*-to *mcr*-5)	341	*mcr*-1 (44 *E. coli*)	ST1564, ST1589, ST3041, ST1286, ST648, ST5229, ST617, ST10, ST29, ST46, ST93, ST101, ST156, ST165, ST354, ST533 and ST542	IncI2 (IS*Apl1*)	Tn6330, *bla*$_{TEM-198}$, *bla*$_{TEM-1}$, *bla*$_{CTX-M-55}$, *bla*$_{CTX-M-14}$, bla$_{CTX-M-65}$, bla$_{CTX-M-15}$, *bla*$_{CTX-M-65}$, *bla*$_{OXA-1}$, *bla*$_{OXA-10}$, *bla*$_{TEM-141}$ and *bla*$_{CMY}$	Wu *et al*. (2018)*
Ducks	*mcr*-1 to *mcr*-8	45	*mcr*-1.1 (35 *E. coli*)	ST48, ST7157, ST10, ST220, ST16, ST189, ST226, ST7454, ST93, ST2929, ST2223, ST162, ST156, ST1582, ST155, ST410, ST1011, ST5259, ST648, ST354 and ST770	IncHI2, IncI, IncX4, IncP and Incp0111	*bla*$_{TEM-1}$	Shen *et al*. (2019)**
Chickens	2017-2018 (*mcr*-1)	668	*mcr*-1 (102 *E. coli*)	ST1251, ST219, ST4710, ST10, ST4477, ST93, ST423, ST95, ST224, ST168, ST48, ST1011, ST69, ST101, ST38, ST43, ST602, ST4969, ST206, ST7584, ST359, ST8900, ST156, ST271, ST359, ST1286,ST4753, ST4204, ST354, ST189, ST1266, ST2732, ST4129, ST648,ST617, ST7108,	-	*bla*$_{TEM}$, *bla*$_{CTX-M}$, *bla*$_{PSE}$, *bla*$_{SHV}$, *aac(6)-Ib-cr* and *qnrB*	Zhao *et al*. (2020)***

				ST2847 and ST297			
Chickens	2008 (*mcr*-1 to *mcr*-9)	55	*mcr*-3.6 and *mcr*-3-like (1 *Aeromonas veronii*)	ST514	IS*As2*, IS*As20* and Δ1IS*Ahy2* and Δ2IS*Ahy2*	Tn*6518*, *bla*$_{\text{PER-3}}$, *strA*, *strB*, *aac(6')Ib-cr*, *aacA4*, *mph(A)*, *catB3*, *sul1* and *tetE*	Wang *et al*. (2020)*
Chickens and chicken slaughter environment	2010-2019 (*mcr*-1)	721	*mcr*-1.1 (10 GNB.)	-	-	-	Fan *et al*. (2020)**
Chicken meat and egg	2011-2016 (*mcr*-1)	2	*mcr*-1 (1 *Salmonella* Indiana and 1 *Salmonella* Typhimurium)	ST 17 and ST34	IncHI2 and IncI2; and chromosome	*bla*$_{\text{OXA-1}}$, *bla*$_{\text{CTX-M-65}}$ *bla*$_{\text{TEM-1B,}}$ *aac(3)-Iva*, *aph(4)-Ia*, *bla*$_{\text{CTX-M-14}}$ and *fosA3*	Hu *et al*. (2019a)*; Hu *et al*. (2019b)*
Chickens	2017 (*mcr*-1to *mcr*-4)	130	*mcr*-1 (75 *E. coli*)	-	-	*bla*$_{\text{TEM}}$, *bla*$_{\text{CTX-M}}$, *aphA3*, *tetA*, *tetB*, *tetC*, *sul1* and *sul2*	Song *et al*. (2020)***
Chicken	2019 (*mcr*-1-*mcr*-8)	1 *Klebsiella pneumoniae*	*mcr*-8.2 (1 *Klebsiella pneumoniae*)	ST395	IncFIB (K) (IS*Ecl1*, IS*903B* and IS*Kpn26*	*floR*, *sul1*, *aac(6')-Ib-cr*, *aadA16*, *aadA2*, *aph(3')- Ia*, *bla*$_{\text{CTX-M-27}}$, *bla*$_{\text{DHA-1}}$, *mph*(A), *tet*(D*)*, *dfrA12*, *dfrA27*, *aac(6')-Ib-cr* and *qnrB4*	Yang *et al*. (2020)***
Chickens	2019 (*mcr*-1 to *mcr*-10)		*mcr*-8 (16 *K. pneumoniae*)	ST37 and ST3332	IncFIB/IncFII, IncFIA/IncFIB, IncHI1B and IncFIB/IncHI1B	*tmexCD1-toprJ1*, *bla*$_{\text{CTX-M-27}}$, *bla*$_{\text{DHA-1}}$, *bla*$_{\text{SHV-11/12}}$, *bla*$_{\text{TEM-1B}}$, *floR*, *fosA*, *oqxAB*, *tet*(A), *dfrA27*, *sul1*, *sul3*, *rmtB*, *aac(6')Ib*, *aadA*, *ant(2'')- Ia*, *aph(3'')-Ib*, *aph(6)-Id*,	Wang X et al. (2022)***
Chicken	*mcr*-1 to *mcr*-10	1	*mcr*-1 (1 *E. fergusonii*)	-	-	*bla*$_{\text{TEM-1A}}$, *bla*$_{\text{CTX-M-65}}$, *bla*$_{\text{OXA-1}}$, *bla*$_{\text{TEM-1B}}$, *bla*$_{\text{CTX-M-55}}$, *aac(3)-IId*, *aph(3')-Ia*, aph(3')-Ib, *aph(6)-Id*, *rmtB*, *aac(6')-Ib-cr*, *aadA2*, qnrS2, *oqxA*, *oqxB*, *fosA*, *mph*(A), *floR*, *catA1*, *catB3*, *arr-3*, *sul1*, *sul2*, *dfrA12*, and *tet*(A)	Tang B et al. (2020a)***
Chickens	2010-2011 (*mcr*-1 to *mcr*-9)	210 *E. coli*	*mcr*-1 (77 *E. coli*)	(A, B1, B2 and D) *aafII*, *eae* and *stx1*	IncHI2, IncFrep, IncFIC, IncX4, IncFIA, IncP, IncN, IncY and IncA/C (IS*Ecp1,* IS*CR1* and IS*Apl1*)	*bla*$_{\text{TEM}}$, *bla*$_{\text{SHV}}$, *bla*$_{\text{CTX-M-1}}$, *bla*$_{\text{CTX-M-9}}$, *bla*$_{\text{CTX-M-55}}$, *bla*$_{\text{CTX-M-14}}$, *bla*$_{\text{CTX-M-15}}$, *bla*$_{\text{CTX-M-28}}$, *bla*$_{\text{CTX-M-66}}$ and integrons	Shafiq M. et al. (2021)*
Chickens and ducks	2019 (*mcr*-1 to *mcr*-10)	104 *E. fergusonii*	*mcr*-1 (24 *E. fergusonii*) from chickens and ducks		IncI2, IncHI2 and IncHI2A(IS*Apl1*)	*bla*$_{\text{TEM-1B}}$, *floR*, *bla*$_{\text{CTX-M-65}}$, *bla*$_{\text{OXA-10}}$, *bla*$_{\text{CTX-M-3}}$, *bla*$_{\text{TEM-1A}}$, *fosA3*, *bla*$_{\text{CTX-M-137}}$, *bla*$_{\text{CTX-M-14}}$, *bla*$_{\text{CTX-M-55}}$ and mutation in *gyrA*	Tang B. et al. (2020b)***

Ducks	2020-2021 (*mcr*-1 to *mcr*-10)	54 *E. fergusonii*	*mcr*-1 (4 *E. fergusonii*)	*gad*, *ireA*, *iss*, *iucC*, *iutA*, *papA20*, *papC*, *terC*, *traT* and *Irea*	IncI2	*aac(3)-IV*, *aadA1*, *aadA2B*, *aph(3)-Ib*, *aph(4)-Ia*, *aph(6)-Id*, bla_{OXA-10}, *qnrS1*, *lnu*(F), *cmlA1*, *floR*, *arr-2*, *sul2*, *sul3*, *dfrA14*, *tet*(A) and *tet*(B)	Lin J et al. (2022)***
Chicken meat	2017 (*mcr*-1 to *mcr*-10)	2	*mcr*-1 (2 *E. coli*)	ST83 and ST21	IncHI2 and IncI2 (IS*26s*; IS*186B* and class one integron)	bla_{NDM-5}, *mph*(A), *tet*(M), *floR* and *aph(39)-Ia*	Liu X. et al. (2021)***
Chickens	2016-2018 (*mcr*-1 to *mcr*-9)	37	*mcr*-1 (12 *E. coli*)	ST10, ST206 ST48 ST155 ST542 ST2539 ST196, ST90, ST2253, ST871, ST73, ST4976, ST3494, ST641, ST410, ST1147, and ST1141. STs of chicken strains were not indicated	-	bla_{SHV}, *aac(6')-Ib-cr*, *qepA*, *qnrS*, *oqxA*, *oqxB*, *sul1*, *sul2*, *sul3*, bla_{TEM}, bla_{CTX-M}, *aacC2*, *aacC4*, *strA*, *tetA*, *tetB*, *tetC* and *floR*	Li F. et al. (2022)*
Eggs and pickled ready-to-eat meat	2007-2016 (*mcr*-9)	2027 Samonellae	*mcr*-9 (127 *Salmonella*)	-	-	bla_{TEM-1} and $bla_{CTX-M-3}$	Sheng et al. (2022)*
Chicken and duck meats	2017 (*mcr*-1 to *mcr*-10)	Salmonell lae	*mcr*-1.1 (1 *Salmonella*) and *mcr*-9 (4 *Salmonella*)	*fimD* and *pefC*	IncX4 and IncI, IncHI2A	*aac(6')-Iaa*, *aph(300)-Ib*, bla_{TEM-1} and *sul2*	Lyu et al. (2021)***
Chicken meats	2015-2017 (*mcr*-1 to *mcr*-4)	315 *E. coli*	*mcr*-1 (34 *E. coli*)	=	IncI2 and IncHI2	bla_{NDM-1}	Liu X. et al. (2019)***
Chickens and ducks	(2017) *mcr*-1	27 *E. coli*	*mcr*-1 (27 *E. coli*)	ST767, ST155, ST457, ST48, ST4204, ST533, ST1638, ST 101, ST6395, ST4408, ST1968, ST 117 and ST156	-	$bla_{CTX-M-14}$, $bla_{CTX-M-15}$, bla_{TEM-1}	Li X. et al. (2020)***
Chickens	*mcr*-1	78 *E. coli*	*mcr*-1 (53 *E. coli*)	ST297, ST156, ST2973, ST2847, ST117, ST101, ST617, ST10, ST1011 and ST2944	IncI2 and IncHI2/ST3-type	$bla_{CTX-M9G}$, $bla_{CTX-M1G}$, *rmtB*, bla_{NDM-5}, bla_{NDM-1}, *floR* and *fosA3*	Liu B-T et al. (2017)***
Geese	2017 (*mcr*-1 to *mcr*-10)	162 *Enterobacteriaceae*	*mcr*-1 (1 *E. coli*)	-	Chromosomal *mcr*-1	*qnrS1*, bla_{TEM-1B}, *aph(6)-Id*, *aph(3')-Ib*, *sul2*, *dfrA14*, *floR* and *tet*(A) (IS *Apl1*)	Lu X et al. (2019)***
Chicken and chicken	2019-2020	690 samples	*mcr*-1 (17 E. *coli*)	ST10338, ST1403, ST1421,	-	bla_{CTX}, bla_{OXA}, bla_{TEM}, $bla_{TEM-122}$, *tet*(A), *floR*, *qnrS1*,	Hu et al. (2022)***

farm environment (water, litter and soil) and flies in farm environment	(*mcr*-1 to *mcr*-10)			ST156, ST162, ST3941, ST6321, ST69, ST7153, ST93, and ST12735 (A, B1 and D)		*dfrA10*, *dfrA14* and *sul1*	
Live poultry markets	2015-2017 (*mcr*-1 to *mcr*-9)	926 *E. coli*	*mcr*-1 (157 *E. coli*)	ST362, 117, 2944, (*KpsM II*, *iutA* an *papA*)	-	$bla_{CTX\text{-}M\text{-}9G}$, *fosA3*, *rmtB*, *floR*, $bla_{NDM\text{-}5}$, $bla_{CTX\text{-}M\text{-}1G}$ and *oqxAB*	Zou et al. (2021)**
Chickens, slaughterhouse workers and people nearby	2019 (*mcr*-10)	200 *Enterobacterales*	*mcr*-10.1 and *mcr*-8 (2 *K. pneumoniae* from chicken, 1 *E. coli* from slaughterhouse and 1 *Enterobacter kobei* from nearby person*)*	ST216; *K. pneumoniae*: ST11 *Enterobacter*: ST1605,	IncFIB, IncFII and IncFIA	*Klebsiella*: $bla_{ACT\text{-}9}$, *aph(3)-lb*, *aph(6)-lb*, *aadA16*, *aac(3)-IId*, *aac(6)-Ib-cr*, *arr-3*, *mph(A)*, *dfrA27*, *sul1*, *sul2*, *tet*(A), *qacE*, *qnrB52*, $bla_{TEM\text{-}1B}$, *floR*, *qnrS1*, $bla_{CTX\text{-}M\text{-}15}$, $bla_{TEM\text{-}1B}$, *aph(39)-Ia*, *aph(39)-Ib*, *aph(39)-IId*, *aadA16*, *aph (6)-Id*, *aph(6)-Id-cr*, *tet*(A), *mph*(A), *aac(69)-Ibcr*, *afrA27*, *msr*(E), *mph*(E), *qacE*, *cmlA1*, *qnrB52*, *sul1*, *tet*(A), $bla_{TEM\text{-}1B}$, *floR*, *fosA*, *oqxA*, *oqxB* and $bla_{SHV\text{-}182}$	Xu et al. (2022)***
Chickens	2016-2019 (mcr-1 to *mcr*-10)	53 samples	*mcr*-1 (10 *E. coli*)	ST156, ST5454 and ST602 (*cvaC*, *etsC*, *hlyF*, *iroN*, *iss*, *iucC*, *iutA*, *mchF*, *ompT*, *sitA*, *traT*, *gad*, *hra*, *lpfA*, *papA_F19*, *terC*, *astA*, *gad*, *lpfA*, *papC*, *terC* and *cma*)	IncX4 and IncI2	$bla_{TEM\text{-}1B}$, *mdf*(A), gyrA, *aadA2*, *aac(3)-IId*, *aph(3')-Ia*, *oqxA*, *oqxB*, *dfrA12*, *sitABCD*, *mph*(A), $bla_{CTX\text{-}M\text{-}55}$, *aph(6)-Id*, *aph(3'')-Ib*, *tet*(A), *sul2*, *floR*, *fosA3*, $bla_{NDM\text{-}5}$, *aadA1*, *dfrA1*, *fosA7*, *mdf*(A), *dfrA17* and mutation in *gyrA*	Liu Z et al. (2021)**
|Chickens	*mcr*-1 to *mcr*-10	8	*mcr*-1 (4 *E. coli*)	ST189 (*gad*)	IncX3	$bla_{CTX\text{-}M\text{-}65}$, $bla_{OXA\text{-}10}$, $bla_{NDM\text{-}5}$, $bla_{CTX\text{-}M\text{-}65}$, $bla_{OXA\text{-}10}$, *aph(4)-Ia*, *aac(3)-IVa*, *fosA*, *aac(6')-Ib-cr*, *aph(3')-Ia* and *mph*(A)	Liu Z. et al. (2022)
Chickens	1970-2019 (*mcr*-1 to *mcr*-10)	567 *E. coli*	*mcr*-1 (251 *E. coli*)	Many up to 21	NA	*aac(3)-IIa*, *aac(3)-Iva*, *aac(6')-Ib-cr*, *aph(3")-Ia* , *aadA*, *aadA5*, *aph(4)-Ia*, *qnrS*, *aphA2*, *oqxB*, *oqxA*, *erm*(B), *erm*(C), *lnu*(F), *mph*(A) , $bla_{CTX\text{-}M\text{-}1}$, $bla_{CTX\text{-}M\text{-}9}$, $bla_{NDM\text{-}1}$, $bla_{OXA\text{-}1}$, bla_{CMY},	Yang L. et al. (2022)**

						bla_{TEM-1D}, *ampC2*, *ampC1*, *strB*, *strA*, *sat2a*, *rmtB*, *fosA*, *catA1*, *catB3*, *cmlA*, *floR*, *arr*, *sul1*, *sul2*, *sul3*, *tet*(A), *tet*(B), *tet*(M), *dfrA1*, dfrA27, *dfrA5*, *dfrA7* and *dfrA12*	
Pigeons	*mcr*-1 to *mcr*-10	3	*mcr*-1 (3 *E. coli*)	ST6775 (*gad*, *neuC*, *terC* and *traT*)	Chromosomal *mcr*-1, (Tn*633*)	bla_{NDM-5}, *tet*(X4), *aadA1*, *aadA2*, *aph(39)-Ia*, *aac(3)-IId*, *floR*, cmlA1, *mdf*(A), *tet*(A), *sul2*, sul3, *dfrA12*, *erm(42)*, and *mef*(B)	Lu X et al. (2022)***
Chickens	2013-2016 (*mcr*-8)	26	*mcr*-8.1 *and novel mcr*-8.5 (7 *K. pneumoniae*) and *mcr*-3.11 (1 *K. pneumoniae*)	ST37	IncFIA, IncFII(K), IncFII and IncFIB (IS*Apl1*)	*tmexCD1-toprJ*	S. Sun et al. (2020)*
Chicken			novel *mcr*-8 (*K. pneumoniae*)				Wang et al. (2018)
Chickens	2013 (*mcr*-1)	58	*mcr*-1 (53 *E. coli*)		IncHI2 and IncI2 (IS*Apl1)*	$bla_{CTX-M-14}$, $bla_{CTX-M-64}$, *fosA3*, $bla_{CTX-M-55}$, $bla_{CTX-M-65}$, *floR*, $bla_{CTX-M-82b}$ and *rmtB*	Cao et al. (2020)*
Chicken	2016 (*mcr*-1 to *mcr*-10)	1	*mcr*-1 (1 *E. coli*)	ST93	IncI2	$bla_{CTX-M-55}$, bla_{TEM-1b}, *rmtB*, $bla_{CTX-M-14}$, *fosA3*, *floR*, *cmlA1*, *aac(3)-IVa*, Δ*aac(3)-IIa*, *aph(4)-Ia*, *aph(3')-Ia*, *ant(3')-Ia*, *ant(3")* family, *sul2*, *sul3*, *dfrA12*, *qacL*, *mef*(B), and *lnu*(F)	Li et al. (2021)*
Chicken egg	2016 (*mcr*-1)	755 *Salmonella*	*mcr*-1.19 (1 *S.* Typhimurium)	ST34	InHI2 (IS*Apl1)*	bla_{TEM-1B}, $bla_{CTX-M-14}$, *fosA3* and mutation in *gyrA*	Hu et al. (2021)*
Chicken	2019	1	*mcr*-10.1 (*Enterobacter rogenkampii*)	ST1056	IncFII	bla_{MIR} and *fosA*	Lei et al. (2021)***
Chickens	2014-2019 (*mcr*-1 to *mcr*-4)	131	*mcr*-1 (51 *E. coli*)	ST696, ST162, ST156, ST10, ST226, ST3519, ST359, ST2179, ST124, ST48, ST6706, ST175, ST6740, ST65, ST189, ST1296, ST4935, ST612, ST171, ST7115, ST117, ST1158, ST2732, ST354, ST82 and ST7189	IncN, IncHI2, IncI1, IncFIB, IncP, IncX4, IncY, IncFrep, IncX2, and IncFIA	-	Zhang W. et al. (2022)***

Geese	*mcr*-1 to *mcr*-10	33 *K. pneumoniae*	*mcr*-1 (1 *K. pneumoniae*)	*ent*B,C,E,F,S, *acrB*, *fepA, D,G*, *etsC*, *altB*, *ompA*, *gndA*, *galF*, *rpos*, *ugD* and *rfBK*	-	bla_{NDM-5}, bla_{CTX-M}, *aad*, *oqxB* and *tet*	Bai *et al*. (2022)
Poultry meat	*mcr*-1 to *mcr*-10	2	*mcr*-1 (1 *E. coil*)	-	IncHI	bla_{OXA-1}, $bla_{CTX-M-65}$, bla_{TEM-1B}, *aac(3)-IV*, *aadA22*, *tet*(A), *tet*(M), *catB3*, *floR*, *sul1*, *sul2*, *aac(6')-Ib-cr*, *oqxA*, *oqxB* and *qnrS2*	Wang *et al*. (2022)
Pigeons	2017 and above (Mcr-1 to mcr-10)	100	*mcr*-1 (45 *E. fergusonnii*)	ST646, ST155, ST38, ST224, ST8024, ST7153, ST939, ST648, ST2351, ST6164 and ST6775	IncX4, IncI2, IncI1, IncHI1, IncY, IncFIB, IncF, IncN	*tet*(X4)	Lu *et al*. (2023)
Ducks and geese	2019-2020 (*mc*r-1 to *mcr*-10)		*mcr*-1 (57 E. *coli*)	-	IncHI2, IncX4, IncI2, IncFIB, IncFIA, IncFIC, IncFII and ncHI2A	$bla_{CTX-M-55}$, $bla_{CTX-M-65}$, bla_{TEM-1}, bla_{OXA-10}, bla_{OXA-1}, $bla_{CTX-M-123}$, *fosA3*, *floR*, *tet*(A) and bla_{TEM-1}	Hu *et al*. (2022)***
Poultry birds and farmworkers	2019 (*mcr*-1 to mcr-10)	5	*mcr*-8.1 (3 *Klebsiella pneumoniae*)	*fimABCDEFGHI*, *mrkABCDFHIJ*, *entABCDEF*, *fepABCDG* and *iroE*	IncFII(pKP91)/IncFIA(HI1)	bla_{NDM-5}, *armA*, bla_{TEM-1B}, $bla_{CTX-M-1}$, *ble*O, *oqxAB qnrS*, *qnrB*, *aac(6')-Ib-cr* , *gyrA* and *parC*	Yang *et al*. (2022)***
Chicken eggs	2017-2022 (*mcr*-1 to *mcr*-10)	22	*mcr*-1 (7 *Salmonella*)	-	-	*aph(4)-Ia*, *aac(3)-IV*, *aph(6)-Id*, *aac(60)-Iaa*, *aph(3")-Ib)*, bla_{OXA-1}, bla_{TEM-1B}, *aac(6')-Ib-cr* and *gyrA* catB3, *arr-3*, *sul1*, *sul2*, *aph(30)-Ia*, *aadA1*, *aadA2b*, *sul3*, *oqxAB*, *floR*, *dfrA12*, *cmlA1* and *ter*YXWZABCDEF	Li et al. (2022)***
Chickens	2020-2021 (*mcr*-1 to *mcr*-10)	54	*mcr*-1 (4 *E.* fer*gusonii*)	*gad*, *ireA*, iss, *iucC*, *terC*, *traT*, *iutA* and *papA*	InI2 (IS*Apl1*, IS*609*, IS*683*, IS*200* and IS*Ecp1* and IS*2*)	bla_{OXA-10}, *qnrS1*, *lnu*(F), *cmlA1*, *floR*, *ARR-2*, *sul2*, *sul3*, *dfrA14*, *tet*(A) and *tet(*B)	Lin *et al*. (2023)***
Chicken meats	2019-2021 (*mc*r-1 to *mcr*-10)	455	*mcr*-1 (7 *Salmonella* Indiana)		IncN1-IncHI2 and IncX4 (IS26, IS1, IS*4321*, IS1006, ISCR2, IS*Aba1*,	*aph(4)-Ia*, *aphA1*, *aac(3)-IVa*, *aadA1*, *aadA2*, *aadA5*, *cmlA1*, *floR*, *oqxAB*, *sul2*, *sul3*, *mph*(A), *dfrA17*, *ter*YXWZABCDEF	Mei *et al*. (2023)***

					Tn*5393*, and IS*Ec59*)		
Chickens	2020-2021 (*mcr*-1 to *mcr*-10)	346	*mcr*-1 (12 *E. coli*)	IncI2 and IncHI2	ST29735, ST43, ST10, ST93, ST162, ST2329, ST189, ST4689, and ST259 (terC and astA,	*aac(3)-IIa*, *aac(3)-IV*, *aadA1*, *aadA2*, *aadA5*, *aadA16*, *aadA22*, *aadA24*, *aadA2b*, aph(3′)-Ia, aph(3′)-IIa, *aph(3″)-Ib*, *aph(4)-IA*, *aph(6)-Id*, *rmtB*, $bla_{CTXM-55}$, $bla_{CTX-M-64}$, $bla_{CTX-M-65}$, bla_{OXA-10}, $bla_{TEM-141}$, $bla_{TEM-206}$, $bla_{TEM-214}$, bla_{TEM-1B}, bla_{TEM-1C}, *dfrA12*, *dfrA14*, *dfrA17*, *dfrA27*, *sul1*, *sul2*, *sul3*, *oqxA*, *oqxB*, *qnrS1*, *qnrS2*, *lnu*(F), *mph(A)*, cmlA1, *floR*, *fosA3*, *arr-2*, *arr-3*, *tet*(A) and *tet*(M)	Tang et al. (2022)***

mcr: mobile colistin resistance gene; NA: not publicly-available; *: study on preban (2016 downwards) isolates; ***study on postban (2017 upwards) isolates; **: study on isolates collected preban and postban; PMCR: plasmid-mediated colistin resistance; GNB: Gram-negative bacteria; -: no data; Additional resistance traits: resistance factors identified in one *mcr*-positive isolate or pooled factors in more than one *mcr*-positive isolate; Sequence type: all sequence types of *mcr*-positive isolates; ST: sequence type; Plasmid: plasmid types identified in one or pooled *mcr*-positive isolates; Inc.: incompatibility; IS: insertion sequence, Δ: truncated; ESBL: Extended-spectrum β-lactamase; AmpC: Ampicillinase C
